# Childhood predictors of religious reading: a cross-national analysis in the Global Flourishing Study

**DOI:** 10.1038/s41598-025-10142-7

**Published:** 2025-07-10

**Authors:** Dorota Weziak-Bialowolska, Richard G. Cowden, Piotr Bialowolski, Matt Bradshaw, R. Noah Padgett, Byron R. Johnson, Tyler J. VanderWeele

**Affiliations:** 1 Department of Quantitative Methods & Information Technology, Kozminski University, Warsaw, Poland; 2 Human Flourishing Program, Institute for Quantitative Social Science, Harvard University, Cambridge, MA 02138 USA; 3 Department of Epidemiology, Harvard T.H. Chan School of Public Health, Boston, MA USA; 4 Department of Economics, Kozminski University, Warsaw, Poland; 5 Institute for Studies of Religion, Baylor University, Waco, TX USA; 6 School of Public Policy, Pepperdine University, Malibu, CA USA

**Keywords:** Religiosity, Religious reading and listening, Childhood, Adolescence, Cross-cultural, Sacred texts, Religious literature, Psychology, Human behaviour

## Abstract

While the family environment, including parental religiosity, shapes children’s religious behaviors, evidence concerning factors influencing religious reading and listening (RR/L) in adulthood remains scarce. This study examines 13 early-life conditions within familial and economic contexts as potential predictors of adult RR/L. Nationally representative samples from 22 countries (*N* = 202,898) included in the first wave of the Global Flourishing Study were used. Multiple imputation, multivariate regression analyses, and random-effects meta-analyses were employed to evaluate associations between the childhood predictors and adult RR/L. Meta-analytical results show that early-life predictors, including religious service attendance, a positive relationship with one’s father, excellent self-assessed health, experience of abuse, and growing up as an outsider, as well as belonging to older cohorts and being female are associated with higher likelihood of adult RR/L, with religious service attendance being the strongest predictor. There was little evidence of associations with childhood financial status, immigration status, and mother-child relationship. Country-specific analyses revealed that race/ethnicity and religious affiliation also influence the RR/L likelihood, with Muslims and minority group members showing a higher probability compared to other groups. Given the rare examination of early-life determinants of RR/L, especially in cross-cultural contexts, this study provides valuable insights into the global distribution of early-life predictors of RR/L.

**Supplementary Information:**

The online version contains supplementary material available at 10.1038/s41598-025-10142-7.

## Introduction

According to recent global estimates, over 80% of the world’s population identifies with a religious group^1^ with more than 20% of Americans and about 30% of the Global Flourishing Study (GFS) population regularly engaging in private religious practices such as scripture reading^2,3^. Despite this, the specific practices of religious reading and listening (RR/L) remain relatively understudied, particularly across non-Western and multifaith contexts. Most existing research has centered on religious service attendance and prayer, with much less attention paid to private, non-institutional expressions of religiosity that often serve as sources of spiritual growth, provide moral guidance, and ensure psychological comfort.

RR/L is a distinct component of private religious practice. Unlike organized worship or institutional rituals, these practices are typically informal, self-directed, and occur in individual or family settings rather than organized worship environment^4^. People engage with sacred texts to seek closeness to the divine, to find direction during adversity, or to fulfill religious obligations. Existing limited empirical evidence and theoretical arguments suggest, however, that these sacred scriptures could also influence their readers’ lives at spiritual, psychological, and moral levels^5–8^.

Despite their spiritual significance, scholarly attention to RR/L has been limited^8^. Most existing research has focused on Christian populations and primarily examined Bible reading, leaving listening to sacred texts and engagement with sacred scriptures that are relevant to other religions and faiths (such as the Tanakh for Jews, the Quran for Muslims, the Vedas for Hindus, the Tripitaka and the Mahayana Sutras for Buddhists, and the Tao Te Ching for Taoists, among others) relatively neglected. Furthermore, prior research on religiosity is disproportionately concentrated in Western, Educated, Industrialized, Rich, and Democratic (WEIRD) societies, potentially overlooking other cultural contexts that might be important for developing a more globally representative picture of how religiosity might impact human life. When a comparative perspective has been adopted, it has often been limited to a group of European countries^9–11^. While data scarcity likely influenced this focus, more comparative, country-level evidence is needed. Finally, evidence from a lifespan perspective is limited. Evidence has been documented for associations involving religiosity during childhood or adolescence, such as the relation between Bible reading and purpose in life among 13–15-year-olds^7^ or the effects of adolescent religiosity on adult health and well-being^12^. However, relatively little is known about how early-life experiences contribute to adult RR/L.

This study aims to examine the early-life determinants of RR/L in adulthood to provide insights into which aspects of a childhood environment contribute to promoting RR/L in adulthood. To this end, associations between the childhood experiences, personal attributes, and familial or social conditions at the age of 12 years and adult engagement in reading and listening to sacred texts are examined within cross-national and cross-cultural contexts. This retrospective and multi-contexts approach offers further insights into mechanisms that may cultivate RR/L. It could also shed light on the role of broader societal factors in shaping the relationship between childhood experiences and adult outcome. Consequently, we hypothesize that among 13 examined childhood predictors (including childhood experiences, personal attributes, and familial or social conditions as well as demographic characteristics), certain predictors will demonstrate meaningful associations with an individual’s religious reading and listening in adulthood. Additionally, the strength of these associations between the 13 childhood predictors and RR/L in adulthood will differ by country reflecting the diverse sociocultural, economic, and health contexts that characterize each nation. To evaluate these hypotheses, data from a diverse sample of 202,898 individuals from 22 countries participating in the first wave of the Global Flourishing Study (GFS) were used.

## Early-life predictors of religiosity

Childhood is a formative period in which moral values, norms, and belief system begin to take shape, guiding individual attitudes and behaviors throughout their lifes^13–15^. These early years are critical not only for psychological and physical development but also for the emergence of religious and spiritual identities^16,17^. Family structures, emotional bonds, and social environments encountered during childhood can leave lasting imprints on adult religiosity, including the practice of RR/L.

Theoretical frameworks help illuminate how early-life experiences may influence religiosity. Bronfenbrenner’s ecological systems theory^18^ emphasize the dynamic and layered influence of individual, familial, and sociocultural contexts on child’s development. Early exposure to religious practices, whether through parental modeling, religious communities’ norms, or broader cultural norms, can lay the foundation for enduring religious behaviors. Parallelly, personal experiences such as psychological, social or economic adversity may either hinder or deepen religious engagement, depending on how individuals interpret and respond to these experiences. Attachment theory further underscores that the role of early caregiver relationships in shaping both emotional security and perceptions of the divine^19–21^. Secure attachments to parents, fostered by consistent emotional support, open communication, and sensitive responsiveness to child’s needs, may promote a benevolent view of God as nurturing and reliable, aligning early relational experiences with adult religiosity through the correspondence pathway. In contrast, insecure attachments to parents, often consequential of parents’ emotional unavailability or dismissiveness, may lead individuals to seek religiosity as a compensatory mechanism to address emotional distress (the compensation pathway). Social learning theory^22^ also highlights the importance of observing religious behaviors, such as prayer, scripture reading, or attending religious services, within the family and community contexts, which may encourage similar practices in later life. Other frameworks, such as resilience theory^23^ and the theory of religious coping^24,25^ suggest that religiosity may serve as a psychological resource in response to childhood adversity. Experiences such as childhood abuse, neglect, or marginalization may lead individuals to seek comfort and meaning through religion and sacred texts. Relative deprivation theory^26,27^ adds that individuals from disadvantaged backgrounds may turn to religiosity, including RR/L, to cope with material hardship and envision religiosity as a path to more hopeful future. Taken together, these frameworks suggest that supportive early-life environment – including stable living conditions, nurturing family relationships, and regular exposure to religious practices, may foster the development of private religious engagement in adulthood. Conversely, adversity in childhood may trigger more complex religious trajectories, including intensified religiosity as a coping mechanism or the emergence of spiritual struggle.

Empirical evidence aligns with these theories. A religious upbringing and exposure to parental religious behaviors (e.g., service attendance, discussions about faith, religious identity) are positively associated with the likelihood of similar religious beliefs and practices later in life^28–32^. Religious education has been also identified as a contributor to adult religiosity^33^. Supportive family environments, particularly those involving positive parental relationships, are linked to more frequent religious practices and greater religiosity in later life^28,34,35^.

Adverse childhood experiences may also shape religiosity, albeit in more complex ways. For example, childhood abuse has been associated with both negative and positive religious outcomes in adulthood—on the one hand, with religious struggle^36,37^, and on the other, with seeking comfort through religion/spirituality, such as an increased positive religious coping^38^. Likewise, while lower socioeconomic status in childhood has been linked to stronger religious beliefs, it does not necessarily predict more frequent religious participation^28^. Additionally, persistent financial stress in adulthood has been prospectively associated with divine religious struggle^27^offering insights into how economic adversities in childhood might influence later spiritual life.

Socio-demographic characteristics also predicts religious engagement. Older adults generally report higher levels of religiosity than younger cohorts, reflecting cohort effects as well as life course changes^39,40^. Women consistently report stronger religious inclinations then men^41,42^. Immigration status and ethnic background can further influence religious trajectories in nuanced ways^43^. Religion may be used to construct personal identity, preserve ethnic heritage, or integrate into a new cultural environment, depending on the resources and context of the host country. Immigrant religious communities often serve protective functions, particularly for second-generation immigrants, although these individuals are often less religious than their parents. The degree to which religiosity is retained or adapted may depend on parental beliefs, country of origin, and the religious landscape of the receiving country, as suggested in previous research^17,44^.

Despite the well-documented associations between childhood experiences, socio-demographic characteristics and adult religiosity, few studies have specifically examined RR/L as an outcome, especially from a global perspective. This study addresses this gap in literature by evaluating 13 early-life predictors of adult RR/L, encompassing family relationships, personal characteristics, and broader childhood contexts.

## Results

The GFS comprises a diverse sample of 202,898 individuals from 22 countries, ensuring broad geographic and cultural representation. Descriptive statistics for the observed sample are presented in Table 1, with each country’s sample weighted to be nationally representative of the demographic characteristics of that country. Summarizing across countries, while participants spanned a wide age range, those born between 1983 and 1993 made up the largest single age cohort (20%). Gender distribution was nearly equal, with slightly more females than males. The vast majority of respondents were born in their respective countries. Childhood experiences varied, with a predominant trend of positive relationships with both mothers and fathers, along with a significant portion reporting comfortable childhood income levels. Nevertheless, 24% of respondents reported being from familial backgrounds characterized by either challenging or highly challenging financial circumstances. Additionally, 14% of participants disclosed instances of childhood abuse, while an equivalent percentage reported experiences of social exclusion during their formative years. Finally, a sizable proportion attended religious services weekly during childhood.

**Table 1:** Nationally-representative descriptive statistics of the observed sample (*N* = 202,898).

Variable	Proportion	Frequency
*Demographic variables*		
Age		
18–24 (born 1998–2005)	0.13	27,007
25–29 (born 1993–1998)	0.10	20,700
30–39 (born 1983–1993)	0.20	40,256
40–49 (born 1973–1983)	0.17	34,464
50–59 (born 1963–1973)	0.16	31,793
60–69 (born 1953–1963)	0.14	27,763
70–79 (born 1943–1953)	0.08	16,776
80 or older (born 1943 or earlier)	0.02	4119
Missing	0.00	20
Gender		
Male	0.49	98,411
Female	0.51	103,488
Other	0.00	602
Missing	0.00	397
Immigration status		
Born in this country	0.94	190,998
Born in another country	0.05	9791
Missing	0.01	2110
*Childhood-related variables*		
Relationship with mother		
Very good	0.63	127,836
Somewhat good	0.26	52,439
Somewhat bad	0.05	11,060
Very bad	0.02	4642
Not applicable	0.03	5965
Missing	0.00	956
Relationship with father		
Very good	0.53	107,742
Somewhat good	0.27	55,714
Somewhat bad	0.08	15,807
Very bad	0.04	8278
Not applicable	0.07	13,985
Missing	0.01	1372
Parent marital status		
Married	0.75	152,001
Divorced	0.09	17,726
Never married	0.08	15,534
One or both had died	0.04	7794
Missing	0.05	9843
Childhood income		
Lived comfortably	0.35	70,861
Got by	0.41	82,905
Found it difficult	0.18	35,852
Found it very difficult	0.06	12,606
Missing	0.00	674
Childhood abuse		
Yes	0.14	29,139
No	0.82	167,279
Missing	0.03	6479
Outsider		
Yes	0.14	28,732
No	0.84	170,577
Not applicable	0.01	2712
Missing	0.00	877
Childhood health		
Excellent	0.33	67,121
Very good	0.31	63,086
Good	0.23	47,378
Fair	0.10	19,877
Poor	0.02	4906
Missing	0.00	530
Childhood service attendance		
At least 1/week	0.41	83,237
1–3/month	0.16	33,308
<1/month	0.18	36,928
Never	0.23	47,445
Missing	0.01	1980
*Country*		
Argentina	0.03	6724
Australia	0.02	3844
Brazil	0.07	13,204
Egypt	0.02	4729
Germany	0.05	9506
Hong Kong	0.01	3012
India	0.06	12,765
Indonesia	0.03	6992
Israel	0.02	3669
Japan	0.10	20,543
Kenya	0.06	11,389
Mexico	0.03	5776
Nigeria	0.03	6827
Philippines	0.03	5292
Poland	0.05	10,389
South Africa	0.01	2651
Spain	0.03	6290
Sweden	0.07	15,068
Tanzania	0.04	9075
Turkey	0.01	1473
United Kingdom	0.03	5368
United States	0.19	38,312

Country-specific descriptive statistics are available in Tables 1a–22a in the Supplementary Material.

The meta-analysis indicated that the strongest effects were observed for the association between religious service attendance at age 12 and adult RR/L. The risk ratio (RR) was 2.56 (95% CI 1.83, 3.63) for those attending religious services at least once per week, RR = 2.05 (95% CI 1.48, 2.84) for those attending 1–3 times per month, and RR = 1.28 (95% CI 1.09,1 .49) for those attending less than once a month, compared to individuals who reported no religious service attendance (Table 2). For this predictor, the share of countries with risk ratios above 1.1 was notably large, indicating rather homogenous, positive, and at least moderately strong effects across nations.

**Table 2:** Random effects meta-analysis of regression of reading sacred texts on childhood predictors.

Variable	Category	RR	95% CI	Estimated proportion of effects by threshold			
				< 0.90	> 1.10	I^2^	Global *p* value
Relationship with mother	(Ref: Very bad/somewhat bad)						0.796
	Very/somewhat good	1.01	(0.97, 1.05)	0.00	0.00	< 0.1ǂ	
Relationship with father	(Ref: Very bad/somewhat bad)						0.168
	Very/somewhat good	1.07	(1.02, 1.12)	0.00	0.41	36.6	
Parent marital status	(Ref: Parents married)						< 0.001**
	Divorced	1.02	(0.93, 1.13)	0.18	0.23	85.0	
	Single, never married	1.10	(0.97, 1.26)	0.27	0.36	89.7	
	One or both had died	0.99	(0.89, 1.09)	0.23	0.14	73.1	
Subjective financial status of family growing up	(Ref: Got by)						0.006*
	Lived comfortably	1.00	(0.97, 1.03)	0.00	0.00	42.4	
	Found it difficult	1.00	(0.97, 1.04)	0.00	0.05	45.7	
	Found it very difficult	1.01	(0.91, 1.11)	0.27	0.23	83.8	
Abuse	(Ref: No)						< 0.001**
	Yes	1.08	(1.01, 1.15)	0.05	0.43	79.1	
Outsider growing up	(Ref: No)						< 0.001**
	Yes	1.10	(1.00, 1.21)	0.09	0.41	91.5	
Self-rated health growing up	(Ref: Good)						< 0.001**
	Excellent	1.07	(1.03, 1.13)	0.00	0.36	66.9	
	Very good	1.05	(0.99, 1.11)	0.09	0.32	78.6	
	Fair	0.95	(0.88, 1.03)	0.32	0.09	79.0	
	Poor	1.13	(0.94, 1.35)	0.18	0.55	90.3	
Immigration status	(Ref: Born in this country)						< 0.001**
	Born in another country	1.06	(0.94, 1.18)	0.18	0.45	68.0	
Age 12 religious service attendance	(Ref: Never)						< 0.001**
	At least 1/week	2.56	(1.81, 3.63)	0.00	0.95	98.7	
	1–3/month	2.05	(1.48, 2.84)	0.00	0.82	98.1	
	Less than 1/month	1.28	(1.09, 1.49)	0.14	0.64	89.7	
Year of birth	(Ref: 1998–2005; age 18–24)						< 0.001**
	1993–1998; age 25–29	1.03	(0.98, 1.08)	0.00	0.18	56.3	
	1983–1993; age 30–39	1.05	(0.96, 1.14)	0.18	0.32	90.3	
	1973–1983; age 40–49	1.08	(0.99, 1.18)	0.14	0.41	87.8	
	1963–1973; age 50–59	1.16	(1.03, 1.30)	0.18	0.68	91.9	
	1953–1963; age 60–69	1.17	(1.02, 1.34)	0.23	0.73	91.8	
	1943–1953; age 70–79	1.23	(1.05, 1.44)	0.14	0.77	86.0	
	1943 or earlier; age 80+	0.73	(0.20, 2.75)	0.18	0.64	99.4	
Gender	(Ref: Male)						< 0.001**
	Female	1.09	(1.00, 1.18)	0.14	0.50	95.4	
	Other	0.02	(0.00, 0.25)	0.61	0.33	99.9	

*p < .05; **p < .004 (Bonferroni corrected threshold); ǂestimate of heterogeneity is likely unstable, please see our online supplement forest plots for more detail on heterogeneity of effects. RR = risk ratio; CI-confidence interval; I^2^ is an estimate of the variability in proportions due to heterogeneity across countries vs. sampling variability which is not uncommonly nearly 100% when there is nice precision in estimated proportion within country; and the Global *p* value corresponds to a test of the null hypothesis that there are no differences between the groups for that sociodemographic characteristic in all of the 22 countries; Country-specific effects are available in the Supplementary Material, Tables 1b–22b and forest plots are available in Figs. S1–S22.

Somewhat weaker effects were found for the relationship with one’s father (RR = 1.07; 95% CI 1.02,1 .12), self-reported excellent health during upbringing compared to those reporting good health (RR = 1.07; 95% CI 1.03,1. 13), experiences of abuse during childhood (RR = 1.08; 95% CI 1.01, 1.15), and growing up as an outsider (RR = 1.10; 95% CI 1.00, 1.21). A very good or somewhat good relationship with one’s father in childhood–despite not being significant in the country-specific analyses, as indicated by global *p* value = 0.168–was found to positively contribute to adult RR/L. However, substantial heterogeneity (I^2^ = 36.6) indicates that the effects could be positive (e.g., in the United Kingdom and Brazil) or negative (e.g., in Hong Kong; Fig. S2 in the Supplementary Material). Similarly, perceiving one’s health as excellent while growing up was associated with an increased adult RR/L, compared to the perception of a mere good health. Country-specific effects were either null or positive (the strongest in Israel, Japan, and Hong Kong; Fig. S11 in the Supplementary Material), implying moderate cross-national variability as indicated by I^2^ = 66.9.

Both negative childhood experiences–abuse and being an outsider – were associated with an increased likelihood of RR/L, with substantially higher cross-national heterogeneity observed for the former. This former experience was associated with increased adult RR/L in Hong Kong, Australia, Japan, Sweden, and India (in other countries the effects were null). The effects of being an outsider were less unidirectional, as its effect on increased adult RR/L were found in Japan, the United Kingdom, and Spain, while decreased effects were found in the United States and the Philippines.

Other childhood predictors showed little evidence of association with adult reading and listening to sacred texts when estimates were aggregated across countries, as indicated by respective 95% confidence intervals including 1. These include the relationship with one’s mother, parental marital status, and perceived family financial status while growing up. While mother relationship quality was the only predictor for which there was little evidence of an association with RR/L in any of the countries (as a cautionary note, the effect of mother relationship quality was affected by issues of multicollinearity in the estimates within some countries, leading to larger uncertainty in the meta-analytic estimates and imprecise estimates of heterogeneity), perceived family financial status and parental marital status played a role in specific countries. For example, very difficult financial circumstances in childhood were associated with an increased likelihood of reading and listening to sacred texts in adulthood in Mexico, Argentina, and Kenya, but with a decreased likelihood in Germany, Japan, and India (Fig. S8 in the Supplementary Material). Coming from a single or never-married parent family, compared to a family with married parents, was associated with an increased probability of reading and listening to sacred texts in adulthood in Poland, Japan, and the United States, but with a decreased probability in Tanzania and Kenya (Fig. S4 in the Supplementary Material).

Regarding demographic predictors, we found that being female (compared to being male) was associated with an increased likelihood of RR/L (RR = 1.09; 95% CI 1.00, 1.18). Additionally, self-identification as a gender other than male or female was associated with a substantially lower probability of RR/L in adulthood compared to individuals identifying as males (RR = 0.02; 95% CI 0.00, 0.25). However, there was again a considerable heterogeneity in the direction of this effect across countries (as indicated by the estimated proportions with risk ratios above 1.1 or below 0.9, and forest plots presented in Fig. S20 in the Supplementary Material). For example, being female (compared to being male) was associated with an increased probability of reading and listening to sacred texts in Poland and a decreased probability in the United Kingdom. In Hong Kong, Germany, Sweden, Australia, Poland, Israel, Argentina, and the United States, the ‘other gender’ classification implied a lower probability of reading and listening to sacred texts, with decisively stronger effects in the first six of these countries. Conversely, in Indonesia, the Philippines, Kenya, Nigeria, and South Africa, the ‘other gender’ classification was associated with a higher likelihood of RR/L, with these negative effects much stronger than the positive effects. Similarly, being aged 50–79 was associated with an increased likelihood of RR/L compared to the youngest individuals, aged 18–24. These effects were relatively pronounced and consistently homogenous across nations, as indicated by I^2^ statistic (Table 2) and forest plots presented in supplementary Figs. S21–S27. No global effects were found for immigrations status.

Various patterns of associations between race/ethnicity and adult reading and listening to sacred texts were observed. In most countries, the association was more negligible; however, notable exceptions existed. For example, in Argentina, Brazil, Mexico, the United Kingdom, and the United States, belonging to a minority group was associated with more frequent reading and listening to sacred texts in adulthood, whereas in India self-identifying with a minority group was associated with less frequent RR/L (the Supplementary Material, Tables 1b–22b).

**Table 3:** Sensitivity of meta-analyzed childhood predictors of reading sacred texts in adulthood to unmeasured confounding.

Variable	Category	E value	E value for 95% CI limit
Relationship with mother	(Ref: Very bad/somewhat bad)		
	Very/somewhat good	1.11	1.00
Relationship with father	(Ref: Very bad/somewhat bad)		
	Very/somewhat good	1.33	1.15
Parent marital status	(Ref: Parents married)		
	Divorced	1.18	1.00
	Single, never married	1.44	1.00
	One or both had died	1.14	1.00
Subjective financial status of family growing up	(Ref: Got by)		
	Lived comfortably	1.04	1.00
	Found it difficult	1.05	1.00
	Found it very difficult	1.08	1.00
Abuse	(Ref: No)		
	Yes	1.37	1.12
Outsider growing up	(Ref: No)		
	Yes	1.44	1.02
Self-rated health growing up	(Ref: Good)		
	Excellent	1.36	1.19
	Very good	1.29	1.00
	Fair	1.29	1.00
	Poor	1.50	1.00
Immigration status	(Ref: Born in this country)		
	Born in another country	1.30	1.00
Age 12 religious service attendance	(Ref: Never)		
	At least 1/week	4.56	3.02
	1–3/month	3.52	2.33
	Less than 1/month	1.87	1.41
Year of birth	(Ref: 1998–2005; age 18–24)		
	1993–1998; age 25–29	1.20	1.00
	1983–1993; age 30–39	1.26	1.00
	1973–1983; age 40–49	1.38	1.00
	1963–1973; age 50–59	1.59	1.21
	1953–1963; age 60–69	1.61	1.14
	1943–1953; age 70–79	1.77	1.29
	1943 or earlier; age 80+	2.07	1.00
Gender	(Ref: Male)		
	Female	1.40	1.07
	Other	109.16	7.35

Religious affiliation also played a role in the likelihood of reading these texts. In countries where there was evidence of an association (*p*s < 0.05, the Supplementary Material, Tables 1b–22b), individuals reporting a religious affiliation tended to read and listen to sacred texts more frequently. These were observed in Australia, Germany, Hong Kong, Sweden, the United Kingdom, and the United States. Additionally, being a Muslim was associated with a higher probability of RR/L compared to being a Christian in Indonesia, the Philippines and Nigeria, and compared to being a Christian, Hindu, or adherent of other religions in India.

### Robustness analysis

Among the reported associations examined through the meta-analysis, the link between child religious service attendance and adult reading of sacred texts was particularly robust to unmeasured confounding, as indicated by E-values reported in Table 3. For example, considering the most frequent religious service attendance (at least once per week), an unmeasured confounder associated with both childhood religious service attendance and adult RR/L by risk ratios of 4.56 each (above and beyond the covariates already adjusted for) could explain away the association, but weaker joint confounder associations could not. Furthermore, for the confidence interval to include the null value, an unmeasured confounder associated with both childhood religious service attendance and adult religious reading and listening by risk ratios of 3.02 each could explain away the association, but weaker joint confounder associations could not. The other associations were somewhat less robust, especially with regard to the 95% CI and some combination of statistical uncertainty and unmeasured confounding might be sufficient to explain these away.

The meta-analysis conducted with population weights, giving considerable weight to India with its large population, revealed that the association between the relationship with one’s father and RR/L in adulthood was attenuated compared to the random-effects meta-analysis. However, the associations involving subjective health, negative childhood experiences, religious service attendance during childhood, and cohort effects remained robustly linked to adult reading and listening to sacred texts, as detailed in Table S1 in the Supplementary Material. Despite minor variations, the overall pattern of results was generally consistent with those observed in the primary – random-effects meta-analyses, underscoring the robustness of our findings.

## Discussion

This study examined the early-life predictors of RR/L in adulthood across diverse cultural and religious landscapes. We found that upbringing circumstances play a crucial role in developing religiosity, as reflected in reading sacred texts and listening to them. Specifically, childhood religious service attendance was identified as the strongest predictor of adult reading of and listening to sacred texts. This aligns with prior evidence that childhood religiosity, including parental behaviors in this respect, is a well-established predictor of adult religiosity and faith transmission^28–32,34,35,45–47^. However, our results suggest that other factors may also be important. Notably, we found some evidence that a positive relationship with one’s father is important. This finding on the role of parenting for child’s future life outcomes in the realm of religiosity aligns with ecological systems theory^18^ and prior empirical studies on the effects of parental warmth on flourishing in later life^48,49^ and adult religiosity^34,35^. However, the lack of association between the quality of mother-child relationship and adult religiosity, which we found, is intriguing (though this should be interpreted cautiously, as it may result from recall bias-induced multicollinearity, an issue we address in the Limitations section). Despite these methodological issues, prior evidence confirms differential parenting styles in general – mothers are predominantly more authoritative and fathers are mostly more authoritarian^50^ – and the differential influence of fathers and mothers on religious transmission in particular^34,35,45,47^. Our findings on the possibly stronger role of father relationship quality in religiosity, reflected in listening to and reading of sacred texts in adulthood, add new insights. Sterns and McKinney^34,35^ have already reported that while perceived parental warmth facilitated the relationship between perceived parental religiosity and the religiosity of emerging adult women, only paternal warmth facilitated this relationship for men. These findings imply that not only the gender of parents but also of offsprings may play a role in the faith transmission process, an interaction that was not accounted for in our research.

Our findings extend the literature on the effects of adverse childhood experiences and traumas on adult life outcomes, for example^37,51,52^. We found that childhood abuse is associated with increased religiosity, as evidenced by an increased likelihood of reading of and listening to sacred texts in adulthood. This finding contrasts with previous reports on spiritual and religious struggles, worsened God image, and a general decline in religiosity experienced by survivors of childhood traumas^36,37^. We may hypothesize, however, that a possible positive religious coping mechanism could have been in place to overcome such traumas, as indicated by prior research^25,53^. Specifically, as reported by Krause and Pargament^5^for people who experienced stressful life events, reading the Bible positively moderates the relationship between experienced stress and hope. They are also more inclined to rely on positive religious coping mechanisms, through which they reinterpret an adverse event or circumstance as part of a divine plan and an opportunity for spiritual growth and strengthening of one’s faith^5^. Moreover, our study finds that these mechanisms are likely to occur not only for Christians but also for adherents of other religions, though some heterogeneity of effects was observed.

In contrast to previous theoretical arguments and empirical findings based on relative deprivation theory^26,27^which suggests that religiosity acts as a compensatory mechanism for individuals facing economic deprivation, offering future rewards, social connectedness in place of material resources, and meaningful explanations for the lack of material affluence, we found no link between childhood subjective financial status and adult propensity to read and listen to sacred literature. Similar effect of no association between low socioeconomic status and religious service participation among adolescents was found by Sarbu et al.^28^. In our case, it may be that the timespan for most participants was too extended to observe such an effect – an issue that could be addressed in future research by introducing an interaction term with the age cohort variable.

Our study, which demonstrated a higher level of religious reading among older cohorts, is in line with previously documented findings indicating a more pronounced religiosity in older age groups^39^. Our observation that women are more likely to engage in the study of holy texts than men resonates with previous research indicating a stronger religious inclination among women^41,42^. However, our study also noted some cross-national variation in the association between gender and studying sacred texts, lending support to the idea that gender-specific religious preferences can depend on the aspects of religiousness examined, religious affiliation, cultural context, and individual personality traits, as indicated by other researchers^42,54,55^.

In summary, our research highlights the complex interplay of childhood experiences on the engagement with sacred texts in adulthood, with the most pronounced effects of childhood religious socialization observed. While conventional factors, such as parental religiosity, play a significant role in predicting RR/L in adulthood, our study draws attention to the subtle impact of positive paternal relationships on shaping religious practices. The observed cross-national diversity in the predictors of adult RR/L underscores the necessity for future research to adopt culturally responsive methodologies. This will further deepen our comprehension of the lasting influence of childhood experiences on religious practices in adulthood, whilst taking into account the various cultural and religious nuances.

The study has several limitations. First, it is based on self-reported retrospective data, which could be influenced by recall bias. This bias occurs when respondents inaccurately recall or omit details about past events, which could potentially bias the results. However, for recall bias to completely explain away the observed associations would require that the effect of adult religious reading on biasing retrospective assessments of the childhood predictors would essentially have to be at least as strong as the observed associations themselves^56^and a few of these were quite substantial. Second, while our findings are derived from a retrospective cross-sectional dataset, we must exercise caution in interpreting the results causally due to the potential for unmeasured confounding factors. However, the large sample size of 202,898 individuals from 22 diverse countries bolsters the generalizability of our findings. Additionally, the E-value sensitivity analysis aids in evaluating the risk of unmeasured confounding, suggesting that at least some results are moderately robust to such confounding. Finally, our question on engagement in studying holy texts merged aspects of reading and listening to religious literature into a single query. This prevents us from distinguishing between these two forms of engagement. Moreover, this question captures frequency but not duration or content, as well as the purpose of engagement (e.g., for comfort or to learn more about divine characteristics). Prior research has shown that the frequency of prayer affects well-being differently depending on the purpose of the prayer^57^suggesting a similar diverse effect could be expected for religious reading.

Despite these limitations, our findings enrich our understanding of childhood factors that may shape religiosity – a well-known predictor of health and well-being^12,58–69^. This understanding is crucial as it not only contributes to the field of religious studies but also may have implications for health and well-being research. Additionally, although respondents may have found it challenging to distinguish between the two modes of engagement with sacred literature examined in this study, our exploration of both – reading, a traditional form of religious engagement, and listening, a novel and increasingly popular method – provides new insights. The listening aspect, in particular, reflects the growing availability and use of audio options in recent years, offering a unique perspective on religious engagement.

## Materials and methods

The description of the methods below has been adapted from VanderWeele et al.^70^. Further methodological detail is available elsewhere^71–77^.

### Data

Wave 1 data from the Global Flourishing Study (GFS) were used. A total of 202,898 participants from 22 geographically and culturally diverse countries [Argentina, Australia, Brazil, Egypt, Germany, Hong Kong (Special Administrative Region of China), India, Indonesia, Israel, Japan, Kenya, Mexico, Nigeria, the Philippines, Poland, South Africa, Spain, Sweden, Tanzania, Türkiye, the United Kingdom, and the United States] provided information. The GSF is planned as a five-year longitudinal study investigating the predictors of well-being, including life satisfaction and happiness, meaning and purpose in life, social connectedness, financial security, and character and virtue^78^. Additionally, it collects data on religiousness and spirituality, personality, as well as economic, political, and health circumstances. Wave 1 data were collected in 2022–2023.

The GFS was designed to produce nationally representative samples of the adult population (aged 18 and older) across 22 countries. Eligibility required access to a phone or the internet, which was necessary to support follow-up data collection^75,76^. In countries where participants were recruited in person, this requirement led to less than 2% loss of coverage. Three main sampling strategies were used: probability-based sampling (Egypt, India, Indonesia, Israel, Kenya, Nigeria, Philippines, South Africa, Tanzania, Turkey, and the United States), non-probability-based sampling using existing web panels (Hong Kong SAR, Japan, and Sweden), and a combination of the two approaches (Argentina, Australia, Brazil, Germany, Mexico, Poland, Spain, and the United Kingdom). In countries using a mixed approach, non-probability samples were added to improve demographic coverage by sex, age, and region. Recruitment modes varied across countries. Some countries, like the United States, used only web-based recruitment, while others, such as Israel and Kenya, relied solely on face-to-face recruitment. Several countries used a mix of methods, including Argentina and Mexico. In Nigeria, an additional subsample was collected to represent adults without access to mobile devices or the internet. A similar attempt in India was initiated but not completed due to the very small size of this subgroup^75,76^.

More information about the study can be found elsewhere^71–77^. Access to data and codebooks are available at https://www.cos.io/gfs. The study was exempted from review by the Institutional Review Board of Baylor University. All respondents provided informed consent.

### Measures

#### Outcome variable

 Religious reading was measured using a question adapted from the Brief Multidimensional Measure of Religiousness/Spirituality (BMMRS)^4^: ‘How often do you read or listen to sacred texts or other religious literature?’ The measure is free to use in scientific research^79^. The response options were: More than once a day, about once a day, sometimes, never. Based on cognitive interview results responses were slightly modified^71^. For analyses, religious reading was dichotomized into (0) sometimes/never and (1) more than once a day/about once a day.

#### Childhood predictors

 The following childhood predictors related to childhood experiences, personal attributes, and familial or social circumstances (around the age of 12 years of a respondent) were tested: quality of mother/father relationship (very good, somewhat good, somewhat bad, or very bad, a ‘does not apply’ category was used as a dichotomous variable for those without a mother or father), parental marital status (married, divorced, never married, one/both parents deceased), perception of family’s household income (lived comfortably, got by, found it difficult, and found it very difficult), experiences of abuse (yes, no), feeling like an outsider in one’s family (yes, no), health (excellent, very good, good, fair, or poor), religious service attendance (ranging from at least once a week to never), and religious tradition/affiliation [Christianity, Islam, Hinduism, Buddhism, Judaism, Sikhism, Baha’i, Jainism, Shinto, Taoism, Confucianism, Primal/Animist/Folk religion, Spiritism, African-Derived, some other religion, or no religion/atheist/agnostic; response categories varying by country^80^. We initially planned to include variables reflecting parental love as early-life determinants; however, these variables were found to be substantially correlated with the quality of mother/father relationship variables, leading to convergence issues. Consequently, we modified our list of descriptors by excluding the parental love variables. This information was added to the preregistration.

Basic demographic characteristics included year of birth, gender (male, female, other), racial/ethnic identity (with country-specific response categories), and immigration status (born in the country of the survey or not).

### Analysis

To examine the associations between childhood predictors and reading sacred texts, a weighted Poisson regression with adjusted standard errors was conducted separately within each country (presented in the Supplementary Material). A random effects meta-analysis was used to aggregate country-specific coefficients across countries^81,82^ (coefficients of ethnicity/race and religious affiliation were not included due to country-specific variations in response categories). Forest plots of these estimates are presented in the Supplementary Material. Proportions of effects with risk ratios exceeding 1.1 or below 0.9 were estimated, and I^2^ statistics were used to evaluate variation within predictor categories across countries^83^.

All missing were multiply imputed using chained equations, generating five datasets^84–87^. To account for variations in the assessment of race/ethnicity and religious affiliation across countries, the imputation process was conducted separately within each country. The *p* values of global tests of association for each childhood predictor with the religious text reading outcome within country and pooled *p* values^88^ are reported. Bonferroni correction for multiple testing was applied^89,90^. All analyses were conducted using weights reflecting the complex sample designed applied in the GFS^73^.

The robustness of our results was examined in two ways. First, potential unmeasured confounding was assessed using E-values, computed for the estimate and confidence interval of each regression coefficient. E-values indicate the minimum strength of an association an unmeasured confounder must have with both the predictor and the outcome to nullify an observed association^91^. Second, a population-weighted random-effects meta-analysis was conducted. All analyses were pre-registered with COS prior to data access (https://osf.io/637hg) and analysis code is available online^74^. All meta-analyses were conducted in R^92^ using the *metafor* package^93^. Other calculations were conducted using Stata.

## Electronic supplementary material

Below is the link to the electronic supplementary material.

Supplementary Material 1

## Data Availability

The data are publicly available through the Center for Open Science (https://www.cos.io/gfs), Wave 1 non-sensitive Global data https://osf.io/sm4cd/. The research questions, variables, and analytic plan for the current study were preregistered with the Center for Open Science prior to accessing data (https://osf.io/637hg); all code to reproduce analyses are openly available in an online repository: https://osf.io/vbype/files/osfstorage? view_only=0372838c315d46a995c122f9c637ae5d.
