# Supplementary material for: Childhood predictors of religious reading: a cross-national analysis in the Global Flourishing Study
Source: Sci Rep. 2025 Jul 10;15:24899. doi: 10.1038/s41598-025-10142-7 (PMC12246113; doi:10.1038/s41598-025-10142-7)

Childhood Predictors of Religious Reading: A Cross-National Analysis in the Global Flourishing Study

Dorota Weziak-Bialowolska, Richard G. Cowden, Piotr Bialowolski, Matt Bradshaw,
R. Noah Padgett, Byron R. Johnson, Tyler J. VanderWeele

Supplementary Material

- Table S1a – S22c: Supplementary tables for country-specific analyses.
- Table S23: Supplementary table for population weighted meta-analysis.
- Table S24: Supplementary table for population weighted meta-analysis of E-values.
- Figure S1– S27: Supplementary figures for estimates of childhood predictor categories.

Table S1a: Nationally Representative Descriptive Statistics of the Observed Sample (Argentina)

Variable	Proportion	Frequency
Relationship with Mother		
Very Good	0.66	4463
Somewhat Good	0.21	1436
Somewhat Bad	0.04	299
Very Bad	0.03	216
Not Applicable	0.04	273
Missing	0.01	36
Relationship with Father		
Very Good	0.54	3612
Somewhat Good	0.23	1537
Somewhat Bad	0.07	440
Very Bad	0.06	401
Not Applicable	0.10	694
Missing	0.01	39
Parent Marital Status		
Married	0.61	4110
Divorced	0.09	637
Never Married	0.20	1368
One or Both Had Died	0.03	199
Missing	0.06	410
Childhood Income		
Lived Comfortably	0.30	2042

Got By	0.34	2305
Found it Difficult	0.27	1789
Found it Very Difficult	0.08	569
Missing	0.00	19
Childhood Abuse		
Yes	0.19	1302
No	0.78	5271
Missing	0.02	151
Outsider		
Yes	0.17	1165
No	0.81	5458
Not Applicable	0.01	68
Missing	0.00	33
Childhood Health		
Excellent	0.36	2402
Very Good	0.27	1819
Good	0.27	1830
Fair	0.08	505
Poor	0.02	156
Missing	0.00	12
Immigration Status		
Born in This Country	0.94	6346
Born in Another Country	0.05	348
Missing	0.00	29
Childhood Service Attendance		
At Least 1/Week	0.39	2601
1-3/Month	0.18	1204
<1/Month	0.16	1059
Never	0.27	1808
Missing	0.01	53
Gender		
Male	0.47	3143
Female	0.53	3542
Other	0.00	21
Missing	0.00	18
Year of Birth		
1998-2005; Age 18-24	0.16	1108
1993-1998; Age 25-29	0.11	719
1983-1993; Age 30-39	0.21	1432
1973-1983; Age 40-49	0.19	1254
1963-1973; Age 50-59	0.15	1014
1953-1963; Age 60-69	0.11	730
1943-1953; Age 70-79	0.05	356
1943 or Earlier; 80 or Older	0.02	112
Missing	.	.
Childhood Religion		
Christianity	0.86	5805
Islam	0.00	11
Hinduism	0.00	2
Buddhism	0.00	3
Judaism	0.01	51

Sikhism	0.00	5
Baha'i	.	.
Jainism	.	.
Shinto	.	.
Taoism	0.00	1
Confucianism	.	.
Primal, Animist, or Folk Religion	0.00	17
Spiritism	.	.
African-Derived	.	.
Chinese	.	.
Some Other Religion	0.00	10
No Religion/Atheist/Agnostic	0.10	697
Missing	0.02	122
Race/Ethnicity		
Asian	0.01	43
Black	0.01	95
Indigenous	0.02	129
Mestizo(a)	0.27	1801
Mullato(a)	0.01	75
White	0.51	3406
Other	0.02	104
Missing	0.16	1070

Table S1b: Variations Across Childhood Predictors (Argentina)

Variable	IRR	SE	Prob	LCI	UCI	Global p-value
Relationship with Mother (Ref: Very/Somewhat Bad)						
Very/Somewhat Good	0.92	0.12	0.52	0.70	1.20	0.52
Relationship with Father (Ref: Very/Somewhat Bad)						
Very/Somewhat Good	1.22	0.14	0.08	0.98	1.52	0.08
Parent Marital Status (Ref: Married)						
Divorced	0.94	0.13	0.66	0.73	1.23	0.93
Never Married	0.94	0.10	0.56	0.77	1.16	.
One or Both Had Died	0.93	0.20	0.73	0.60	1.43	.
Childhood Income (Ref: Got By)						
Lived Comfortably	1.05	0.09	0.58	0.88	1.26	0.19
Found it Difficult	1.11	0.11	0.28	0.92	1.33	.
Found it Very Difficult	1.33	0.18	0.03	1.03	1.73	.
Childhood Abuse (Ref: No)						
Yes	1.12	0.10	0.19	0.94	1.34	0.19
Outsider (Ref: No)						
Yes	1.01	0.11	0.91	0.81	1.26	0.91
Childhood Health (Ref: Good)						
Excellent	1.04	0.10	0.70	0.86	1.25	0.16
Very Good	1.02	0.10	0.83	0.84	1.24	.
Fair	1.33	0.17	0.02	1.04	1.70	.
Poor	1.27	0.30	0.30	0.80	2.02	.
Immigration Status (Ref: Born in This Country)						

Born in Another Country	1.08	0.16	0.59	0.81	1.44	0.59
Childhood Service Attendance (Ref: Never)						
At Least 1/Week	2.01	0.22	0.00	1.63	2.49	0.00
1-3/Month	1.53	0.20	0.00	1.18	1.97	.
<1/Month	1.34	0.19	0.04	1.01	1.76	.
Gender (Ref: Male)						
Female	1.13	0.08	0.10	0.98	1.31	0.00
Other	0.05	0.05	0.00	0.01	0.40	.
Year of Birth (Ref: 1998-2005)						
1993-1998; Age 25-29	1.44	0.22	0.02	1.07	1.93	0.02
1983-1993; Age 30-39	1.33	0.19	0.04	1.01	1.75	.
1973-1983; Age 40-49	1.25	0.18	0.13	0.94	1.65	.
1963-1973; Age 50-59	1.60	0.23	0.00	1.21	2.13	.
1953-1963; Age 60-69	1.41	0.23	0.03	1.03	1.93	.
1943-1953; Age 70-79	1.85	0.34	0.00	1.29	2.67	.
1943 or Earlier; Age 80 or Older	1.54	0.43	0.12	0.89	2.65	.
Mother Absence/Presence (Ref: Present)						
Absent	0.98	0.15	0.91	0.73	1.33	0.91
Father Absence/Presence (Ref: Present)						
Absent	1.25	0.16	0.08	0.97	1.60	0.08
Childhood Religion (Ref: No Religion/Atheist/Agnostic)						
Christianity	1.11	0.17	0.48	0.83	1.49	0.52
Some Other Religion	1.41	0.43	0.26	0.77	2.56	.
Race/Ethnicity (Ref: Ethnic Plurality)						
Ethnic Minority	1.18	0.10	0.05	1.00	1.38	0.05

Table S1c: E-Values and E-Value Limits (Argentina)

Variable	E-Value	E-Value Limit
Relationship with Mother (Ref: Very/Somewhat Bad)		
Very/Somewhat Good	1.44	1.00
Relationship with Father (Ref: Very/Somewhat Bad)		
Very/Somewhat Good	1.80	1.00
Parent Marital Status (Ref: Married)		
Divorced	1.34	1.00
Never Married	1.34	1.00
One or Both Had Died	1.40	1.00
Childhood Income (Ref: Got By)		
Lived Comfortably	1.30	1.00
Found it Difficult	1.49	1.00
Found it Very Difficult	2.10	1.13
Childhood Abuse (Ref: No)		
Yes	1.53	1.00
Outsider (Ref: No)		
Yes	1.13	1.00
Childhood Health (Ref: Good)		
Excellent	1.25	1.00
Very Good	1.18	1.00
Fair	2.09	1.17
Poor	1.94	1.00

Immigration Status (Ref: Born in This Country)		
Born in Another Country	1.41	1.00
Childhood Service Attendance (Ref: Never)		
At Least 1/Week	3.78	1.87
1-3/Month	2.58	1.39
<1/Month	2.10	1.09
Gender (Ref: Male)		
Female	1.55	1.00
Other	52.46	2.55
Year of Birth (Ref: 1998-2005)		
1993-1998; Age 25-29	2.36	1.22
1983-1993; Age 30-39	2.08	1.07
1973-1983; Age 40-49	1.87	1.00
1963-1973; Age 50-59	2.77	1.43
1953-1963; Age 60-69	2.29	1.13
1943-1953; Age 70-79	3.39	1.53
1943 or Earlier; Age 80 or Older	2.61	1.00
Mother Absence/Presence (Ref: Present)		
Absent	1.16	1.00
Father Absence/Presence (Ref: Present)		
Absent	1.87	1.00
Childhood Religion (Ref: No Religion/Atheist/Agnostic)		
Christianity	1.50	1.00
Some Other Religion	2.28	1.00
Race/Ethnicity (Ref: Ethnic Plurality)		
Ethnic Minority	1.68	1.02

Table S2a: Nationally Representative Descriptive Statistics of the Observed Sample (Australia)

Variable	Proportion	Frequency
Relationship with Mother		
Very Good	0.66	2554
Somewhat Good	0.24	925
Somewhat Bad	0.06	218
Very Bad	0.03	107
Not Applicable	0.01	32
Missing	0.00	7
Relationship with Father		
Very Good	0.53	2032
Somewhat Good	0.30	1144
Somewhat Bad	0.08	315
Very Bad	0.05	196
Not Applicable	0.04	148
Missing	0.00	9
Parent Marital Status		
Married	0.79	3048
Divorced	0.12	462
Never Married	0.05	187
One or Both Had Died	0.02	96

Missing	0.01	52
Childhood Income		
Lived Comfortably	0.46	1756
Got By	0.39	1496
Found it Difficult	0.11	422
Found it Very Difficult	0.04	154
Missing	0.00	16
Childhood Abuse		
Yes	0.26	995
No	0.73	2790
Missing	0.02	59
Outsider		
Yes	0.20	756
No	0.80	3062
Not Applicable	0.00	6
Missing	0.00	19
Childhood Health		
Excellent	0.45	1736
Very Good	0.28	1087
Good	0.16	603
Fair	0.08	308
Poor	0.03	106
Missing	0.00	4
Immigration Status		
Born in This Country	0.77	2953
Born in Another Country	0.23	885
Missing	0.00	6
Childhood Service Attendance		
At Least 1/Week	0.35	1362
1-3/Month	0.13	486
<1/Month	0.16	600
Never	0.34	1307
Missing	0.02	90
Gender		
Male	0.48	1861
Female	0.50	1941
Other	0.01	36
Missing	0.00	6
Year of Birth		
1998-2005; Age 18-24	0.09	345
1993-1998; Age 25-29	0.07	282
1983-1993; Age 30-39	0.17	641
1973-1983; Age 40-49	0.16	618
1963-1973; Age 50-59	0.18	691
1953-1963; Age 60-69	0.15	589
1943-1953; Age 70-79	0.13	498
1943 or Earlier; 80 or Older	0.05	178
Missing	0.00	2
Childhood Religion		

Christianity	0.70	2678
Islam	0.01	48
Hinduism	0.01	39
Buddhism	0.00	16
Judaism	0.01	29
Sikhism	0.00	6
Baha'i	0.00	5
Jainism	.	.
Shinto	.	.
Taoism	0.00	1
Confucianism	.	.
Primal, Animist, or Folk Religion	0.00	4
Spiritism	.	.
African-Derived	.	.
Chinese	.	.
Some Other Religion	0.00	8
No Religion/Atheist/Agnostic	0.26	990
Missing	0.01	21
Race/Ethnicity		
Aboriginal	0.01	53
Australian	0.51	1946
Australian /British/European	0.27	1047
Chinese	0.02	75
Indian	0.02	58
Japanese	0.00	1
Malay	0.00	11
Sinhalese	0.00	1
Spanish	0.00	2
Sri Lankan Moor	0.00	1
Sri Lankan Tamil	0.00	7
Vietnamese	0.00	7
Taiwanese/Holo	.	.
Russian	0.00	7
Samoan	0.00	4
New Zealander	0.02	91
Other European	0.09	357
Other	0.04	163
Missing	0.00	14

Table S2b: Variations Across Childhood Predictors (Australia)

Variable	IRR	SE	Prob	LCI	UCI	Global p-value
Relationship with Mother (Ref: Very/Somewhat Bad)						
Very/Somewhat Good	1.32	0.33	0.26	0.81	2.16	0.26
Relationship with Father (Ref: Very/Somewhat Bad)						

Very/Somewhat Good	1.44	0.34	0.12	0.91	2.28	0.12
Parent Marital Status (Ref: Married)						
Divorced	0.56	0.17	0.05	0.31	1.01	0.10
Never Married	1.23	0.54	0.64	0.52	2.91	.
One or Both Had Died	1.42	0.42	0.24	0.79	2.56	.
Childhood Income (Ref: Got By)						
Lived Comfortably	0.96	0.12	0.73	0.76	1.22	0.68
Found it Difficult	0.77	0.17	0.23	0.51	1.18	.
Found it Very Difficult	1.03	0.34	0.92	0.54	1.97	.
Childhood Abuse (Ref: No)						
Yes	1.40	0.18	0.01	1.08	1.80	0.01
Outsider (Ref: No)						
Yes	1.16	0.20	0.37	0.83	1.62	0.37
Childhood Health (Ref: Good)						
Excellent	1.18	0.21	0.35	0.83	1.67	0.86
Very Good	1.18	0.22	0.37	0.82	1.70	.
Fair	1.32	0.36	0.31	0.77	2.26	.
Poor	1.11	0.44	0.80	0.51	2.40	.
Immigration Status (Ref: Born in This Country)						
Born in Another Country	1.32	0.19	0.05	1.00	1.74	0.05
Childhood Service Attendance (Ref: Never)						
At Least 1/Week	3.77	0.80	0.00	2.49	5.72	0.00
1-3/Month	2.35	0.62	0.00	1.40	3.92	.
<1/Month	1.54	0.41	0.10	0.92	2.58	.
Gender (Ref: Male)						
Female	1.27	0.14	0.03	1.02	1.57	0.00
Other	0.00	0.00	0.00	0.00	0.00	.
Year of Birth (Ref: 1998-2005)						
1993-1998; Age 25-29	1.65	0.61	0.18	0.80	3.42	0.00
1983-1993; Age 30-39	0.84	0.30	0.63	0.42	1.69	.
1973-1983; Age 40-49	1.63	0.53	0.13	0.86	3.07	.
1963-1973; Age 50-59	1.20	0.40	0.58	0.62	2.32	.
1953-1963; Age 60-69	1.10	0.36	0.76	0.58	2.10	.
1943-1953; Age 70-79	1.35	0.45	0.37	0.70	2.60	.
1943 or Earlier; Age 80 or Older	2.65	0.89	0.00	1.37	5.13	.
Mother Absence/Presence (Ref: Present)						
Absent	0.41	0.19	0.05	0.17	1.01	0.05
Father Absence/Presence (Ref: Present)						
Absent	1.25	0.38	0.46	0.69	2.27	0.46
Childhood Religion (Ref: No Religion/Atheist/Agnostic)						
Christianity	2.24	0.61	0.00	1.31	3.80	0.00
Some Other Religion	4.14	1.29	0.00	2.25	7.64	.
Race/Ethnicity (Ref: Ethnic Plurality)						
Ethnic Minority	0.97	0.13	0.81	0.75	1.25	0.81

Table S2c: E-Values and E-Value Limits (Australia)

Variable	E-Value	E-Value Limit
Relationship with Mother (Ref: Very/Somewhat Bad)		
Very/Somewhat Good	2.46	1.00
Relationship with Father (Ref: Very/Somewhat Bad)		
Very/Somewhat Good	2.92	1.00
Parent Marital Status (Ref: Married)		
Divorced	4.36	1.00
Never Married	2.09	1.00
One or Both Had Died	2.84	1.00
Childhood Income (Ref: Got By)		
Lived Comfortably	1.33	1.00
Found it Difficult	2.35	1.00
Found it Very Difficult	1.28	1.00
Childhood Abuse (Ref: No)		
Yes	2.74	1.25
Outsider (Ref: No)		
Yes	1.84	1.00
Childhood Health (Ref: Good)		
Excellent	1.91	1.00
Very Good	1.91	1.00
Fair	2.45	1.00
Poor	1.61	1.00
Immigration Status (Ref: Born in This Country)		
Born in Another Country	2.44	1.00
Childhood Service Attendance (Ref: Never)		
At Least 1/Week	15.04	2.53
1-3/Month	6.93	1.65
<1/Month	3.32	1.00
Gender (Ref: Male)		
Female	2.23	1.10
Other	2.78e+08	591.88
Year of Birth (Ref: 1998-2005)		
1993-1998; Age 25-29	3.76	1.00
1983-1993; Age 30-39	1.93	1.00
1973-1983; Age 40-49	3.66	1.00
1963-1973; Age 50-59	2.00	1.00
1953-1963; Age 60-69	1.61	1.00
1943-1953; Age 70-79	2.55	1.00
1943 or Earlier; Age 80 or Older	8.48	1.62
Mother Absence/Presence (Ref: Present)		
Absent	7.46	1.00
Father Absence/Presence (Ref: Present)		
Absent	2.18	1.00
Childhood Religion (Ref: No Religion/Atheist/Agnostic)		
Christianity	6.39	1.56
Some Other Religion	17.47	2.36
Race/Ethnicity (Ref: Ethnic Plurality)		
Ethnic Minority	1.28	1.00

Table S3a: Nationally Representative Descriptive Statistics of the Observed Sample (Brazil)

Variable	Proportion	Frequency
Relationship with Mother		
Very Good	0.63	8369
Somewhat Good	0.27	3559
Somewhat Bad	0.04	483
Very Bad	0.02	214
Not Applicable	0.04	507
Missing	0.01	73
Relationship with Father		
Very Good	0.48	6364
Somewhat Good	0.28	3654
Somewhat Bad	0.08	1035
Very Bad	0.06	756
Not Applicable	0.10	1303
Missing	0.01	93
Parent Marital Status		
Married	0.65	8546
Divorced	0.10	1384
Never Married	0.15	1985
One or Both Had Died	0.04	508
Missing	0.06	781
Childhood Income		
Lived Comfortably	0.38	4998
Got By	0.35	4616
Found it Difficult	0.19	2484
Found it Very Difficult	0.08	1027
Missing	0.01	79
Childhood Abuse		
Yes	0.20	2606
No	0.77	10147
Missing	0.03	451
Outsider		
Yes	0.13	1659
No	0.85	11234
Not Applicable	0.02	229
Missing	0.01	82
Childhood Health		
Excellent	0.40	5312
Very Good	0.26	3392
Good	0.22	2873
Fair	0.10	1368
Poor	0.02	228
Missing	0.00	30
Immigration Status		
Born in This Country	0.96	12688

Born in Another Country	0.01	153
Missing	0.03	363
Childhood Service Attendance		
At Least 1/Week	0.48	6306
1-3/Month	0.19	2491
<1/Month	0.20	2629
Never	0.13	1707
Missing	0.01	71
Gender		
Male	0.48	6320
Female	0.52	6820
Other	0.00	35
Missing	0.00	30
Year of Birth		
1998-2005; Age 18-24	0.15	1986
1993-1998; Age 25-29	0.11	1468
1983-1993; Age 30-39	0.22	2908
1973-1983; Age 40-49	0.20	2638
1963-1973; Age 50-59	0.16	2131
1953-1963; Age 60-69	0.11	1435
1943-1953; Age 70-79	0.04	510
1943 or Earlier; 80 or Older	0.01	126
Missing	.	.
Childhood Religion		
Christianity	0.86	11403
Islam	0.00	14
Hinduism	0.00	1
Buddhism	0.00	27
Judaism	0.00	40
Sikhism	.	.
Baha'i	0.00	1
Jainism	0.00	4
Shinto	0.00	4
Taoism	0.00	1
Confucianism	0.00	7
Primal, Animist, or Folk Religion	0.00	17
Spiritism	0.03	336
African-Derived	0.02	262
Chinese	.	.
Some Other Religion	0.01	87
No Religion/Atheist/Agnostic	0.07	908
Missing	0.01	94
Race/Ethnicity		
Branca	0.39	5169
Preta	0.12	1615
Parda	0.39	5125
Amarela	0.02	238
Indigena	0.01	131
Other	0.00	61

Missing	0.07	865

Table S3b: Variations Across Childhood Predictors (Brazil)

Variable	IRR	SE	Prob	LCI	UCI	Global p-value
Relationship with Mother (Ref: Very/Somewhat Bad)						
Very/Somewhat Good	1.06	0.06	0.30	0.95	1.19	0.30
Relationship with Father (Ref: Very/Somewhat Bad)						
Very/Somewhat Good	1.10	0.05	0.02	1.01	1.20	0.02
Parent Marital Status (Ref: Married)						
Divorced	0.98	0.05	0.69	0.89	1.08	0.96
Never Married	1.01	0.05	0.82	0.92	1.11	.
One or Both Had Died	1.01	0.08	0.86	0.87	1.19	.
Childhood Income (Ref: Got By)						
Lived Comfortably	0.98	0.03	0.51	0.91	1.05	0.14
Found it Difficult	1.05	0.04	0.21	0.97	1.14	.
Found it Very Difficult	1.08	0.06	0.14	0.98	1.20	.
Childhood Abuse (Ref: No)						
Yes	1.02	0.04	0.53	0.95	1.10	0.53
Outsider (Ref: No)						
Yes	0.99	0.04	0.87	0.91	1.08	0.87
Childhood Health (Ref: Good)						
Excellent	1.06	0.04	0.11	0.99	1.15	0.01
Very Good	0.96	0.04	0.29	0.88	1.04	.
Fair	0.95	0.05	0.38	0.85	1.06	.
Poor	1.16	0.11	0.13	0.96	1.40	.
Immigration Status (Ref: Born in This Country)						
Born in Another Country	1.11	0.15	0.44	0.85	1.46	0.44
Childhood Service Attendance (Ref: Never)						
At Least 1/Week	1.69	0.10	0.00	1.51	1.90	0.00
1-3/Month	1.39	0.09	0.00	1.23	1.58	.
<1/Month	1.02	0.07	0.81	0.89	1.16	.
Gender (Ref: Male)						
Female	1.12	0.03	0.00	1.06	1.19	0.00
Other	0.82	0.25	0.51	0.45	1.49	.
Year of Birth (Ref: 1998-2005)						
1993-1998; Age 25-29	1.11	0.07	0.08	0.99	1.25	0.00
1983-1993; Age 30-39	1.27	0.06	0.00	1.16	1.40	.
1973-1983; Age 40-49	1.46	0.07	0.00	1.33	1.61	.
1963-1973; Age 50-59	1.63	0.08	0.00	1.47	1.80	.
1953-1963; Age 60-69	1.61	0.10	0.00	1.43	1.83	.
1943-1953; Age 70-79	1.58	0.15	0.00	1.30	1.91	.
1943 or Earlier; Age 80 or Older	1.78	0.29	0.00	1.29	2.45	.
Mother Absence/Presence (Ref: Present)						

Absent	1.06	0.07	0.33	0.94	1.20	0.33
Father Absence/Presence (Ref: Present)						
Absent	1.00	0.05	0.95	0.91	1.10	0.95
Childhood Religion (Ref: No Religion/Atheist/Agnostic)						
Christianity	0.99	0.06	0.92	0.88	1.13	0.38
Some Other Religion	1.07	0.09	0.38	0.91	1.26	.
Race/Ethnicity (Ref: Ethnic Plurality)						
Ethnic Minority	1.19	0.04	0.00	1.12	1.27	0.00

Table S3c: E-Values and E-Value Limits (Brazil)

Variable	E-Value	E-Value Limit
Relationship with Mother (Ref: Very/Somewhat Bad)		
Very/Somewhat Good	1.28	1.00
Relationship with Father (Ref: Very/Somewhat Bad)		
Very/Somewhat Good	1.38	1.09
Parent Marital Status (Ref: Married)		
Divorced	1.14	1.00
Never Married	1.10	1.00
One or Both Had Died	1.12	1.00
Childhood Income (Ref: Got By)		
Lived Comfortably	1.15	1.00
Found it Difficult	1.25	1.00
Found it Very Difficult	1.33	1.00
Childhood Abuse (Ref: No)		
Yes	1.16	1.00
Outsider (Ref: No)		
Yes	1.08	1.00
Childhood Health (Ref: Good)		
Excellent	1.28	1.00
Very Good	1.23	1.00
Fair	1.24	1.00
Poor	1.50	1.00
Immigration Status (Ref: Born in This Country)		
Born in Another Country	1.40	1.00
Childhood Service Attendance (Ref: Never)		
At Least 1/Week	2.41	1.76
1-3/Month	1.93	1.45
<1/Month	1.13	1.00
Gender (Ref: Male)		
Female	1.42	1.20
Other	1.63	1.00
Year of Birth (Ref: 1998-2005)		
1993-1998; Age 25-29	1.40	1.00
1983-1993; Age 30-39	1.71	1.36
1973-1983; Age 40-49	2.05	1.57

1963-1973; Age 50-59	2.31	1.72
1953-1963; Age 60-69	2.29	1.68
1943-1953; Age 70-79	2.23	1.54
1943 or Earlier; Age 80 or Older	2.54	1.53
Mother Absence/Presence (Ref: Present)		
Absent	1.28	1.00
Father Absence/Presence (Ref: Present)		
Absent	1.05	1.00
Childhood Religion (Ref: No Religion/Atheist/Agnostic)		
Christianity	1.08	1.00
Some Other Religion	1.31	1.00
Race/Ethnicity (Ref: Ethnic Plurality)		
Ethnic Minority	1.57	1.31

Table S4a: Nationally Representative Descriptive Statistics of the Observed Sample (Egypt)

Variable	Proportion	Frequency
Relationship with Mother		
Very Good	0.87	4110
Somewhat Good	0.11	505
Somewhat Bad	0.00	21
Very Bad	0.00	10
Not Applicable	0.02	83
Missing	.	.
Relationship with Father		
Very Good	0.79	3713
Somewhat Good	0.14	683
Somewhat Bad	0.01	56
Very Bad	0.01	30
Not Applicable	0.05	233
Missing	0.00	14
Parent Marital Status		
Married	0.86	4049
Divorced	0.03	131
Never Married	0.00	9
One or Both Had Died	0.10	485
Missing	0.01	55
Childhood Income		
Lived Comfortably	0.26	1251
Got By	0.50	2352
Found it Difficult	0.18	857
Found it Very Difficult	0.06	268
Missing	0.00	1
Childhood Abuse		
Yes	0.09	405
No	0.91	4293
Missing	0.01	30
Outsider		

Yes	0.05	260
No	0.94	4456
Not Applicable	0.00	4
Missing	0.00	10
Childhood Health		
Excellent	0.57	2687
Very Good	0.25	1174
Good	0.11	497
Fair	0.06	265
Poor	0.02	106
Missing	0.00	1
Immigration Status		
Born in This Country	1.00	4713
Born in Another Country	0.00	16
Missing	0.00	1
Childhood Service Attendance		
At Least 1/Week	0.49	2307
1-3/Month	0.12	570
<1/Month	0.13	629
Never	0.25	1165
Missing	0.01	57
Gender		
Male	0.51	2394
Female	0.49	2334
Other	.	.
Missing	0.00	0
Year of Birth		
1998-2005; Age 18-24	0.20	960
1993-1998; Age 25-29	0.13	607
1983-1993; Age 30-39	0.25	1204
1973-1983; Age 40-49	0.19	897
1963-1973; Age 50-59	0.13	613
1953-1963; Age 60-69	0.08	387
1943-1953; Age 70-79	0.01	54
1943 or Earlier; 80 or Older	0.00	7
Missing	.	.
Childhood Religion		
Christianity	0.03	123
Islam	0.97	4602
Hinduism	.	.
Buddhism	.	.
Judaism	.	.
Sikhism	.	.
Baha'i	.	.
Jainism	0.00	1
Shinto	.	.
Taoism	0.00	0
Confucianism	.	.
Primal, Animist, or Folk Religion	.	.

Spiritism	.	.
African-Derived	.	.
Chinese	.	.
Some Other Religion	.	.
No Religion/Atheist/Agnostic	.	.
Missing	0.00	3
Race/Ethnicity		
Arab	0.97	4585
Turkish	0.00	9
Greek	0.00	1
Abazas	.	.
Bedouin Arab	0.00	4
Swiss	.	.
Nubian	0.01	27
Other	.	.
Missing	0.02	102

Table S4b: Variations Across Childhood Predictors (Egypt)

Variable	IRR	SE	Prob	LCI	UCI	Global p-value
Relationship with Mother (Ref: Very/Somewhat Bad)						
Very/Somewhat Good	0.92	0.11	0.49	0.72	1.17	0.49
Relationship with Father (Ref: Very/Somewhat Bad)						
Very/Somewhat Good	0.99	0.08	0.91	0.84	1.17	0.91
Parent Marital Status (Ref: Married)						
Divorced	1.00	0.09	0.96	0.85	1.19	0.82
Never Married	1.20	0.24	0.35	0.82	1.77	.
One or Both Had Died	1.01	0.06	0.88	0.90	1.14	.
Childhood Income (Ref: Got By)						
Lived Comfortably	0.94	0.03	0.10	0.88	1.01	0.32
Found it Difficult	0.97	0.03	0.33	0.90	1.04	.
Found it Very Difficult	0.98	0.07	0.75	0.85	1.12	.
Childhood Abuse (Ref: No)						
Yes	0.98	0.05	0.74	0.88	1.09	0.74
Outsider (Ref: No)						
Yes	0.96	0.07	0.56	0.84	1.10	0.56
Childhood Health (Ref: Good)						
Excellent	1.05	0.04	0.26	0.96	1.14	0.19
Very Good	0.98	0.05	0.74	0.88	1.09	.
Fair	0.98	0.08	0.79	0.84	1.15	.
Poor	1.10	0.09	0.24	0.94	1.30	.
Immigration Status (Ref: Born in This Country)						
Born in Another Country	1.29	0.28	0.24	0.84	1.99	0.24

Childhood Service Attendance (Ref: Never)						
At Least 1/Week	1.10	0.05	0.02	1.01	1.20	0.02
1-3/Month	0.99	0.06	0.85	0.88	1.11	.
<1/Month	0.98	0.05	0.66	0.89	1.08	.
Gender (Ref: Male)						
Female	1.17	0.04	0.00	1.10	1.24	0.00
Other	1.00	.	.	.	.	.
Year of Birth (Ref: 1998-2005)						
1993-1998; Age 25-29	0.92	0.06	0.20	0.82	1.04	0.00
1983-1993; Age 30-39	0.94	0.05	0.19	0.85	1.03	.
1973-1983; Age 40-49	1.03	0.05	0.55	0.94	1.13	.
1963-1973; Age 50-59	1.17	0.06	0.01	1.04	1.30	.
1953-1963; Age 60-69	1.33	0.08	0.00	1.18	1.50	.
1943-1953; Age 70-79	1.15	0.15	0.29	0.89	1.49	.
1943 or Earlier; Age 80 or Older	1.15	0.46	0.72	0.53	2.53	.
Mother Absence/Presence (Ref: Present)						
Absent	0.94	0.13	0.65	0.72	1.23	0.65
Father Absence/Presence (Ref: Present)						
Absent	1.01	0.10	0.88	0.84	1.23	0.88
Childhood Religion (Ref: Islam)						
Some Other Religion	1.07	0.10	0.44	0.90	1.28	0.44
Race/Ethnicity (Ref: Ethnic Plurality)						
Ethnic Minority	0.76	0.16	0.19	0.51	1.14	0.19

Table S4c: E-Values and E-Value Limits (Egypt)

Variable	E-Value	E-Value Limit
Relationship with Mother (Ref: Very/Somewhat Bad)		
Very/Somewhat Good	1.30	1.00
Relationship with Father (Ref: Very/Somewhat Bad)		
Very/Somewhat Good	1.09	1.00
Parent Marital Status (Ref: Married)		
Divorced	1.06	1.00
Never Married	1.51	1.00
One or Both Had Died	1.08	1.00
Childhood Income (Ref: Got By)		
Lived Comfortably	1.24	1.00
Found it Difficult	1.18	1.00
Found it Very Difficult	1.14	1.00
Childhood Abuse (Ref: No)		
Yes	1.12	1.00
Outsider (Ref: No)		
Yes	1.19	1.00
Childhood Health (Ref: Good)		
Excellent	1.22	1.00
Very Good	1.12	1.00

Fair	1.13	1.00
Poor	1.34	1.00
Immigration Status (Ref: Born in This Country)		
Born in Another Country	1.65	1.00
Childhood Service Attendance (Ref: Never)		
At Least 1/Week	1.33	1.09
1-3/Month	1.09	1.00
<1/Month	1.13	1.00
Gender (Ref: Male)		
Female	1.45	1.27
Other	1.00	1.00
Year of Birth (Ref: 1998-2005)		
1993-1998; Age 25-29	1.29	1.00
1983-1993; Age 30-39	1.26	1.00
1973-1983; Age 40-49	1.16	1.00
1963-1973; Age 50-59	1.45	1.17
1953-1963; Age 60-69	1.70	1.39
1943-1953; Age 70-79	1.42	1.00
1943 or Earlier; Age 80 or Older	1.43	1.00
Mother Absence/Presence (Ref: Present)		
Absent	1.25	1.00
Father Absence/Presence (Ref: Present)		
Absent	1.11	1.00
Childhood Religion (Ref: Islam)		
Some Other Religion	1.27	1.00
Race/Ethnicity (Ref: Ethnic Plurality)		
Ethnic Minority	1.68	1.00

Table S5a: Nationally Representative Descriptive Statistics of the Observed Sample (Germany)

Variable	Proportion	Frequency
Relationship with Mother		
Very Good	0.58	5497
Somewhat Good	0.32	3031
Somewhat Bad	0.05	496
Very Bad	0.02	187
Not Applicable	0.03	241
Missing	0.01	54
Relationship with Father		
Very Good	0.49	4652
Somewhat Good	0.32	3012
Somewhat Bad	0.09	846
Very Bad	0.04	385
Not Applicable	0.06	538
Missing	0.01	73
Parent Marital Status		
Married	0.80	7620
Divorced	0.10	927

Never Married	0.06	578
One or Both Had Died	0.03	245
Missing	0.01	136
Childhood Income		
Lived Comfortably	0.33	3177
Got By	0.47	4508
Found it Difficult	0.16	1481
Found it Very Difficult	0.03	314
Missing	0.00	26
Childhood Abuse		
Yes	0.11	1086
No	0.88	8321
Missing	0.01	99
Outsider		
Yes	0.12	1105
No	0.87	8262
Not Applicable	0.01	114
Missing	0.00	25
Childhood Health		
Excellent	0.28	2633
Very Good	0.37	3518
Good	0.27	2582
Fair	0.06	612
Poor	0.01	134
Missing	0.00	26
Immigration Status		
Born in This Country	0.92	8722
Born in Another Country	0.08	744
Missing	0.00	40
Childhood Service Attendance		
At Least 1/Week	0.20	1943
1-3/Month	0.20	1899
<1/Month	0.30	2887
Never	0.29	2749
Missing	0.00	27
Gender		
Male	0.49	4641
Female	0.51	4843
Other	0.00	11
Missing	0.00	11
Year of Birth		
1998-2005; Age 18-24	0.09	829
1993-1998; Age 25-29	0.08	774
1983-1993; Age 30-39	0.15	1438
1973-1983; Age 40-49	0.16	1494
1963-1973; Age 50-59	0.18	1729
1953-1963; Age 60-69	0.20	1915
1943-1953; Age 70-79	0.12	1137
1943 or Earlier; 80 or Older	0.02	190

Missing	.	.
Childhood Religion		
Christianity	0.61	5751
Islam	0.04	350
Hinduism	0.00	15
Buddhism	0.00	25
Judaism	0.00	18
Sikhism	0.00	5
Baha'i	0.00	2
Jainism	0.00	1
Shinto	.	.
Taoism	.	.
Confucianism	0.00	4
Primal, Animist, or Folk Religion	0.00	19
Spiritism	.	.
African-Derived	.	.
Chinese	.	.
Some Other Religion	0.01	67
No Religion/Atheist/Agnostic	0.33	3163
Missing	0.01	85
Race/Ethnicity		
No Data	.	.

Table S5b: Variations Across Childhood Predictors (Germany)

Variable	IRR	SE	Prob	LCI	UCI	Global p-value
Relationship with Mother (Ref: Very/Somewhat Bad)						
Very/Somewhat Good	0.78	0.14	0.17	0.56	1.11	0.17
Relationship with Father (Ref: Very/Somewhat Bad)						
Very/Somewhat Good	1.37	0.22	0.05	1.00	1.88	0.05
Parent Marital Status (Ref: Married)						
Divorced	0.89	0.15	0.48	0.64	1.24	0.75
Never Married	1.07	0.20	0.71	0.74	1.56	.
One or Both Had Died	0.79	0.25	0.46	0.43	1.47	.
Childhood Income (Ref: Got By)						
Lived Comfortably	1.11	0.13	0.38	0.88	1.39	0.07
Found it Difficult	1.11	0.16	0.49	0.83	1.47	.
Found it Very Difficult	0.42	0.16	0.02	0.20	0.88	.
Childhood Abuse (Ref: No)						
Yes	1.26	0.17	0.09	0.96	1.65	0.09
Outsider (Ref: No)						
Yes	1.15	0.15	0.28	0.89	1.49	0.28
Childhood Health (Ref: Good)						
Excellent	0.91	0.13	0.51	0.69	1.20	0.87
Very Good	0.95	0.12	0.69	0.74	1.22	.

Fair	1.02	0.21	0.91	0.69	1.51	.
Poor	1.32	0.48	0.44	0.65	2.70	.
Immigration Status (Ref: Born in This Country)						
Born in Another Country	1.14	0.21	0.46	0.80	1.63	0.46
Childhood Service Attendance (Ref: Never)						
At Least 1/Week	4.15	0.68	0.00	3.00	5.73	0.00
1-3/Month	3.05	0.53	0.00	2.18	4.28	.
<1/Month	1.23	0.23	0.27	0.85	1.76	.
Gender (Ref: Male)						
Female	0.88	0.09	0.21	0.73	1.07	0.00
Other	0.00	0.00	0.00	0.00	0.00	.
Year of Birth (Ref: 1998-2005)						
1993-1998; Age 25-29	0.88	0.20	0.56	0.56	1.37	0.42
1983-1993; Age 30-39	0.88	0.17	0.50	0.60	1.28	.
1973-1983; Age 40-49	0.87	0.17	0.48	0.59	1.28	.
1963-1973; Age 50-59	0.69	0.14	0.07	0.46	1.03	.
1953-1963; Age 60-69	0.75	0.15	0.17	0.50	1.12	.
1943-1953; Age 70-79	1.00	0.22	0.98	0.65	1.52	.
1943 or Earlier; Age 80 or Older	1.05	0.32	0.87	0.58	1.91	.
Mother Absence/Presence (Ref: Present)						
Absent	1.03	0.23	0.88	0.67	1.59	0.88
Father Absence/Presence (Ref: Present)						
Absent	1.18	0.26	0.45	0.77	1.82	0.45
Childhood Religion (Ref: No Religion/Atheist/Agnostic)						
Christianity	1.31	0.18	0.05	1.00	1.72	0.00
Islam	3.52	0.68	0.00	2.41	5.14	.
Some Other Religion	2.51	0.83	0.01	1.31	4.82	.

Table S5c: E-Values and E-Value Limits (Germany)

Variable	E-Value	E-Value Limit
Relationship with Mother (Ref: Very/Somewhat Bad)		
Very/Somewhat Good	2.54	1.00
Relationship with Father (Ref: Very/Somewhat Bad)		
Very/Somewhat Good	3.03	1.04
Parent Marital Status (Ref: Married)		
Divorced	1.81	1.00
Never Married	1.55	1.00
One or Both Had Died	2.47	1.00
Childhood Income (Ref: Got By)		
Lived Comfortably	1.71	1.00
Found it Difficult	1.71	1.00
Found it Very Difficult	9.89	1.32
Childhood Abuse (Ref: No)		
Yes	2.47	1.00

Outsider (Ref: No)		
Yes	1.93	1.00
Childhood Health (Ref: Good)		
Excellent	1.66	1.00
Very Good	1.43	1.00
Fair	1.25	1.00
Poor	2.78	1.00
Immigration Status (Ref: Born in This Country)		
Born in Another Country	1.90	1.00
Childhood Service Attendance (Ref: Never)		
At Least 1/Week	28.72	2.86
1-3/Month	15.86	2.31
<1/Month	2.30	1.00
Gender (Ref: Male)		
Female	1.84	1.00
Other	1.12e+11	555.56
Year of Birth (Ref: 1998-2005)		
1993-1998; Age 25-29	1.88	1.00
1983-1993; Age 30-39	1.88	1.00
1973-1983; Age 40-49	1.92	1.00
1963-1973; Age 50-59	3.48	1.00
1953-1963; Age 60-69	2.81	1.00
1943-1953; Age 70-79	1.10	1.00
1943 or Earlier; Age 80 or Older	1.44	1.00
Mother Absence/Presence (Ref: Present)		
Absent	1.33	1.00
Father Absence/Presence (Ref: Present)		
Absent	2.07	1.00
Childhood Religion (Ref: No Religion/Atheist/Agnostic)		
Christianity	2.72	1.00
Islam	20.95	2.48
Some Other Religion	10.81	1.55

Table S6a: Nationally Representative Descriptive Statistics of the Observed Sample (Hong Kong)

Variable	Proportion	Frequency
Relationship with Mother		
Very Good	0.36	1077
Somewhat Good	0.39	1164
Somewhat Bad	0.10	293
Very Bad	0.02	49
Not Applicable	0.14	426
Missing	0.00	3
Relationship with Father		
Very Good	0.29	868
Somewhat Good	0.36	1089
Somewhat Bad	0.13	393

Very Bad	0.03	102
Not Applicable	0.19	557
Missing	0.00	3
Parent Marital Status		
Married	0.91	2752
Divorced	0.04	114
Never Married	0.01	40
One or Both Had Died	0.02	50
Missing	0.02	56
Childhood Income		
Lived Comfortably	0.30	906
Got By	0.51	1527
Found it Difficult	0.16	473
Found it Very Difficult	0.03	84
Missing	0.01	22
Childhood Abuse		
Yes	0.11	318
No	0.89	2688
Missing	0.00	5
Outsider		
Yes	0.22	664
No	0.74	2224
Not Applicable	0.04	110
Missing	0.00	14
Childhood Health		
Excellent	0.18	545
Very Good	0.36	1073
Good	0.29	863
Fair	0.14	426
Poor	0.03	91
Missing	0.00	13
Immigration Status		
Born in This Country	0.88	2637
Born in Another Country	0.11	321
Missing	0.02	53
Childhood Service Attendance		
At Least 1/Week	0.14	432
1-3/Month	0.18	528
<1/Month	0.25	753
Never	0.43	1295
Missing	0.00	4
Gender		
Male	0.46	1390
Female	0.54	1620
Other	0.00	2
Missing	.	.
Year of Birth		
1998-2005; Age 18-24	0.07	217
1993-1998; Age 25-29	0.07	198

1983-1993; Age 30-39	0.17	507
1973-1983; Age 40-49	0.19	580
1963-1973; Age 50-59	0.24	711
1953-1963; Age 60-69	0.21	620
1943-1953; Age 70-79	0.05	164
1943 or Earlier; 80 or Older	0.00	15
Missing	.	.
Childhood Religion		
Christianity	0.24	715
Islam	0.03	86
Hinduism	0.01	27
Buddhism	0.11	323
Judaism	0.01	16
Sikhism	0.00	4
Baha'i	.	.
Jainism	0.00	1
Shinto	0.01	18
Taoism	0.03	81
Confucianism	0.00	10
Primal, Animist, or Folk Religion	0.00	15
Spiritism	.	.
African-Derived	.	.
Chinese	0.04	108
Some Other Religion	0.00	5
No Religion/Atheist/Agnostic	0.53	1601
Missing	0.00	1
Race/Ethnicity		
Chinese (Cantonese)	0.64	1930
Chinese (Chaoshan)	0.07	201
Chinese (Fujianese)	0.04	117
Chinese (Hakka)	0.04	121
Chinese (Shanghainese)	0.03	89
Chinese (Other Ethnicity)	0.09	264
East Asian (Korean, Japanese)	0.00	10
Southeast Asian (Filipino, Indonesian, Thailand)	0.02	46
South Asian (Indian, Nepalese, Pakistani)	0.01	17
Taiwanese	0.00	14
White	0.00	15
Other	0.00	4
Missing	0.06	184

Table S6b: Variations Across Childhood Predictors (Hong Kong)

Variable	IRR	SE	Prob	LCI	UCI	Global p-value
Relationship with Mother (Ref: Very/Somewhat Bad)						

Very/Somewhat Good	1.12	0.12	0.32	0.90	1.38	0.32
Relationship with Father (Ref: Very/Somewhat Bad)						
Very/Somewhat Good	0.78	0.09	0.04	0.61	0.99	0.04
Parent Marital Status (Ref: Married)						
Divorced	1.09	0.27	0.73	0.68	1.76	0.34
Never Married	1.40	0.34	0.17	0.86	2.27	.
One or Both Had Died	0.60	0.26	0.24	0.25	1.42	.
Childhood Income (Ref: Got By)						
Lived Comfortably	1.04	0.07	0.55	0.91	1.19	0.43
Found it Difficult	0.87	0.14	0.39	0.64	1.19	.
Found it Very Difficult	0.59	0.24	0.20	0.27	1.31	.
Childhood Abuse (Ref: No)						
Yes	1.41	0.11	0.00	1.21	1.64	0.00
Outsider (Ref: No)						
Yes	1.26	0.08	0.00	1.11	1.43	0.00
Childhood Health (Ref: Good)						
Excellent	1.33	0.15	0.01	1.07	1.66	0.00
Very Good	1.21	0.11	0.04	1.01	1.45	.
Fair	0.39	0.09	0.00	0.24	0.62	.
Poor	0.49	0.19	0.07	0.22	1.05	.
Immigration Status (Ref: Born in This Country)						
Born in Another Country	0.70	0.11	0.03	0.51	0.97	0.03
Childhood Service Attendance (Ref: Never)						
At Least 1/Week	8.89	2.28	0.00	5.37	14.70	0.00
1-3/Month	8.13	1.97	0.00	5.05	13.08	.
<1/Month	3.51	0.84	0.00	2.20	5.61	.
Gender (Ref: Male)						
Female	1.04	0.06	0.56	0.92	1.16	0.00
Other	0.00	0.00	0.00	0.00	0.00	.
Year of Birth (Ref: 1998-2005)						
1993-1998; Age 25-29	0.94	0.13	0.64	0.72	1.23	0.00
1983-1993; Age 30-39	1.00	0.12	0.99	0.79	1.26	.
1973-1983; Age 40-49	1.09	0.12	0.41	0.88	1.36	.
1963-1973; Age 50-59	1.31	0.13	0.01	1.07	1.60	.
1953-1963; Age 60-69	1.36	0.15	0.01	1.09	1.69	.
1943-1953; Age 70-79	1.20	0.31	0.48	0.73	1.98	.
1943 or Earlier; Age 80 or Older	0.00	0.00	0.00	0.00	0.00	.
Mother Absence/Presence (Ref: Present)						
Absent	1.46	0.18	0.00	1.15	1.85	0.00
Father Absence/Presence (Ref: Present)						
Absent	0.60	0.08	0.00	0.46	0.79	0.00
Childhood Religion (Ref: No Religion/Atheist/Agnostic)						
Christianity	2.20	0.32	0.00	1.65	2.93	0.00
Buddhism	1.93	0.30	0.00	1.42	2.61	.
Chinese	2.40	0.38	0.00	1.77	3.27	.
Some Other Religion	2.27	0.36	0.00	1.67	3.10	.

Race/Ethnicity (Ref: Ethnic Plurality)						
Ethnic Minority	0.91	0.06	0.15	0.80	1.04	0.15

Table S6c: E-Values and E-Value Limits (Hong Kong)

Variable	E-Value	E-Value Limit
Relationship with Mother (Ref: Very/Somewhat Bad)		
Very/Somewhat Good	1.47	1.00
Relationship with Father (Ref: Very/Somewhat Bad)		
Very/Somewhat Good	1.86	1.08
Parent Marital Status (Ref: Married)		
Divorced	1.39	1.00
Never Married	2.12	1.00
One or Both Had Died	2.68	1.00
Childhood Income (Ref: Got By)		
Lived Comfortably	1.24	1.00
Found it Difficult	1.54	1.00
Found it Very Difficult	2.71	1.00
Childhood Abuse (Ref: No)		
Yes	2.14	1.43
Outsider (Ref: No)		
Yes	1.82	1.29
Childhood Health (Ref: Good)		
Excellent	1.97	1.22
Very Good	1.69	1.06
Fair	4.48	1.85
Poor	3.45	1.00
Immigration Status (Ref: Born in This Country)		
Born in Another Country	2.17	1.15
Childhood Service Attendance (Ref: Never)		
At Least 1/Week	16.23	4.07
1-3/Month	14.83	3.92
<1/Month	6.24	2.33
Gender (Ref: Male)		
Female	1.22	1.00
Other	855582.81	647.45
Year of Birth (Ref: 1998-2005)		
1993-1998; Age 25-29	1.33	1.00
1983-1993; Age 30-39	1.04	1.00
1973-1983; Age 40-49	1.41	1.00
1963-1973; Age 50-59	1.92	1.22
1953-1963; Age 60-69	2.03	1.26
1943-1953; Age 70-79	1.67	1.00
1943 or Earlier; Age 80 or Older	2.96e+06	1439.33
Mother Absence/Presence (Ref: Present)		
Absent	2.24	1.35
Father Absence/Presence (Ref: Present)		

Absent	2.65	1.50
Childhood Religion (Ref: No Religion/Atheist/Agnostic)		
Christianity	3.72	1.89
Buddhism	3.19	1.67
Chinese	4.13	1.99
Some Other Religion	3.87	1.90
Race/Ethnicity (Ref: Ethnic Plurality)		
Ethnic Minority	1.43	1.00

Table S7a: Nationally Representative Descriptive Statistics of the Observed Sample (India)

Variable	Proportion	Frequency
Relationship with Mother		
Very Good	0.90	11465
Somewhat Good	0.06	788
Somewhat Bad	0.01	88
Very Bad	0.01	73
Not Applicable	0.02	269
Missing	0.01	82
Relationship with Father		
Very Good	0.86	10923
Somewhat Good	0.08	995
Somewhat Bad	0.01	126
Very Bad	0.01	100
Not Applicable	0.04	481
Missing	0.01	140
Parent Marital Status		
Married	0.44	5578
Divorced	0.02	236
Never Married	0.08	1055
One or Both Had Died	0.07	940
Missing	0.39	4956
Childhood Income		
Lived Comfortably	0.39	4946
Got By	0.24	3010
Found it Difficult	0.21	2703
Found it Very Difficult	0.16	2035
Missing	0.01	70
Childhood Abuse		
Yes	0.11	1468
No	0.82	10526
Missing	0.06	771
Outsider		
Yes	0.15	1926
No	0.84	10780
Not Applicable	0.00	15
Missing	0.00	44
Childhood Health		

Excellent	0.17	2182
Very Good	0.30	3882
Good	0.32	4028
Fair	0.17	2202
Poor	0.03	424
Missing	0.00	47
Immigration Status		
Born in This Country	0.99	12629
Born in Another Country	0.01	110
Missing	0.00	26
Childhood Service Attendance		
At Least 1/Week	0.41	5288
1-3/Month	0.23	2959
<1/Month	0.21	2719
Never	0.12	1478
Missing	0.03	321
Gender		
Male	0.51	6473
Female	0.49	6292
Other	.	.
Missing	.	.
Year of Birth		
1998-2005; Age 18-24	0.20	2543
1993-1998; Age 25-29	0.13	1640
1983-1993; Age 30-39	0.24	3109
1973-1983; Age 40-49	0.18	2275
1963-1973; Age 50-59	0.12	1574
1953-1963; Age 60-69	0.09	1188
1943-1953; Age 70-79	0.03	370
1943 or Earlier; 80 or Older	0.01	67
Missing	.	.
Childhood Religion		
Christianity	0.02	254
Islam	0.12	1550
Hinduism	0.82	10417
Buddhism	0.01	180
Judaism	.	.
Sikhism	0.01	126
Baha'i	.	.
Jainism	0.00	9
Shinto	0.00	4
Taoism	.	.
Confucianism	.	.
Primal, Animist, or Folk Religion	0.00	27
Spiritism	.	.
African-Derived	.	.
Chinese	.	.
Some Other Religion	0.00	59
No Religion/Atheist/Agnostic	0.00	7

Missing	0.01	131
Race/Ethnicity		
General	0.28	3538
Other Backward Caste	0.33	4177
Schedule Caste	0.28	3599
Schedule Tribe	0.09	1185
Other	.	.
Missing	0.02	267

Table S7b: Variations Across Childhood Predictors (India)

Variable	IRR	SE	Prob	LCI	UCI	Global p-value
Relationship with Mother (Ref: Very/Somewhat Bad)						
Very/Somewhat Good	0.98	0.11	0.89	0.79	1.23	0.89
Relationship with Father (Ref: Very/Somewhat Bad)						
Very/Somewhat Good	1.14	0.11	0.19	0.94	1.38	0.19
Parent Marital Status (Ref: Married)						
Divorced	1.19	0.09	0.03	1.02	1.38	0.09
Never Married	0.89	0.06	0.13	0.76	1.04	.
One or Both Had Died	1.01	0.06	0.87	0.89	1.14	.
Childhood Income (Ref: Got By)						
Lived Comfortably	1.02	0.04	0.54	0.95	1.10	0.00
Found it Difficult	0.95	0.04	0.26	0.88	1.04	.
Found it Very Difficult	0.82	0.05	0.00	0.73	0.92	.
Childhood Abuse (Ref: No)						
Yes	1.24	0.05	0.00	1.15	1.35	0.00
Outsider (Ref: No)						
Yes	1.05	0.05	0.28	0.96	1.14	0.28
Childhood Health (Ref: Good)						
Excellent	0.97	0.05	0.53	0.88	1.07	0.19
Very Good	1.04	0.04	0.29	0.97	1.12	.
Fair	0.95	0.04	0.28	0.87	1.04	.
Poor	1.07	0.08	0.36	0.92	1.25	.
Immigration Status (Ref: Born in This Country)						
Born in Another Country	1.23	0.22	0.25	0.86	1.76	0.25
Childhood Service Attendance (Ref: Never)						
At Least 1/Week	1.20	0.06	0.00	1.08	1.33	0.00
1-3/Month	1.14	0.06	0.02	1.02	1.27	.
<1/Month	1.05	0.06	0.43	0.93	1.17	.
Gender (Ref: Male)						
Female	1.03	0.03	0.28	0.97	1.10	0.28
Other	1.00	.	.	.	.	.
Year of Birth (Ref: 1998-2005)						

1993-1998; Age 25-29	1.00	0.05	0.97	0.90	1.11	0.05
1983-1993; Age 30-39	1.03	0.05	0.57	0.94	1.12	.
1973-1983; Age 40-49	1.07	0.05	0.12	0.98	1.18	.
1963-1973; Age 50-59	1.09	0.06	0.14	0.97	1.21	.
1953-1963; Age 60-69	1.17	0.07	0.01	1.05	1.32	.
1943-1953; Age 70-79	1.10	0.10	0.29	0.92	1.32	.
1943 or Earlier; Age 80 or Older	1.44	0.25	0.04	1.02	2.03	.
Mother Absence/Presence (Ref: Present)						
Absent	0.97	0.12	0.80	0.77	1.23	0.80
Father Absence/Presence (Ref: Present)						
Absent	1.02	0.10	0.84	0.84	1.24	0.84
Childhood Religion (Ref: Hinduism)						
Islam	1.29	0.06	0.00	1.19	1.41	0.00
Some Other Religion	1.18	0.07	0.01	1.05	1.33	.
Race/Ethnicity (Ref: Ethnic Plurality)						
Ethnic Minority	0.89	0.03	0.00	0.83	0.95	0.00

Table S7c: E-Values and E-Value Limits (India)

Variable	E-Value	E-Value Limit
Relationship with Mother (Ref: Very/Somewhat Bad)		
Very/Somewhat Good	1.13	1.00
Relationship with Father (Ref: Very/Somewhat Bad)		
Very/Somewhat Good	1.47	1.00
Parent Marital Status (Ref: Married)		
Divorced	1.57	1.11
Never Married	1.43	1.00
One or Both Had Died	1.10	1.00
Childhood Income (Ref: Got By)		
Lived Comfortably	1.16	1.00
Found it Difficult	1.25	1.00
Found it Very Difficult	1.64	1.26
Childhood Abuse (Ref: No)		
Yes	1.69	1.35
Outsider (Ref: No)		
Yes	1.24	1.00
Childhood Health (Ref: Good)		
Excellent	1.19	1.00
Very Good	1.22	1.00
Fair	1.25	1.00
Poor	1.31	1.00
Immigration Status (Ref: Born in This Country)		
Born in Another Country	1.66	1.00
Childhood Service Attendance (Ref: Never)		
At Least 1/Week	1.60	1.24
1-3/Month	1.46	1.10
<1/Month	1.24	1.00

Gender (Ref: Male)		
Female	1.20	1.00
Other	1.00	1.00
Year of Birth (Ref: 1998-2005)		
1993-1998; Age 25-29	1.04	1.00
1983-1993; Age 30-39	1.17	1.00
1973-1983; Age 40-49	1.32	1.00
1963-1973; Age 50-59	1.34	1.00
1953-1963; Age 60-69	1.54	1.17
1943-1953; Age 70-79	1.38	1.00
1943 or Earlier; Age 80 or Older	2.04	1.10
Mother Absence/Presence (Ref: Present)		
Absent	1.19	1.00
Father Absence/Presence (Ref: Present)		
Absent	1.15	1.00
Childhood Religion (Ref: Hinduism)		
Islam	1.78	1.40
Some Other Religion	1.56	1.19
Race/Ethnicity (Ref: Ethnic Plurality)		
Ethnic Minority	1.45	1.20

Table S8a: Nationally Representative Descriptive Statistics of the Observed Sample (Indonesia)

Variable	Proportion	Frequency
Relationship with Mother		
Very Good	0.89	6238
Somewhat Good	0.08	583
Somewhat Bad	0.01	50
Very Bad	0.00	26
Not Applicable	0.01	68
Missing	0.00	27
Relationship with Father		
Very Good	0.87	6067
Somewhat Good	0.09	628
Somewhat Bad	0.01	68
Very Bad	0.01	52
Not Applicable	0.02	115
Missing	0.01	61
Parent Marital Status		
Married	0.79	5557
Divorced	0.06	448
Never Married	0.01	47
One or Both Had Died	0.11	735
Missing	0.03	205
Childhood Income		
Lived Comfortably	0.49	3408
Got By	0.42	2955
Found it Difficult	0.06	439

Found it Very Difficult	0.03	181
Missing	0.00	9
Childhood Abuse		
Yes	0.07	486
No	0.92	6427
Missing	0.01	79
Outsider		
Yes	0.05	343
No	0.95	6639
Not Applicable	0.00	1
Missing	0.00	9
Childhood Health		
Excellent	0.18	1246
Very Good	0.28	1968
Good	0.36	2490
Fair	0.18	1233
Poor	0.01	55
Missing	0.00	1
Immigration Status		
Born in This Country	1.00	6958
Born in Another Country	0.00	34
Missing	.	.
Childhood Service Attendance		
At Least 1/Week	0.77	5363
1-3/Month	0.14	973
<1/Month	0.05	329
Never	0.04	275
Missing	0.01	51
Gender		
Male	0.50	3461
Female	0.50	3513
Other	0.00	7
Missing	0.00	11
Year of Birth		
1998-2005; Age 18-24	0.17	1216
1993-1998; Age 25-29	0.12	849
1983-1993; Age 30-39	0.23	1591
1973-1983; Age 40-49	0.23	1576
1963-1973; Age 50-59	0.17	1169
1953-1963; Age 60-69	0.07	490
1943-1953; Age 70-79	0.01	83
1943 or Earlier; 80 or Older	0.00	17
Missing	.	.
Childhood Religion		
Christianity	0.08	528
Islam	0.91	6373
Hinduism	0.01	75
Buddhism	0.00	5
Judaism	.	.

Sikhism	.	.
Baha'i	.	.
Jainism	0.00	1
Shinto	.	.
Taoism	0.00	0
Confucianism	0.00	1
Primal, Animist, or Folk Religion	0.00	1
Spiritism	.	.
African-Derived	.	.
Chinese	.	.
Some Other Religion	.	.
No Religion/Atheist/Agnostic	0.00	2
Missing	0.00	8
Race/Ethnicity		
Banjar/Melayu Banjar	0.05	320
Betawi	0.04	251
Bugis	0.03	243
Jawa	0.41	2846
Madura	0.04	262
Minangkabau	0.04	273
Sunda/Parahyangan	0.17	1172
Bali	0.01	69
Batak	0.02	165
Makasar	0.01	91
Other	0.18	1262
Missing	0.01	38

Table S8b: Variations Across Childhood Predictors (Indonesia)

Variable	IRR	SE	Prob	LCI	UCI	Global p-value
Relationship with Mother (Ref: Very/Somewhat Bad)						
Very/Somewhat Good	1.12	0.14	0.36	0.87	1.45	0.36
Relationship with Father (Ref: Very/Somewhat Bad)						
Very/Somewhat Good	0.93	0.08	0.36	0.79	1.09	0.36
Parent Marital Status (Ref: Married)						
Divorced	1.04	0.05	0.35	0.96	1.14	0.27
Never Married	1.02	0.17	0.89	0.74	1.41	.
One or Both Had Died	1.07	0.04	0.07	0.99	1.16	.
Childhood Income (Ref: Got By)						
Lived Comfortably	1.03	0.03	0.34	0.97	1.09	0.80
Found it Difficult	1.03	0.06	0.60	0.92	1.16	.
Found it Very Difficult	1.01	0.09	0.94	0.84	1.21	.
Childhood Abuse (Ref: No)						
Yes	0.94	0.05	0.27	0.85	1.05	0.27
Outsider (Ref: No)						

Yes	0.99	0.06	0.87	0.87	1.12	0.87
Childhood Health (Ref: Good)						
Excellent	1.17	0.04	0.00	1.09	1.25	0.00
Very Good	1.13	0.03	0.00	1.07	1.20	.
Fair	1.04	0.04	0.22	0.97	1.12	.
Poor	0.91	0.18	0.65	0.62	1.34	.
Immigration Status (Ref: Born in This Country)						
Born in Another Country	1.05	0.25	0.83	0.66	1.69	0.83
Childhood Service Attendance (Ref: Never)						
At Least 1/Week	1.27	0.10	0.00	1.09	1.49	0.00
1-3/Month	1.16	0.09	0.08	0.98	1.36	.
<1/Month	0.86	0.10	0.19	0.69	1.07	.
Gender (Ref: Male)						
Female	1.10	0.03	0.00	1.05	1.16	0.00
Other	1.44	0.21	0.01	1.08	1.92	.
Year of Birth (Ref: 1998-2005)						
1993-1998; Age 25-29	0.97	0.05	0.54	0.88	1.07	0.01
1983-1993; Age 30-39	1.03	0.04	0.53	0.94	1.12	.
1973-1983; Age 40-49	1.04	0.04	0.39	0.95	1.13	.
1963-1973; Age 50-59	1.13	0.05	0.01	1.03	1.24	.
1953-1963; Age 60-69	1.15	0.08	0.03	1.01	1.31	.
1943-1953; Age 70-79	1.25	0.13	0.03	1.02	1.53	.
1943 or Earlier; Age 80 or Older	1.22	0.41	0.56	0.63	2.37	.
Mother Absence/Presence (Ref: Present)						
Absent	1.06	0.13	0.61	0.84	1.34	0.61
Father Absence/Presence (Ref: Present)						
Absent	0.96	0.09	0.68	0.79	1.16	0.68
Childhood Religion (Ref: Islam)						
Christianity	0.66	0.05	0.00	0.56	0.77	0.00
Some Other Religion	0.63	0.08	0.00	0.48	0.82	.
Race/Ethnicity (Ref: Ethnic Plurality)						
Ethnic Minority	0.99	0.03	0.81	0.94	1.05	0.81

Table S8c: E-Values and E-Value Limits (Indonesia)

Variable	E-Value	E-Value Limit
Relationship with Mother (Ref: Very/Somewhat Bad)		
Very/Somewhat Good	1.37	1.00
Relationship with Father (Ref: Very/Somewhat Bad)		
Very/Somewhat Good	1.28	1.00
Parent Marital Status (Ref: Married)		
Divorced	1.20	1.00
Never Married	1.14	1.00
One or Both Had Died	1.27	1.00
Childhood Income (Ref: Got By)		

Lived Comfortably	1.15	1.00
Found it Difficult	1.17	1.00
Found it Very Difficult	1.07	1.00
Childhood Abuse (Ref: No)		
Yes	1.24	1.00
Outsider (Ref: No)		
Yes	1.09	1.00
Childhood Health (Ref: Good)		
Excellent	1.44	1.25
Very Good	1.38	1.22
Fair	1.20	1.00
Poor	1.31	1.00
Immigration Status (Ref: Born in This Country)		
Born in Another Country	1.22	1.00
Childhood Service Attendance (Ref: Never)		
At Least 1/Week	1.61	1.25
1-3/Month	1.43	1.00
<1/Month	1.43	1.00
Gender (Ref: Male)		
Female	1.33	1.18
Other	1.84	1.23
Year of Birth (Ref: 1998-2005)		
1993-1998; Age 25-29	1.16	1.00
1983-1993; Age 30-39	1.15	1.00
1973-1983; Age 40-49	1.18	1.00
1963-1973; Age 50-59	1.39	1.14
1953-1963; Age 60-69	1.43	1.09
1943-1953; Age 70-79	1.58	1.11
1943 or Earlier; Age 80 or Older	1.53	1.00
Mother Absence/Presence (Ref: Present)		
Absent	1.25	1.00
Father Absence/Presence (Ref: Present)		
Absent	1.19	1.00
Childhood Religion (Ref: Islam)		
Christianity	1.96	1.55
Some Other Religion	2.04	1.45
Race/Ethnicity (Ref: Ethnic Plurality)		
Ethnic Minority	1.07	1.00

Table S9a: Nationally Representative Descriptive Statistics of the Observed Sample (Israel)

Variable	Proportion	Frequency
Relationship with Mother		
Very Good	0.73	2686
Somewhat Good	0.22	793
Somewhat Bad	0.03	110
Very Bad	0.00	18
Not Applicable	0.01	45

Missing	0.00	17
Relationship with Father		
Very Good	0.62	2290
Somewhat Good	0.25	912
Somewhat Bad	0.06	234
Very Bad	0.01	37
Not Applicable	0.05	171
Missing	0.01	25
Parent Marital Status		
Married	0.86	3172
Divorced	0.08	284
Never Married	0.01	36
One or Both Had Died	0.04	130
Missing	0.01	47
Childhood Income		
Lived Comfortably	0.25	923
Got By	0.50	1822
Found it Difficult	0.18	667
Found it Very Difficult	0.07	239
Missing	0.00	17
Childhood Abuse		
Yes	.	.
No	.	.
Missing	.	.
Outsider		
Yes	0.10	371
No	0.88	3228
Not Applicable	0.01	36
Missing	0.01	34
Childhood Health		
Excellent	0.49	1785
Very Good	0.35	1284
Good	0.13	480
Fair	0.03	105
Poor	0.00	6
Missing	0.00	8
Immigration Status		
Born in This Country	0.76	2796
Born in Another Country	0.24	868
Missing	0.00	5
Childhood Service Attendance		
At Least 1/Week	0.24	867
1-3/Month	0.12	435
<1/Month	0.22	810
Never	0.42	1539
Missing	0.00	17
Gender		
Male	0.49	1791
Female	0.51	1872

Other	0.00	0
Missing	0.00	6
Year of Birth		
1998-2005; Age 18-24	0.15	553
1993-1998; Age 25-29	0.11	407
1983-1993; Age 30-39	0.18	666
1973-1983; Age 40-49	0.17	616
1963-1973; Age 50-59	0.15	542
1953-1963; Age 60-69	0.13	469
1943-1953; Age 70-79	0.09	336
1943 or Earlier; 80 or Older	0.02	79
Missing	.	.
Childhood Religion		
Christianity	0.02	60
Islam	0.18	647
Hinduism	.	.
Buddhism	.	.
Judaism	0.78	2873
Sikhism	0.00	1
Baha'i	0.00	1
Jainism	.	.
Shinto	.	.
Taoism	.	.
Confucianism	.	.
Primal, Animist, or Folk Religion	0.00	3
Spiritism	.	.
African-Derived	.	.
Chinese	.	.
Some Other Religion	0.00	5
No Religion/Atheist/Agnostic	0.02	69
Missing	0.00	10
Race/Ethnicity		
Jewish	0.80	2926
Arab	0.18	674
Other	0.01	39
Missing	0.01	30

Table S9b: Variations Across Childhood Predictors (Israel)

Variable	IRR	SE	Prob	LCI	UCI	Global p-value
Relationship with Mother (Ref: Very/Somewhat Bad)						
Very/Somewhat Good	0.96	0.16	0.82	0.70	1.33	0.82
Relationship with Father (Ref: Very/Somewhat Bad)						
Very/Somewhat Good	0.92	0.11	0.50	0.72	1.18	0.50
Parent Marital Status (Ref: Married)						

Divorced	0.72	0.11	0.03	0.54	0.97	0.00
Never Married	1.65	0.36	0.03	1.06	2.55	.
One or Both Had Died	0.57	0.10	0.00	0.40	0.82	.
Childhood Income (Ref: Got By)						
Lived Comfortably	0.91	0.08	0.25	0.77	1.07	0.02
Found it Difficult	1.21	0.09	0.01	1.05	1.39	.
Found it Very Difficult	1.10	0.15	0.49	0.84	1.43	.
Childhood Abuse (Ref: No)						
Yes	1.00	.	.	.	.	.
Outsider (Ref: No)						
Yes	0.93	0.09	0.43	0.77	1.12	0.43
Childhood Health (Ref: Good)						
Excellent	1.49	0.19	0.00	1.15	1.92	0.00
Very Good	1.35	0.15	0.01	1.07	1.69	.
Fair	0.76	0.19	0.26	0.46	1.24	.
Poor	4.90	1.07	0.00	3.18	7.55	.
Immigration Status (Ref: Born in This Country)						
Born in Another Country	0.72	0.08	0.00	0.59	0.89	0.00
Childhood Service Attendance (Ref: Never)						
At Least 1/Week	3.83	0.42	0.00	3.08	4.76	0.00
1-3/Month	2.90	0.32	0.00	2.32	3.62	.
<1/Month	1.57	0.19	0.00	1.22	2.00	.
Gender (Ref: Male)						
Female	1.14	0.07	0.05	1.00	1.29	0.00
Other	0.00	0.00	0.00	0.00	0.00	.
Year of Birth (Ref: 1998-2005)						
1993-1998; Age 25-29	0.88	0.08	0.16	0.74	1.05	0.10
1983-1993; Age 30-39	0.81	0.07	0.02	0.68	0.96	.
1973-1983; Age 40-49	0.81	0.08	0.03	0.66	0.98	.
1963-1973; Age 50-59	0.92	0.08	0.35	0.78	1.09	.
1953-1963; Age 60-69	0.74	0.08	0.01	0.59	0.93	.
1943-1953; Age 70-79	0.77	0.12	0.10	0.57	1.05	.
1943 or Earlier; Age 80 or Older	0.85	0.24	0.57	0.48	1.50	.
Mother Absence/Presence (Ref: Present)						
Absent	1.43	0.31	0.10	0.93	2.20	0.10
Father Absence/Presence (Ref: Present)						
Absent	0.94	0.17	0.74	0.66	1.35	0.74
Childhood Religion (Ref: Judaism)						
Islam	1.63	0.79	0.31	0.63	4.25	0.02
Some Other Religion	0.62	0.28	0.28	0.25	1.50	.
Race/Ethnicity (Ref: Ethnic Plurality)						
Ethnic Minority	0.87	0.42	0.77	0.33	2.26	0.77

Table S9c: E-Values and E-Value Limits (Israel)

Variable	E-Value	E-Value Limit
Relationship with Mother (Ref: Very/Somewhat Bad)		
Very/Somewhat Good	1.23	1.00
Relationship with Father (Ref: Very/Somewhat Bad)		
Very/Somewhat Good	1.38	1.00
Parent Marital Status (Ref: Married)		
Divorced	2.04	1.13
Never Married	2.54	1.21
One or Both Had Died	2.73	1.44
Childhood Income (Ref: Got By)		
Lived Comfortably	1.41	1.00
Found it Difficult	1.67	1.19
Found it Very Difficult	1.40	1.00
Childhood Abuse (Ref: No)		
Yes	1.00	1.00
Outsider (Ref: No)		
Yes	1.35	1.00
Childhood Health (Ref: Good)		
Excellent	2.25	1.36
Very Good	1.96	1.23
Fair	1.92	1.00
Poor	8.13	2.96
Immigration Status (Ref: Born in This Country)		
Born in Another Country	2.05	1.32
Childhood Service Attendance (Ref: Never)		
At Least 1/Week	6.36	2.91
1-3/Month	4.78	2.42
<1/Month	2.39	1.45
Gender (Ref: Male)		
Female	1.50	1.04
Other	8818.34	67.16
Year of Birth (Ref: 1998-2005)		
1993-1998; Age 25-29	1.50	1.00
1983-1993; Age 30-39	1.73	1.17
1973-1983; Age 40-49	1.74	1.10
1963-1973; Age 50-59	1.36	1.00
1953-1963; Age 60-69	1.96	1.24
1943-1953; Age 70-79	1.85	1.00
1943 or Earlier; Age 80 or Older	1.60	1.00
Mother Absence/Presence (Ref: Present)		
Absent	2.13	1.00
Father Absence/Presence (Ref: Present)		
Absent	1.31	1.00
Childhood Religion (Ref: Judaism)		
Islam	2.52	1.00
Some Other Religion	2.50	1.00
Race/Ethnicity (Ref: Ethnic Plurality)		
Ethnic Minority	1.53	1.00

Table S10a: Nationally Representative Descriptive Statistics of the Observed Sample (Japan)

Variable	Proportion	Frequency
Relationship with Mother		
Very Good	0.27	5630
Somewhat Good	0.46	9461
Somewhat Bad	0.13	2750
Very Bad	0.04	799
Not Applicable	0.09	1838
Missing	0.00	66
Relationship with Father		
Very Good	0.20	4156
Somewhat Good	0.44	9081
Somewhat Bad	0.17	3446
Very Bad	0.06	1223
Not Applicable	0.13	2580
Missing	0.00	57
Parent Marital Status		
Married	0.86	17713
Divorced	0.05	1127
Never Married	0.03	591
One or Both Had Died	0.04	754
Missing	0.02	359
Childhood Income		
Lived Comfortably	0.41	8320
Got By	0.43	8799
Found it Difficult	0.12	2398
Found it Very Difficult	0.05	973
Missing	0.00	52
Childhood Abuse		
Yes	0.07	1482
No	0.92	18964
Missing	0.00	96
Outsider		
Yes	0.10	1963
No	0.83	17136
Not Applicable	0.07	1402
Missing	0.00	42
Childhood Health		
Excellent	0.13	2711
Very Good	0.35	7106
Good	0.33	6689
Fair	0.16	3199
Poor	0.04	758
Missing	0.00	80
Immigration Status		
Born in This Country	0.95	19548

Born in Another Country	0.01	158
Missing	0.04	837
Childhood Service Attendance		
At Least 1/Week	0.02	398
1-3/Month	0.04	883
<1/Month	0.24	5023
Never	0.69	14117
Missing	0.01	123
Gender		
Male	0.48	9847
Female	0.52	10602
Other	0.00	28
Missing	0.00	66
Year of Birth		
1998-2005; Age 18-24	0.08	1589
1993-1998; Age 25-29	0.04	806
1983-1993; Age 30-39	0.14	2851
1973-1983; Age 40-49	0.16	3363
1963-1973; Age 50-59	0.18	3770
1953-1963; Age 60-69	0.20	4118
1943-1953; Age 70-79	0.17	3554
1943 or Earlier; 80 or Older	0.02	493
Missing	.	.
Childhood Religion		
Christianity	0.02	343
Islam	0.00	7
Hinduism	0.00	4
Buddhism	0.32	6536
Judaism	.	.
Sikhism	.	.
Baha'i	0.00	7
Jainism	0.00	1
Shinto	0.02	382
Taoism	0.00	14
Confucianism	0.00	25
Primal, Animist, or Folk Religion	0.00	13
Spiritism	.	.
African-Derived	.	.
Chinese	.	.
Some Other Religion	0.00	46
No Religion/Atheist/Agnostic	0.63	12950
Missing	0.01	215
Race/Ethnicity		
No Data	.	.

Table S10b: Variations Across Childhood Predictors (Japan)

Variable	IRR	SE	Prob	LCI	UCI	Global p-value
Relationship with Mother (Ref: Very/Somewhat Bad)						
Very/Somewhat Good	1.02	0.12	0.88	0.80	1.30	0.88
Relationship with Father (Ref: Very/Somewhat Bad)						
Very/Somewhat Good	1.14	0.12	0.22	0.93	1.39	0.22
Parent Marital Status (Ref: Married)						
Divorced	1.64	0.25	0.00	1.21	2.22	0.00
Never Married	2.23	0.34	0.00	1.65	3.00	.
One or Both Had Died	2.31	0.48	0.00	1.53	3.48	.
Childhood Income (Ref: Got By)						
Lived Comfortably	0.79	0.07	0.01	0.65	0.94	0.00
Found it Difficult	1.05	0.13	0.71	0.82	1.35	.
Found it Very Difficult	0.52	0.12	0.01	0.33	0.82	.
Childhood Abuse (Ref: No)						
Yes	1.39	0.17	0.01	1.09	1.76	0.01
Outsider (Ref: No)						
Yes	2.05	0.24	0.00	1.63	2.57	0.00
Childhood Health (Ref: Good)						
Excellent	1.36	0.17	0.01	1.06	1.73	0.00
Very Good	1.34	0.14	0.00	1.10	1.65	.
Fair	0.79	0.12	0.11	0.59	1.06	.
Poor	1.11	0.24	0.65	0.72	1.70	.
Immigration Status (Ref: Born in This Country)						
Born in Another Country	1.12	0.41	0.76	0.55	2.28	0.76
Childhood Service Attendance (Ref: Never)						
At Least 1/Week	13.71	2.16	0.00	10.07	18.66	0.00
1-3/Month	12.02	1.71	0.00	9.10	15.88	.
<1/Month	2.86	0.39	0.00	2.20	3.73	.
Gender (Ref: Male)						
Female	0.82	0.07	0.02	0.70	0.97	0.07
Other	1.12	0.71	0.86	0.32	3.88	.
Year of Birth (Ref: 1998-2005)						
1993-1998; Age 25-29	1.07	0.21	0.74	0.72	1.58	0.05
1983-1993; Age 30-39	0.93	0.15	0.68	0.68	1.29	.
1973-1983; Age 40-49	0.84	0.14	0.30	0.60	1.17	.
1963-1973; Age 50-59	0.67	0.12	0.02	0.48	0.94	.
1953-1963; Age 60-69	0.79	0.14	0.17	0.56	1.11	.
1943-1953; Age 70-79	0.71	0.13	0.05	0.50	1.00	.
1943 or Earlier; Age 80 or Older	1.16	0.29	0.56	0.70	1.91	.
Mother Absence/Presence (Ref: Present)						
Absent	1.52	0.26	0.02	1.07	2.15	0.02
Father Absence/Presence (Ref: Present)						
Absent	0.57	0.09	0.00	0.42	0.78	0.00

Childhood Religion (Ref: No Religion/Atheist/Agnostic)						
Buddhism	1.71	0.19	0.00	1.38	2.12	0.00
Some Other Religion	2.22	0.29	0.00	1.72	2.87	.

Table S10c: E-Values and E-Value Limits (Japan)

Variable	E-Value	E-Value Limit
Relationship with Mother (Ref: Very/Somewhat Bad)		
Very/Somewhat Good	1.29	1.00
Relationship with Father (Ref: Very/Somewhat Bad)		
Very/Somewhat Good	2.16	1.00
Parent Marital Status (Ref: Married)		
Divorced	6.94	1.44
Never Married	16.34	1.89
One or Both Had Died	18.06	1.78
Childhood Income (Ref: Got By)		
Lived Comfortably	3.22	1.20
Found it Difficult	1.52	1.00
Found it Very Difficult	11.05	1.44
Childhood Abuse (Ref: No)		
Yes	4.22	1.27
Outsider (Ref: No)		
Yes	12.93	1.87
Childhood Health (Ref: Good)		
Excellent	3.93	1.21
Very Good	3.83	1.27
Fair	3.18	1.00
Poor	1.94	1.00
Immigration Status (Ref: Born in This Country)		
Born in Another Country	2.03	1.00
Childhood Service Attendance (Ref: Never)		
At Least 1/Week	2127.12	5.80
1-3/Month	1500.52	5.48
<1/Month	32.42	2.33
Gender (Ref: Male)		
Female	2.73	1.13
Other	2.03	1.00
Year of Birth (Ref: 1998-2005)		
1993-1998; Age 25-29	1.67	1.00
1983-1993; Age 30-39	1.69	1.00
1973-1983; Age 40-49	2.56	1.00
1963-1973; Age 50-59	5.27	1.21
1953-1963; Age 60-69	3.21	1.00
1943-1953; Age 70-79	4.41	1.00
1943 or Earlier; Age 80 or Older	2.33	1.00
Mother Absence/Presence (Ref: Present)		

Absent	5.50	1.22
Father Absence/Presence (Ref: Present)		
Absent	8.40	1.53
Childhood Religion (Ref: No Religion/Atheist/Agnostic)		
Buddhism	7.82	1.63
Some Other Religion	16.21	1.95

Table S11a: Nationally Representative Descriptive Statistics of the Observed Sample (Kenya)

Variable	Proportion	Frequency
Relationship with Mother		
Very Good	0.83	9418
Somewhat Good	0.13	1435
Somewhat Bad	0.01	130
Very Bad	0.01	100
Not Applicable	0.02	240
Missing	0.01	66
Relationship with Father		
Very Good	0.70	7958
Somewhat Good	0.17	1896
Somewhat Bad	0.02	216
Very Bad	0.02	220
Not Applicable	0.08	967
Missing	0.01	132
Parent Marital Status		
Married	0.81	9238
Divorced	0.06	697
Never Married	0.06	681
One or Both Had Died	0.04	471
Missing	0.03	301
Childhood Income		
Lived Comfortably	0.27	3026
Got By	0.29	3279
Found it Difficult	0.36	4071
Found it Very Difficult	0.09	994
Missing	0.00	19
Childhood Abuse		
Yes	0.11	1300
No	0.88	10040
Missing	0.00	49
Outsider		
Yes	0.11	1223
No	0.89	10114
Not Applicable	0.00	23
Missing	0.00	29
Childhood Health		
Excellent	0.39	4449
Very Good	0.23	2598
Good	0.23	2582
Fair	0.12	1384

Poor	0.03	349
Missing	0.00	26
Immigration Status		
Born in This Country	0.99	11270
Born in Another Country	0.01	117
Missing	0.00	2
Childhood Service Attendance		
At Least 1/Week	0.81	9189
1-3/Month	0.15	1687
<1/Month	0.02	236
Never	0.02	198
Missing	0.01	79
Gender		
Male	0.49	5567
Female	0.51	5813
Other	0.00	2
Missing	0.00	7
Year of Birth		
1998-2005; Age 18-24	0.25	2868
1993-1998; Age 25-29	0.18	2035
1983-1993; Age 30-39	0.23	2564
1973-1983; Age 40-49	0.15	1708
1963-1973; Age 50-59	0.09	1072
1953-1963; Age 60-69	0.06	710
1943-1953; Age 70-79	0.03	360
1943 or Earlier; 80 or Older	0.01	67
Missing	0.00	5
Childhood Religion		
Christianity	0.91	10369
Islam	0.08	916
Hinduism	.	.
Buddhism	0.00	5
Judaism	0.00	6
Sikhism	0.00	0
Baha'i	0.00	3
Jainism	0.00	1
Shinto	.	.
Taoism	.	.
Confucianism	.	.
Primal, Animist, or Folk Religion	0.00	13
Spiritism	.	.
African-Derived	.	.
Chinese	.	.
Some Other Religion	0.00	0
No Religion/Atheist/Agnostic	0.01	67
Missing	0.00	9
Race/Ethnicity		
Luhya	0.17	1943
Luo	0.10	1120

Kalenjin	0.12	1377
Kamba	0.11	1299
Kikuyu	0.19	2118
Kisii	0.07	789
Maasai	0.02	237
Meru	0.06	630
Kenan Somali/Somali	0.03	396
Miji Kenda Tribes	0.06	708
Embu	0.02	197
Other	0.05	548
Missing	0.00	27

Table S11b: Variations Across Childhood Predictors (Kenya)

Variable	IRR	SE	Prob	LCI	UCI	Global p-value
Relationship with Mother (Ref: Very/Somewhat Bad)						
Very/Somewhat Good	1.02	0.05	0.70	0.92	1.13	0.70
Relationship with Father (Ref: Very/Somewhat Bad)						
Very/Somewhat Good	1.00	0.04	0.92	0.92	1.07	0.92
Parent Marital Status (Ref: Married)						
Divorced	1.02	0.03	0.61	0.96	1.08	0.00
Never Married	0.86	0.04	0.00	0.79	0.93	.
One or Both Had Died	0.88	0.05	0.01	0.79	0.97	.
Childhood Income (Ref: Got By)						
Lived Comfortably	0.97	0.02	0.20	0.93	1.02	0.01
Found it Difficult	1.01	0.02	0.62	0.97	1.05	.
Found it Very Difficult	1.08	0.03	0.01	1.02	1.14	.
Childhood Abuse (Ref: No)						
Yes	0.97	0.03	0.29	0.92	1.03	0.29
Outsider (Ref: No)						
Yes	0.96	0.03	0.15	0.91	1.02	0.15
Childhood Health (Ref: Good)						
Excellent	1.11	0.03	0.00	1.06	1.16	0.00
Very Good	1.03	0.03	0.31	0.98	1.08	.
Fair	1.03	0.03	0.30	0.97	1.09	.
Poor	1.02	0.06	0.68	0.92	1.14	.
Immigration Status (Ref: Born in This Country)						
Born in Another Country	1.04	0.06	0.51	0.92	1.17	0.51
Childhood Service Attendance (Ref: Never)						
At Least 1/Week	1.12	0.08	0.12	0.97	1.30	0.05
1-3/Month	1.06	0.09	0.44	0.91	1.25	.
<1/Month	1.15	0.11	0.15	0.95	1.40	.
Gender (Ref: Male)						

Female	1.15	0.02	0.00	1.11	1.19	0.00
Other	1.94	0.15	0.00	1.66	2.26	.
Year of Birth (Ref: 1998-2005)						
1993-1998; Age 25-29	1.05	0.03	0.05	1.00	1.10	0.00
1983-1993; Age 30-39	1.20	0.03	0.00	1.15	1.25	.
1973-1983; Age 40-49	1.29	0.03	0.00	1.23	1.36	.
1963-1973; Age 50-59	1.32	0.05	0.00	1.23	1.42	.
1953-1963; Age 60-69	1.35	0.05	0.00	1.25	1.45	.
1943-1953; Age 70-79	1.32	0.08	0.00	1.17	1.48	.
1943 or Earlier; Age 80 or Older	1.21	0.20	0.26	0.87	1.67	.
Mother Absence/Presence (Ref: Present)						
Absent	1.10	0.07	0.12	0.97	1.24	0.12
Father Absence/Presence (Ref: Present)						
Absent	0.99	0.04	0.78	0.91	1.07	0.78
Childhood Religion (Ref: Christianity)						
Islam	0.95	0.04	0.22	0.87	1.03	0.16
Some Other Religion	0.82	0.10	0.12	0.64	1.05	.
Race/Ethnicity (Ref: Ethnic Plurality)						
Ethnic Minority	1.21	0.04	0.00	1.13	1.29	0.00

Table S11c: E-Values and E-Value Limits (Kenya)

Variable	E-Value	E-Value Limit
Relationship with Mother (Ref: Very/Somewhat Bad)		
Very/Somewhat Good	1.13	1.00
Relationship with Father (Ref: Very/Somewhat Bad)		
Very/Somewhat Good	1.05	1.00
Parent Marital Status (Ref: Married)		
Divorced	1.11	1.00
Never Married	1.43	1.23
One or Both Had Died	1.39	1.13
Childhood Income (Ref: Got By)		
Lived Comfortably	1.15	1.00
Found it Difficult	1.09	1.00
Found it Very Difficult	1.27	1.10
Childhood Abuse (Ref: No)		
Yes	1.16	1.00
Outsider (Ref: No)		
Yes	1.19	1.00
Childhood Health (Ref: Good)		
Excellent	1.33	1.20
Very Good	1.14	1.00
Fair	1.16	1.00
Poor	1.14	1.00
Immigration Status (Ref: Born in This Country)		
Born in Another Country	1.19	1.00
Childhood Service Attendance (Ref: Never)		

At Least 1/Week	1.36	1.00
1-3/Month	1.24	1.00
<1/Month	1.41	1.00
Gender (Ref: Male)		
Female	1.41	1.29
Other	2.39	1.90
Year of Birth (Ref: 1998-2005)		
1993-1998; Age 25-29	1.21	1.02
1983-1993; Age 30-39	1.48	1.35
1973-1983; Age 40-49	1.62	1.45
1963-1973; Age 50-59	1.67	1.46
1953-1963; Age 60-69	1.70	1.49
1943-1953; Age 70-79	1.66	1.38
1943 or Earlier; Age 80 or Older	1.50	1.00
Mother Absence/Presence (Ref: Present)		
Absent	1.32	1.00
Father Absence/Presence (Ref: Present)		
Absent	1.09	1.00
Childhood Religion (Ref: Christianity)		
Islam	1.22	1.00
Some Other Religion	1.52	1.00
Race/Ethnicity (Ref: Ethnic Plurality)		
Ethnic Minority	1.50	1.33

Table S12a: Nationally Representative Descriptive Statistics of the Observed Sample (Mexico)

Variable	Proportion	Frequency
Relationship with Mother		
Very Good	0.68	3912
Somewhat Good	0.23	1340
Somewhat Bad	0.03	177
Very Bad	0.02	90
Not Applicable	0.03	177
Missing	0.01	80
Relationship with Father		
Very Good	0.53	3089
Somewhat Good	0.27	1556
Somewhat Bad	0.06	335
Very Bad	0.05	267
Not Applicable	0.08	470
Missing	0.01	60
Parent Marital Status		
Married	0.69	3999
Divorced	0.06	341
Never Married	0.14	827
One or Both Had Died	0.03	176
Missing	0.07	432
Childhood Income		

Lived Comfortably	0.31	1775
Got By	0.32	1872
Found it Difficult	0.30	1712
Found it Very Difficult	0.06	369
Missing	0.01	48
Childhood Abuse		
Yes	0.16	905
No	0.80	4604
Missing	0.05	267
Outsider		
Yes	0.13	772
No	0.85	4897
Not Applicable	0.01	58
Missing	0.01	49
Childhood Health		
Excellent	0.32	1860
Very Good	0.23	1350
Good	0.29	1677
Fair	0.13	743
Poor	0.02	133
Missing	0.00	14
Immigration Status		
Born in This Country	0.96	5517
Born in Another Country	0.02	108
Missing	0.03	151
Childhood Service Attendance		
At Least 1/Week	0.44	2514
1-3/Month	0.20	1162
<1/Month	0.19	1087
Never	0.16	944
Missing	0.01	69
Gender		
Male	0.48	2755
Female	0.52	2997
Other	0.00	3
Missing	0.00	21
Year of Birth		
1998-2005; Age 18-24	0.17	986
1993-1998; Age 25-29	0.11	623
1983-1993; Age 30-39	0.23	1312
1973-1983; Age 40-49	0.18	1027
1963-1973; Age 50-59	0.15	873
1953-1963; Age 60-69	0.11	611
1943-1953; Age 70-79	0.05	277
1943 or Earlier; 80 or Older	0.01	68
Missing	.	.
Childhood Religion		
Christianity	0.92	5337
Islam	0.00	6

Hinduism	0.00	1
Buddhism	0.00	1
Judaism	0.00	8
Sikhism	0.00	4
Baha'i	0.00	1
Jainism	.	.
Shinto	0.00	2
Taoism	0.00	5
Confucianism	.	.
Primal, Animist, or Folk Religion	0.00	2
Spiritism	.	.
African-Derived	.	.
Chinese	.	.
Some Other Religion	0.00	7
No Religion/Atheist/Agnostic	0.06	328
Missing	0.01	74
Race/Ethnicity		
White	0.19	1116
Mestizo	0.48	2762
Indigenous	0.10	594
Black	0.02	108
Mulatto	0.01	63
Other	0.06	339
Missing	0.14	794

Table S12b: Variations Across Childhood Predictors (Mexico)

Variable	IRR	SE	Prob	LCI	UCI	Global p-value
Relationship with Mother (Ref: Very/Somewhat Bad)						
Very/Somewhat Good	0.91	0.11	0.45	0.72	1.16	0.45
Relationship with Father (Ref: Very/Somewhat Bad)						
Very/Somewhat Good	1.08	0.11	0.41	0.90	1.31	0.41
Parent Marital Status (Ref: Married)						
Divorced	1.23	0.15	0.09	0.97	1.55	0.16
Never Married	0.93	0.10	0.50	0.74	1.16	.
One or Both Had Died	0.84	0.16	0.36	0.57	1.23	.
Childhood Income (Ref: Got By)						
Lived Comfortably	1.19	0.09	0.03	1.02	1.38	0.02
Found it Difficult	1.13	0.09	0.14	0.96	1.32	.
Found it Very Difficult	1.41	0.17	0.00	1.12	1.78	.
Childhood Abuse (Ref: No)						
Yes	1.04	0.09	0.68	0.88	1.22	0.68
Outsider (Ref: No)						
Yes	1.25	0.11	0.01	1.06	1.48	0.01
Childhood Health (Ref: Good)						

Excellent	1.09	0.09	0.27	0.93	1.27	0.51
Very Good	0.96	0.09	0.67	0.80	1.15	.
Fair	0.92	0.10	0.44	0.75	1.13	.
Poor	1.02	0.18	0.92	0.72	1.43	.
Immigration Status (Ref: Born in This Country)						
Born in Another Country	0.98	0.21	0.92	0.64	1.50	0.92
Childhood Service Attendance (Ref: Never)						
At Least 1/Week	2.69	0.32	0.00	2.13	3.39	0.00
1-3/Month	2.17	0.28	0.00	1.68	2.79	.
<1/Month	1.23	0.18	0.16	0.92	1.64	.
Gender (Ref: Male)						
Female	1.05	0.07	0.41	0.93	1.19	0.30
Other	0.38	0.29	0.20	0.09	1.69	.
Year of Birth (Ref: 1998-2005)						
1993-1998; Age 25-29	1.14	0.15	0.33	0.88	1.47	0.00
1983-1993; Age 30-39	1.51	0.16	0.00	1.22	1.87	.
1973-1983; Age 40-49	1.58	0.18	0.00	1.26	1.98	.
1963-1973; Age 50-59	1.85	0.22	0.00	1.47	2.34	.
1953-1963; Age 60-69	1.82	0.23	0.00	1.42	2.34	.
1943-1953; Age 70-79	2.13	0.32	0.00	1.60	2.85	.
1943 or Earlier; Age 80 or Older	2.20	0.53	0.00	1.37	3.53	.
Mother Absence/Presence (Ref: Present)						
Absent	1.13	0.16	0.39	0.85	1.50	0.39
Father Absence/Presence (Ref: Present)						
Absent	0.97	0.11	0.81	0.78	1.22	0.81
Childhood Religion (Ref: No Religion/Atheist/Agnostic)						
Christianity	0.81	0.11	0.14	0.62	1.07	0.01
Some Other Religion	1.41	0.34	0.16	0.87	2.26	.
Race/Ethnicity (Ref: Ethnic Plurality)						
Ethnic Minority	1.16	0.08	0.02	1.02	1.32	0.02

Table S12c: E-Values and E-Value Limits (Mexico)

Variable	E-Value	E-Value Limit
Relationship with Mother (Ref: Very/Somewhat Bad)		
Very/Somewhat Good	1.43	1.00
Relationship with Father (Ref: Very/Somewhat Bad)		
Very/Somewhat Good	1.40	1.00
Parent Marital Status (Ref: Married)		
Divorced	1.78	1.00
Never Married	1.39	1.00
One or Both Had Died	1.71	1.00
Childhood Income (Ref: Got By)		
Lived Comfortably	1.68	1.10
Found it Difficult	1.52	1.00

Found it Very Difficult	2.22	1.31
Childhood Abuse (Ref: No)		
Yes	1.23	1.00
Outsider (Ref: No)		
Yes	1.83	1.20
Childhood Health (Ref: Good)		
Excellent	1.41	1.00
Very Good	1.25	1.00
Fair	1.40	1.00
Poor	1.15	1.00
Immigration Status (Ref: Born in This Country)		
Born in Another Country	1.18	1.00
Childhood Service Attendance (Ref: Never)		
At Least 1/Week	5.06	2.28
1-3/Month	3.91	1.92
<1/Month	1.79	1.00
Gender (Ref: Male)		
Female	1.30	1.00
Other	4.92	1.00
Year of Birth (Ref: 1998-2005)		
1993-1998; Age 25-29	1.55	1.00
1983-1993; Age 30-39	2.44	1.44
1973-1983; Age 40-49	2.60	1.49
1963-1973; Age 50-59	3.21	1.72
1953-1963; Age 60-69	3.14	1.66
1943-1953; Age 70-79	3.84	1.84
1943 or Earlier; Age 80 or Older	3.99	1.62
Mother Absence/Presence (Ref: Present)		
Absent	1.53	1.00
Father Absence/Presence (Ref: Present)		
Absent	1.20	1.00
Childhood Religion (Ref: No Religion/Atheist/Agnostic)		
Christianity	1.79	1.00
Some Other Religion	2.21	1.00
Race/Ethnicity (Ref: Ethnic Plurality)		
Ethnic Minority	1.62	1.12

Table S13a: Nationally Representative Descriptive Statistics of the Observed Sample (Nigeria)

Variable	Proportion	Frequency
Relationship with Mother		
Very Good	0.88	5986
Somewhat Good	0.09	648
Somewhat Bad	0.01	62
Very Bad	0.00	18
Not Applicable	0.02	104
Missing	0.00	9
Relationship with Father		

Very Good	0.82	5578
Somewhat Good	0.14	924
Somewhat Bad	0.01	76
Very Bad	0.01	43
Not Applicable	0.03	177
Missing	0.00	29
Parent Marital Status		
Married	0.82	5568
Divorced	0.05	307
Never Married	0.05	335
One or Both Had Died	0.07	462
Missing	0.02	154
Childhood Income		
Lived Comfortably	0.32	2192
Got By	0.35	2381
Found it Difficult	0.24	1661
Found it Very Difficult	0.08	563
Missing	0.00	29
Childhood Abuse		
Yes	0.13	880
No	0.86	5851
Missing	0.01	96
Outsider		
Yes	0.10	669
No	0.89	6059
Not Applicable	0.01	86
Missing	0.00	13
Childhood Health		
Excellent	0.39	2644
Very Good	0.38	2613
Good	0.17	1152
Fair	0.04	306
Poor	0.01	98
Missing	0.00	14
Immigration Status		
Born in This Country	0.99	6779
Born in Another Country	0.01	47
Missing	0.00	1
Childhood Service Attendance		
At Least 1/Week	0.87	5907
1-3/Month	0.09	600
<1/Month	0.02	136
Never	0.02	138
Missing	0.01	45
Gender		
Male	0.49	3371
Female	0.51	3456
Other	0.00	0
Missing	.	.

Year of Birth		
1998-2005; Age 18-24	0.22	1533
1993-1998; Age 25-29	0.17	1193
1983-1993; Age 30-39	0.28	1943
1973-1983; Age 40-49	0.16	1059
1963-1973; Age 50-59	0.09	619
1953-1963; Age 60-69	0.04	296
1943-1953; Age 70-79	0.02	133
1943 or Earlier; 80 or Older	0.01	50
Missing	.	.
Childhood Religion		
Christianity	0.51	3463
Islam	0.49	3314
Hinduism	.	.
Buddhism	0.00	0
Judaism	.	.
Sikhism	.	.
Baha'i	.	.
Jainism	.	.
Shinto	.	.
Taoism	.	.
Confucianism	0.00	0
Primal, Animist, or Folk Religion	0.00	17
Spiritism	.	.
African-Derived	.	.
Chinese	.	.
Some Other Religion	.	.
No Religion/Atheist/Agnostic	0.00	19
Missing	0.00	14
Race/Ethnicity		
Hausa	0.34	2342
Yoruba	0.18	1230
Igbo (Ibo)	0.16	1112
Edo	0.02	116
Urhobo	0.01	38
Fulani	0.04	266
Kanuri	0.00	31
Tiv	0.03	198
Efik	0.01	48
Ijaw	0.02	110
Igala	0.01	77
Ibibio	0.03	180
Idoma	0.01	61
Other	0.15	1014
Missing	0.00	4

Table S13b: Variations Across Childhood Predictors (Nigeria)

Variable	IRR	SE	Prob	LCI	UCI	Global p-value
Relationship with Mother (Ref: Very/Somewhat Bad)						
Very/Somewhat Good	1.27	0.16	0.07	0.98	1.64	0.07
Relationship with Father (Ref: Very/Somewhat Bad)						
Very/Somewhat Good	1.05	0.09	0.58	0.88	1.25	0.58
Parent Marital Status (Ref: Married)						
Divorced	1.05	0.07	0.48	0.92	1.20	0.85
Never Married	1.03	0.06	0.55	0.93	1.15	.
One or Both Had Died	0.99	0.06	0.84	0.88	1.11	.
Childhood Income (Ref: Got By)						
Lived Comfortably	1.02	0.03	0.45	0.96	1.09	0.79
Found it Difficult	0.99	0.04	0.71	0.92	1.06	.
Found it Very Difficult	1.00	0.05	0.93	0.91	1.11	.
Childhood Abuse (Ref: No)						
Yes	0.94	0.04	0.15	0.87	1.02	0.15
Outsider (Ref: No)						
Yes	1.09	0.04	0.03	1.01	1.17	0.03
Childhood Health (Ref: Good)						
Excellent	1.00	0.04	0.91	0.93	1.07	0.35
Very Good	1.03	0.04	0.44	0.96	1.11	.
Fair	0.97	0.08	0.76	0.83	1.15	.
Poor	1.16	0.10	0.09	0.98	1.37	.
Immigration Status (Ref: Born in This Country)						
Born in Another Country	0.62	0.17	0.08	0.36	1.06	0.08
Childhood Service Attendance (Ref: Never)						
At Least 1/Week	1.02	0.09	0.80	0.86	1.21	0.18
1-3/Month	1.00	0.09	0.98	0.83	1.21	.
<1/Month	0.82	0.10	0.10	0.64	1.04	.
Gender (Ref: Male)						
Female	1.05	0.03	0.07	1.00	1.11	0.00
Other	1.95	0.10	0.00	1.77	2.14	.
Year of Birth (Ref: 1998-2005)						
1993-1998; Age 25-29	1.06	0.03	0.07	1.00	1.13	0.00
1983-1993; Age 30-39	1.04	0.03	0.23	0.98	1.10	.
1973-1983; Age 40-49	1.16	0.05	0.00	1.07	1.26	.
1963-1973; Age 50-59	1.19	0.06	0.00	1.08	1.31	.
1953-1963; Age 60-69	1.18	0.11	0.07	0.99	1.41	.
1943-1953; Age 70-79	1.18	0.17	0.24	0.89	1.56	.
1943 or Earlier; Age 80 or Older	0.81	0.36	0.63	0.33	1.95	.
Mother Absence/Presence (Ref: Present)						
Absent	0.98	0.11	0.87	0.80	1.21	0.87
Father Absence/Presence (Ref: Present)						
Absent	0.94	0.08	0.52	0.79	1.12	0.52
Childhood Religion (Ref: Christianity)						

Islam	1.21	0.04	0.00	1.13	1.30	0.00
Some Other Religion	0.68	0.29	0.37	0.29	1.58	.
Race/Ethnicity (Ref: Ethnic Plurality)						
Ethnic Minority	0.95	0.03	0.17	0.89	1.02	0.17

Table S13c: E-Values and E-Value Limits (Nigeria)

Variable	E-Value	E-Value Limit
Relationship with Mother (Ref: Very/Somewhat Bad)		
Very/Somewhat Good	1.60	1.00
Relationship with Father (Ref: Very/Somewhat Bad)		
Very/Somewhat Good	1.21	1.00
Parent Marital Status (Ref: Married)		
Divorced	1.21	1.00
Never Married	1.17	1.00
One or Both Had Died	1.09	1.00
Childhood Income (Ref: Got By)		
Lived Comfortably	1.14	1.00
Found it Difficult	1.10	1.00
Found it Very Difficult	1.06	1.00
Childhood Abuse (Ref: No)		
Yes	1.24	1.00
Outsider (Ref: No)		
Yes	1.29	1.06
Childhood Health (Ref: Good)		
Excellent	1.05	1.00
Very Good	1.15	1.00
Fair	1.14	1.00
Poor	1.42	1.00
Immigration Status (Ref: Born in This Country)		
Born in Another Country	2.04	1.00
Childhood Service Attendance (Ref: Never)		
At Least 1/Week	1.13	1.00
1-3/Month	1.04	1.00
<1/Month	1.53	1.00
Gender (Ref: Male)		
Female	1.22	1.00
Other	2.41	1.99
Year of Birth (Ref: 1998-2005)		
1993-1998; Age 25-29	1.24	1.00
1983-1993; Age 30-39	1.18	1.00
1973-1983; Age 40-49	1.43	1.23
1963-1973; Age 50-59	1.47	1.23
1953-1963; Age 60-69	1.46	1.00
1943-1953; Age 70-79	1.46	1.00
1943 or Earlier; Age 80 or Older	1.55	1.00
Mother Absence/Presence (Ref: Present)		

Absent	1.12	1.00
Father Absence/Presence (Ref: Present)		
Absent	1.23	1.00
Childhood Religion (Ref: Christianity)		
Islam	1.51	1.32
Some Other Religion	1.86	1.00
Race/Ethnicity (Ref: Ethnic Plurality)		
Ethnic Minority	1.20	1.00

Table S14a: Nationally Representative Descriptive Statistics of the Observed Sample (Philippines)

Variable	Proportion	Frequency
Relationship with Mother		
Very Good	0.63	3333
Somewhat Good	0.32	1703
Somewhat Bad	0.02	124
Very Bad	0.01	39
Not Applicable	0.01	59
Missing	0.01	35
Relationship with Father		
Very Good	0.65	3443
Somewhat Good	0.27	1429
Somewhat Bad	0.03	159
Very Bad	0.01	58
Not Applicable	0.02	108
Missing	0.02	95
Parent Marital Status		
Married	0.86	4575
Divorced	0.01	64
Never Married	0.10	517
One or Both Had Died	0.01	51
Missing	0.02	86
Childhood Income		
Lived Comfortably	0.18	937
Got By	0.57	3006
Found it Difficult	0.20	1055
Found it Very Difficult	0.06	291
Missing	0.00	3
Childhood Abuse		
Yes	0.08	420
No	0.91	4837
Missing	0.01	35
Outsider		
Yes	0.07	395
No	0.92	4884
Not Applicable	0.00	3
Missing	0.00	9

Childhood Health		
Excellent	0.20	1041
Very Good	0.11	559
Good	0.41	2174
Fair	0.24	1246
Poor	0.05	272
Missing	0.00	0
Immigration Status		
Born in This Country	1.00	5284
Born in Another Country	0.00	8
Missing	.	.
Childhood Service Attendance		
At Least 1/Week	0.46	2453
1-3/Month	0.32	1699
<1/Month	0.17	892
Never	0.04	201
Missing	0.01	47
Gender		
Male	0.50	2625
Female	0.50	2643
Other	0.00	13
Missing	0.00	11
Year of Birth		
1998-2005; Age 18-24	0.20	1073
1993-1998; Age 25-29	0.13	695
1983-1993; Age 30-39	0.22	1160
1973-1983; Age 40-49	0.18	972
1963-1973; Age 50-59	0.14	732
1953-1963; Age 60-69	0.09	495
1943-1953; Age 70-79	0.03	143
1943 or Earlier; 80 or Older	0.00	23
Missing	.	.
Childhood Religion		
Christianity	0.94	4968
Islam	0.05	276
Hinduism	.	.
Buddhism	0.00	1
Judaism	.	.
Sikhism	0.00	4
Baha'i	0.00	1
Jainism	.	.
Shinto	.	.
Taoism	.	.
Confucianism	.	.
Primal, Animist, or Folk Religion	0.00	14
Spiritism	.	.
African-Derived	.	.
Chinese	.	.
Some Other Religion	0.00	9

No Religion/Atheist/Agnostic	0.00	9
Missing	0.00	11
Race/Ethnicity		
Tagalog	0.32	1691
Cebuana	0.12	656
Ilocano/Ilokano	0.08	429
Visayan/Bisaya	0.14	739
Ilonggo/Hiligaynon	0.08	428
Bicolano/Bikolano	0.06	300
Waray	0.04	216
Tausug	0.02	94
Maranao	0.01	39
Maguindanaoan	0.02	84
Chinese-Filipino	0.00	3
Kapampangan	0.02	107
Pangasinese	0.02	107
Zamboangueno	0.01	51
Malay	.	.
Masbateno	0.01	54
Aeta	0.00	1
Igorot	0.01	42
Mangyan	0.00	2
Badjao	0.00	2
Other	0.05	244
Missing	0.00	3

Table S14b: Variations Across Childhood Predictors (Philippines)

Variable	IRR	SE	Prob	LCI	UCI	Global p-value
Relationship with Mother (Ref: Very/Somewhat Bad)						
Very/Somewhat Good	1.03	0.17	0.84	0.74	1.44	0.84
Relationship with Father (Ref: Very/Somewhat Bad)						
Very/Somewhat Good	1.02	0.14	0.87	0.78	1.33	0.87
Parent Marital Status (Ref: Married)						
Divorced	0.89	0.24	0.68	0.52	1.53	0.54
Never Married	0.86	0.09	0.16	0.70	1.06	.
One or Both Had Died	0.90	0.25	0.71	0.52	1.56	.
Childhood Income (Ref: Got By)						
Lived Comfortably	1.16	0.09	0.06	0.99	1.35	0.26
Found it Difficult	1.05	0.07	0.52	0.91	1.20	.
Found it Very Difficult	0.96	0.12	0.74	0.76	1.22	.
Childhood Abuse (Ref: No)						
Yes	1.08	0.12	0.50	0.87	1.34	0.50
Outsider (Ref: No)						
Yes	0.78	0.09	0.02	0.62	0.97	0.02

Childhood Health (Ref: Good)						
Excellent	1.04	0.08	0.64	0.89	1.21	0.19
Very Good	1.15	0.09	0.09	0.98	1.35	.
Fair	0.95	0.07	0.54	0.82	1.11	.
Poor	1.18	0.15	0.19	0.92	1.50	.
Immigration Status (Ref: Born in This Country)						
Born in Another Country	1.12	0.62	0.84	0.38	3.34	0.84
Childhood Service Attendance (Ref: Never)						
At Least 1/Week	1.76	0.36	0.01	1.17	2.63	0.00
1-3/Month	1.23	0.25	0.32	0.82	1.85	.
<1/Month	1.10	0.23	0.64	0.73	1.66	.
Gender (Ref: Male)						
Female	1.08	0.07	0.20	0.96	1.22	0.04
Other	1.86	0.50	0.02	1.10	3.17	.
Year of Birth (Ref: 1998-2005)						
1993-1998; Age 25-29	0.99	0.11	0.91	0.79	1.23	0.06
1983-1993; Age 30-39	0.84	0.08	0.06	0.70	1.01	.
1973-1983; Age 40-49	0.95	0.09	0.57	0.79	1.14	.
1963-1973; Age 50-59	1.10	0.11	0.32	0.91	1.34	.
1953-1963; Age 60-69	1.06	0.14	0.67	0.82	1.37	.
1943-1953; Age 70-79	1.09	0.20	0.65	0.76	1.56	.
1943 or Earlier; Age 80 or Older	1.42	0.47	0.29	0.74	2.71	.
Mother Absence/Presence (Ref: Present)						
Absent	1.22	0.23	0.28	0.85	1.76	0.28
Father Absence/Presence (Ref: Present)						
Absent	0.99	0.15	0.97	0.74	1.34	0.97
Childhood Religion (Ref: Christianity)						
Islam	1.63	0.16	0.00	1.35	1.97	0.00
Some Other Religion	1.56	0.49	0.16	0.84	2.88	.
Race/Ethnicity (Ref: Ethnic Plurality)						
Ethnic Minority	1.12	0.08	0.11	0.98	1.28	0.11

Table S14c: E-Values and E-Value Limits (Philippines)

Variable	E-Value	E-Value Limit
Relationship with Mother (Ref: Very/Somewhat Bad)		
Very/Somewhat Good	1.22	1.00
Relationship with Father (Ref: Very/Somewhat Bad)		
Very/Somewhat Good	1.17	1.00
Parent Marital Status (Ref: Married)		
Divorced	1.48	1.00
Never Married	1.59	1.00
One or Both Had Died	1.46	1.00
Childhood Income (Ref: Got By)		
Lived Comfortably	1.57	1.00

Found it Difficult	1.27	1.00
Found it Very Difficult	1.25	1.00
Childhood Abuse (Ref: No)		
Yes	1.37	1.00
Outsider (Ref: No)		
Yes	1.88	1.15
Childhood Health (Ref: Good)		
Excellent	1.23	1.00
Very Good	1.56	1.00
Fair	1.27	1.00
Poor	1.62	1.00
Immigration Status (Ref: Born in This Country)		
Born in Another Country	1.48	1.00
Childhood Service Attendance (Ref: Never)		
At Least 1/Week	2.89	1.38
1-3/Month	1.75	1.00
<1/Month	1.44	1.00
Gender (Ref: Male)		
Female	1.38	1.00
Other	3.10	1.27
Year of Birth (Ref: 1998-2005)		
1993-1998; Age 25-29	1.13	1.00
1983-1993; Age 30-39	1.66	1.00
1973-1983; Age 40-49	1.29	1.00
1963-1973; Age 50-59	1.44	1.00
1953-1963; Age 60-69	1.30	1.00
1943-1953; Age 70-79	1.39	1.00
1943 or Earlier; Age 80 or Older	2.18	1.00
Mother Absence/Presence (Ref: Present)		
Absent	1.74	1.00
Father Absence/Presence (Ref: Present)		
Absent	1.08	1.00
Childhood Religion (Ref: Christianity)		
Islam	2.63	1.60
Some Other Religion	2.47	1.00
Race/Ethnicity (Ref: Ethnic Plurality)		
Ethnic Minority	1.47	1.00

Table S15a: Nationally Representative Descriptive Statistics of the Observed Sample (Poland)

Variable	Proportion	Frequency
Relationship with Mother		
Very Good	0.47	4879
Somewhat Good	0.48	4973
Somewhat Bad	0.03	285
Very Bad	0.01	58
Not Applicable	0.01	80
Missing	0.01	112

Relationship with Father		
Very Good	0.41	4231
Somewhat Good	0.48	4984
Somewhat Bad	0.05	516
Very Bad	0.01	78
Not Applicable	0.04	407
Missing	0.02	173
Parent Marital Status		
Married	0.86	8972
Divorced	0.06	587
Never Married	0.02	193
One or Both Had Died	0.03	313
Missing	0.03	324
Childhood Income		
Lived Comfortably	0.13	1384
Got By	0.60	6257
Found it Difficult	0.21	2133
Found it Very Difficult	0.05	509
Missing	0.01	106
Childhood Abuse		
Yes	0.03	325
No	0.96	10009
Missing	0.01	55
Outsider		
Yes	0.05	490
No	0.93	9615
Not Applicable	0.00	33
Missing	0.02	252
Childhood Health		
Excellent	0.26	2676
Very Good	0.52	5371
Good	0.17	1779
Fair	0.04	406
Poor	0.01	123
Missing	0.00	34
Immigration Status		
Born in This Country	0.99	10258
Born in Another Country	0.01	108
Missing	0.00	23
Childhood Service Attendance		
At Least 1/Week	0.46	4751
1-3/Month	0.26	2689
<1/Month	0.21	2161
Never	0.03	354
Missing	0.04	434
Gender		
Male	0.48	4974
Female	0.52	5387
Other	0.00	3

Missing	0.00	26
Year of Birth		
1998-2005; Age 18-24	0.09	955
1993-1998; Age 25-29	0.07	761
1983-1993; Age 30-39	0.21	2159
1973-1983; Age 40-49	0.19	1956
1963-1973; Age 50-59	0.16	1670
1953-1963; Age 60-69	0.18	1909
1943-1953; Age 70-79	0.08	833
1943 or Earlier; 80 or Older	0.01	145
Missing	0.00	1
Childhood Religion		
Christianity	0.95	9861
Islam	0.00	3
Hinduism	.	.
Buddhism	0.00	2
Judaism	.	.
Sikhism	0.00	1
Baha'i	.	.
Jainism	.	.
Shinto	.	.
Taoism	.	.
Confucianism	.	.
Primal, Animist, or Folk Religion	0.00	5
Spiritism	.	.
African-Derived	.	.
Chinese	.	.
Some Other Religion	.	.
No Religion/Atheist/Agnostic	0.05	482
Missing	0.00	35
Race/Ethnicity		
Polish	0.99	10309
German	0.00	4
Belarussian	0.00	2
Ukranian	0.00	38
Roma	.	.
Russian	.	.
Ethnic Jewish	.	.
Lemko	.	.
Silesia	0.00	14
Kashubians	0.00	3
Other	0.00	4
Missing	0.00	14

Table S15b: Variations Across Childhood Predictors (Poland)

Variable	IRR	SE	Prob	LCI	UCI	Global p-value

Relationship with Mother (Ref: Very/Somewhat Bad)						
Very/Somewhat Good	1.10	0.31	0.74	0.63	1.91	0.74
Relationship with Father (Ref: Very/Somewhat Bad)						
Very/Somewhat Good	1.25	0.29	0.34	0.79	1.97	0.34
Parent Marital Status (Ref: Married)						
Divorced	1.80	0.36	0.00	1.21	2.68	0.00
Never Married	3.14	0.78	0.00	1.92	5.12	.
One or Both Had Died	1.29	0.43	0.44	0.67	2.47	.
Childhood Income (Ref: Got By)						
Lived Comfortably	1.13	0.22	0.53	0.77	1.65	0.59
Found it Difficult	1.17	0.17	0.27	0.88	1.56	.
Found it Very Difficult	1.30	0.31	0.26	0.82	2.08	.
Childhood Abuse (Ref: No)						
Yes	0.90	0.23	0.69	0.55	1.49	0.69
Outsider (Ref: No)						
Yes	1.38	0.28	0.12	0.92	2.07	0.12
Childhood Health (Ref: Good)						
Excellent	0.88	0.15	0.44	0.63	1.22	0.38
Very Good	0.80	0.10	0.07	0.62	1.02	.
Fair	0.74	0.17	0.20	0.47	1.17	.
Poor	0.84	0.39	0.71	0.34	2.09	.
Immigration Status (Ref: Born in This Country)						
Born in Another Country	2.69	1.18	0.02	1.14	6.36	0.02
Childhood Service Attendance (Ref: Never)						
At Least 1/Week	2.14	0.73	0.03	1.09	4.23	0.00
1-3/Month	1.25	0.44	0.52	0.63	2.50	.
<1/Month	1.29	0.48	0.49	0.62	2.67	.
Gender (Ref: Male)						
Female	1.69	0.17	0.00	1.38	2.07	0.00
Other	0.00	0.00	0.00	0.00	0.00	.
Year of Birth (Ref: 1998-2005)						
1993-1998; Age 25-29	0.85	0.26	0.61	0.47	1.56	0.00
1983-1993; Age 30-39	1.22	0.27	0.37	0.79	1.90	.
1973-1983; Age 40-49	1.20	0.26	0.41	0.78	1.84	.
1963-1973; Age 50-59	1.80	0.40	0.01	1.17	2.77	.
1953-1963; Age 60-69	2.77	0.67	0.00	1.73	4.44	.
1943-1953; Age 70-79	4.28	1.14	0.00	2.54	7.22	.
1943 or Earlier; Age 80 or Older	4.51	1.77	0.00	2.09	9.73	.
Mother Absence/Presence (Ref: Present)						
Absent	0.88	0.53	0.83	0.26	2.92	0.83
Father Absence/Presence (Ref: Present)						
Absent	0.77	0.30	0.51	0.36	1.66	0.51
Childhood Religion (Ref: No Religion/Atheist/Agnostic)						
Christianity	1.13	0.32	0.66	0.65	1.99	0.83
Some Other Religion	1.64	1.57	0.61	0.25	10.69	.

Race/Ethnicity (Ref: Ethnic Plurality)						
Ethnic Minority	0.53	0.36	0.35	0.14	2.01	0.35

Table S15c: E-Values and E-Value Limits (Poland)

Variable	E-Value	E-Value Limit
Relationship with Mother (Ref: Very/Somewhat Bad)		
Very/Somewhat Good	1.64	1.00
Relationship with Father (Ref: Very/Somewhat Bad)		
Very/Somewhat Good	2.31	1.00
Parent Marital Status (Ref: Married)		
Divorced	5.05	1.43
Never Married	14.35	2.12
One or Both Had Died	2.51	1.00
Childhood Income (Ref: Got By)		
Lived Comfortably	1.78	1.00
Found it Difficult	1.97	1.00
Found it Very Difficult	2.57	1.00
Childhood Abuse (Ref: No)		
Yes	1.68	1.00
Outsider (Ref: No)		
Yes	2.92	1.00
Childhood Health (Ref: Good)		
Excellent	1.84	1.00
Very Good	2.35	1.00
Fair	2.79	1.00
Poor	2.06	1.00
Immigration Status (Ref: Born in This Country)		
Born in Another Country	10.81	1.33
Childhood Service Attendance (Ref: Never)		
At Least 1/Week	7.08	1.25
1-3/Month	2.33	1.00
<1/Month	2.49	1.00
Gender (Ref: Male)		
Female	4.47	1.63
Other	5.41e+07	136.67
Year of Birth (Ref: 1998-2005)		
1993-1998; Age 25-29	1.98	1.00
1983-1993; Age 30-39	2.20	1.00
1973-1983; Age 40-49	2.09	1.00
1963-1973; Age 50-59	5.06	1.38
1953-1963; Age 60-69	11.40	1.96
1943-1953; Age 70-79	25.20	2.57
1943 or Earlier; Age 80 or Older	27.66	2.25
Mother Absence/Presence (Ref: Present)		
Absent	1.83	1.00
Father Absence/Presence (Ref: Present)		

Absent	2.51	1.00
Childhood Religion (Ref: No Religion/Atheist/Agnostic)		
Christianity	1.80	1.00
Some Other Religion	4.20	1.00
Race/Ethnicity (Ref: Ethnic Plurality)		
Ethnic Minority	5.49	1.00

Table S16a: Nationally Representative Descriptive Statistics of the Observed Sample (South Africa)

Variable	Proportion	Frequency
Relationship with Mother		
Very Good	0.82	2186
Somewhat Good	0.10	263
Somewhat Bad	0.02	51
Very Bad	0.01	39
Not Applicable	0.03	90
Missing	0.01	21
Relationship with Father		
Very Good	0.62	1656
Somewhat Good	0.13	333
Somewhat Bad	0.03	86
Very Bad	0.06	159
Not Applicable	0.12	331
Missing	0.03	85
Parent Marital Status		
Married	0.50	1321
Divorced	0.05	131
Never Married	0.34	904
One or Both Had Died	0.05	140
Missing	0.06	155
Childhood Income		
Lived Comfortably	0.40	1050
Got By	0.33	875
Found it Difficult	0.16	432
Found it Very Difficult	0.11	289
Missing	0.00	5
Childhood Abuse		
Yes	0.17	450
No	0.81	2149
Missing	0.02	52
Outsider		
Yes	0.16	434
No	0.83	2211
Not Applicable	0.00	3
Missing	0.00	3
Childhood Health		
Excellent	0.46	1225
Very Good	0.22	590

Good	0.14	370
Fair	0.10	266
Poor	0.07	183
Missing	0.01	17
Immigration Status		
Born in This Country	0.95	2511
Born in Another Country	0.05	139
Missing	0.00	1
Childhood Service Attendance		
At Least 1/Week	0.63	1681
1-3/Month	0.21	552
<1/Month	0.07	175
Never	0.08	217
Missing	0.01	26
Gender		
Male	0.49	1288
Female	0.51	1356
Other	0.00	2
Missing	0.00	4
Year of Birth		
1998-2005; Age 18-24	0.17	461
1993-1998; Age 25-29	0.14	364
1983-1993; Age 30-39	0.25	655
1973-1983; Age 40-49	0.20	522
1963-1973; Age 50-59	0.12	309
1953-1963; Age 60-69	0.07	195
1943-1953; Age 70-79	0.05	120
1943 or Earlier; 80 or Older	0.01	17
Missing	0.00	9
Childhood Religion		
Christianity	0.88	2323
Islam	0.02	52
Hinduism	0.00	2
Buddhism	0.00	11
Judaism	.	.
Sikhism	.	.
Baha'i	.	.
Jainism	.	.
Shinto	0.00	2
Taoism	0.00	1
Confucianism	.	.
Primal, Animist, or Folk Religion	0.04	117
Spiritism	.	.
African-Derived	.	.
Chinese	.	.
Some Other Religion	0.00	7
No Religion/Atheist/Agnostic	0.04	107
Missing	0.01	27
Race/Ethnicity		

Black	0.90	2381
Asian/Indian	0.00	6
Colored	0.10	252
White	0.00	8
Other	0.00	1
Missing	0.00	3

Table S16b: Variations Across Childhood Predictors (South Africa)

Variable	IRR	SE	Prob	LCI	UCI	Global p-value
Relationship with Mother (Ref: Very/Somewhat Bad)						
Very/Somewhat Good	0.85	0.13	0.29	0.62	1.16	0.29
Relationship with Father (Ref: Very/Somewhat Bad)						
Very/Somewhat Good	1.06	0.11	0.61	0.86	1.30	0.61
Parent Marital Status (Ref: Married)						
Divorced	0.96	0.14	0.77	0.71	1.28	0.55
Never Married	0.89	0.07	0.16	0.75	1.05	.
One or Both Had Died	0.98	0.18	0.91	0.69	1.39	.
Childhood Income (Ref: Got By)						
Lived Comfortably	1.01	0.08	0.85	0.87	1.18	0.09
Found it Difficult	0.80	0.09	0.04	0.64	0.99	.
Found it Very Difficult	1.05	0.13	0.70	0.83	1.33	.
Childhood Abuse (Ref: No)						
Yes	1.08	0.09	0.35	0.92	1.28	0.35
Outsider (Ref: No)						
Yes	1.05	0.10	0.60	0.87	1.26	0.60
Childhood Health (Ref: Good)						
Excellent	1.14	0.13	0.25	0.91	1.42	0.50
Very Good	1.08	0.14	0.57	0.84	1.38	.
Fair	0.92	0.15	0.60	0.67	1.26	.
Poor	1.19	0.20	0.31	0.85	1.65	.
Immigration Status (Ref: Born in This Country)						
Born in Another Country	0.65	0.16	0.09	0.39	1.07	0.09
Childhood Service Attendance (Ref: Never)						
At Least 1/Week	1.11	0.22	0.59	0.75	1.64	0.05
1-3/Month	0.97	0.21	0.90	0.64	1.48	.
<1/Month	0.70	0.18	0.16	0.42	1.16	.
Gender (Ref: Male)						
Female	1.21	0.09	0.01	1.04	1.41	0.00
Other	2.34	0.53	0.00	1.50	3.65	.
Year of Birth (Ref: 1998-2005)						
1993-1998; Age 25-29	0.90	0.10	0.37	0.72	1.13	0.01
1983-1993; Age 30-39	0.80	0.09	0.04	0.65	0.99	.
1973-1983; Age 40-49	1.04	0.11	0.68	0.85	1.28	.

1963-1973; Age 50-59	1.29	0.16	0.04	1.01	1.64	.
1953-1963; Age 60-69	1.16	0.18	0.36	0.85	1.57	.
1943-1953; Age 70-79	1.24	0.24	0.26	0.85	1.80	.
1943 or Earlier; Age 80 or Older	1.04	0.55	0.94	0.36	2.97	.
Mother Absence/Presence (Ref: Present)						
Absent	0.95	0.19	0.79	0.63	1.42	0.79
Father Absence/Presence (Ref: Present)						
Absent	1.08	0.12	0.52	0.86	1.34	0.52
Childhood Religion (Ref: No Religion/Atheist/Agnostic)						
Christianity	1.48	0.44	0.18	0.83	2.65	0.44
Primal, Animist, or Folk Religion	1.22	0.39	0.55	0.64	2.30	.
Some Other Religion	1.23	0.51	0.62	0.54	2.79	.
Race/Ethnicity (Ref: Ethnic Plurality)						
Ethnic Minority	1.14	0.17	0.38	0.85	1.52	0.38

Table S16c: E-Values and E-Value Limits (South Africa)

Variable	E-Value	E-Value Limit
Relationship with Mother (Ref: Very/Somewhat Bad)		
Very/Somewhat Good	1.57	1.00
Relationship with Father (Ref: Very/Somewhat Bad)		
Very/Somewhat Good	1.27	1.00
Parent Marital Status (Ref: Married)		
Divorced	1.23	1.00
Never Married	1.44	1.00
One or Both Had Died	1.15	1.00
Childhood Income (Ref: Got By)		
Lived Comfortably	1.12	1.00
Found it Difficult	1.72	1.09
Found it Very Difficult	1.24	1.00
Childhood Abuse (Ref: No)		
Yes	1.34	1.00
Outsider (Ref: No)		
Yes	1.25	1.00
Childhood Health (Ref: Good)		
Excellent	1.48	1.00
Very Good	1.32	1.00
Fair	1.36	1.00
Poor	1.58	1.00
Immigration Status (Ref: Born in This Country)		
Born in Another Country	2.25	1.00
Childhood Service Attendance (Ref: Never)		
At Least 1/Week	1.42	1.00
1-3/Month	1.18	1.00
<1/Month	2.06	1.00
Gender (Ref: Male)		

Female	1.63	1.16
Other	3.53	1.75
Year of Birth (Ref: 1998-2005)		
1993-1998; Age 25-29	1.41	1.00
1983-1993; Age 30-39	1.71	1.06
1973-1983; Age 40-49	1.23	1.00
1963-1973; Age 50-59	1.79	1.09
1953-1963; Age 60-69	1.51	1.00
1943-1953; Age 70-79	1.69	1.00
1943 or Earlier; Age 80 or Older	1.22	1.00
Mother Absence/Presence (Ref: Present)		
Absent	1.27	1.00
Father Absence/Presence (Ref: Present)		
Absent	1.32	1.00
Childhood Religion (Ref: No Religion/Atheist/Agnostic)		
Christianity	2.14	1.00
Primal, Animist, or Folk Religion	1.64	1.00
Some Other Religion	1.67	1.00
Race/Ethnicity (Ref: Ethnic Plurality)		
Ethnic Minority	1.47	1.00

Table S17a: Nationally Representative Descriptive Statistics of the Observed Sample (Spain)

Variable	Proportion	Frequency
Relationship with Mother		
Very Good	0.72	4557
Somewhat Good	0.20	1258
Somewhat Bad	0.04	248
Very Bad	0.01	92
Not Applicable	0.02	107
Missing	0.00	28
Relationship with Father		
Very Good	0.66	4131
Somewhat Good	0.22	1397
Somewhat Bad	0.05	309
Very Bad	0.03	178
Not Applicable	0.04	243
Missing	0.01	33
Parent Marital Status		
Married	0.84	5285
Divorced	0.06	378
Never Married	0.05	312
One or Both Had Died	0.02	126
Missing	0.03	188
Childhood Income		
Lived Comfortably	0.32	2041
Got By	0.47	2956
Found it Difficult	0.18	1154

Found it Very Difficult	0.02	110
Missing	0.00	29
Childhood Abuse		
Yes	0.10	659
No	0.88	5510
Missing	0.02	122
Outsider		
Yes	0.09	579
No	0.90	5637
Not Applicable	0.01	36
Missing	0.01	39
Childhood Health		
Excellent	0.39	2450
Very Good	0.36	2286
Good	0.20	1235
Fair	0.03	164
Poor	0.02	135
Missing	0.00	20
Immigration Status		
Born in This Country	0.87	5479
Born in Another Country	0.13	788
Missing	0.00	23
Childhood Service Attendance		
At Least 1/Week	0.38	2391
1-3/Month	0.18	1132
<1/Month	0.20	1287
Never	0.23	1445
Missing	0.01	36
Gender		
Male	0.50	3142
Female	0.50	3119
Other	0.00	6
Missing	0.00	22
Year of Birth		
1998-2005; Age 18-24	0.09	594
1993-1998; Age 25-29	0.07	450
1983-1993; Age 30-39	0.18	1111
1973-1983; Age 40-49	0.22	1396
1963-1973; Age 50-59	0.20	1252
1953-1963; Age 60-69	0.16	977
1943-1953; Age 70-79	0.07	467
1943 or Earlier; 80 or Older	0.01	43
Missing	.	.
Childhood Religion		
Christianity	0.81	5119
Islam	0.02	132
Hinduism	0.00	5
Buddhism	0.00	8
Judaism	0.00	5

Sikhism	0.00	2
Baha'i	.	.
Jainism	.	.
Shinto	.	.
Taoism	.	.
Confucianism	0.00	1
Primal, Animist, or Folk Religion	0.00	4
Spiritism	.	.
African-Derived	.	.
Chinese	.	.
Some Other Religion	0.00	13
No Religion/Atheist/Agnostic	0.15	972
Missing	0.00	29
Race/Ethnicity		
No Data	.	.

Table S17b: Variations Across Childhood Predictors (Spain)

Variable	IRR	SE	Prob	LCI	UCI	Global p-value
Relationship with Mother (Ref: Very/Somewhat Bad)						
Very/Somewhat Good	1.11	0.18	0.54	0.80	1.53	0.54
Relationship with Father (Ref: Very/Somewhat Bad)						
Very/Somewhat Good	1.23	0.16	0.10	0.96	1.59	0.10
Parent Marital Status (Ref: Married)						
Divorced	1.44	0.21	0.01	1.08	1.92	0.05
Never Married	1.28	0.18	0.08	0.97	1.69	.
One or Both Had Died	0.98	0.27	0.95	0.58	1.67	.
Childhood Income (Ref: Got By)						
Lived Comfortably	1.12	0.12	0.32	0.90	1.38	0.40
Found it Difficult	0.98	0.12	0.86	0.76	1.25	.
Found it Very Difficult	0.72	0.21	0.26	0.41	1.28	.
Childhood Abuse (Ref: No)						
Yes	1.14	0.14	0.30	0.89	1.44	0.30
Outsider (Ref: No)						
Yes	1.62	0.16	0.00	1.34	1.97	0.00
Childhood Health (Ref: Good)						
Excellent	1.29	0.16	0.04	1.01	1.65	0.02
Very Good	1.45	0.19	0.00	1.13	1.87	.
Fair	1.00	0.34	1.00	0.52	1.94	.
Poor	1.85	0.53	0.03	1.05	3.25	.
Immigration Status (Ref: Born in This Country)						
Born in Another Country	1.57	0.16	0.00	1.29	1.91	0.00
Childhood Service Attendance (Ref: Never)						
At Least 1/Week	3.13	0.55	0.00	2.22	4.42	0.00

1-3/Month	3.30	0.59	0.00	2.33	4.67	.
<1/Month	1.59	0.32	0.02	1.07	2.35	.
Gender (Ref: Male)						
Female	0.89	0.08	0.18	0.75	1.05	0.37
Other	1.20	0.72	0.76	0.37	3.92	.
Year of Birth (Ref: 1998-2005)						
1993-1998; Age 25-29	0.96	0.14	0.79	0.72	1.29	0.00
1983-1993; Age 30-39	0.88	0.12	0.34	0.68	1.14	.
1973-1983; Age 40-49	0.66	0.09	0.00	0.51	0.86	.
1963-1973; Age 50-59	0.64	0.10	0.00	0.47	0.87	.
1953-1963; Age 60-69	0.50	0.10	0.00	0.34	0.74	.
1943-1953; Age 70-79	1.11	0.25	0.64	0.71	1.73	.
1943 or Earlier; Age 80 or Older	1.95	0.79	0.10	0.88	4.32	.
Mother Absence/Presence (Ref: Present)						
Absent	2.06	0.39	0.00	1.41	3.00	0.00
Father Absence/Presence (Ref: Present)						
Absent	0.92	0.15	0.60	0.67	1.26	0.60
Childhood Religion (Ref: No Religion/Atheist/Agnostic)						
Christianity	1.17	0.20	0.35	0.84	1.65	0.00
Some Other Religion	3.40	0.67	0.00	2.31	5.01	.

Table S17c: E-Values and E-Value Limits (Spain)

Variable	E-Value	E-Value Limit
Relationship with Mother (Ref: Very/Somewhat Bad)		
Very/Somewhat Good	1.60	1.00
Relationship with Father (Ref: Very/Somewhat Bad)		
Very/Somewhat Good	2.07	1.00
Parent Marital Status (Ref: Married)		
Divorced	2.81	1.24
Never Married	2.23	1.00
One or Both Had Died	1.18	1.00
Childhood Income (Ref: Got By)		
Lived Comfortably	1.63	1.00
Found it Difficult	1.22	1.00
Found it Very Difficult	2.62	1.00
Childhood Abuse (Ref: No)		
Yes	1.70	1.00
Outsider (Ref: No)		
Yes	3.48	1.58
Childhood Health (Ref: Good)		
Excellent	2.27	1.06
Very Good	2.85	1.32
Fair	1.04	1.00
Poor	4.37	1.19
Immigration Status (Ref: Born in This Country)		

Born in Another Country	3.29	1.53
Childhood Service Attendance (Ref: Never)		
At Least 1/Week	10.18	2.34
1-3/Month	11.02	2.42
<1/Month	3.35	1.23
Gender (Ref: Male)		
Female	1.66	1.00
Other	1.95	1.00
Year of Birth (Ref: 1998-2005)		
1993-1998; Age 25-29	1.31	1.00
1983-1993; Age 30-39	1.70	1.00
1973-1983; Age 40-49	3.05	1.37
1963-1973; Age 50-59	3.28	1.35
1953-1963; Age 60-69	5.01	1.60
1943-1953; Age 70-79	1.61	1.00
1943 or Earlier; Age 80 or Older	4.78	1.00
Mother Absence/Presence (Ref: Present)		
Absent	5.21	1.66
Father Absence/Presence (Ref: Present)		
Absent	1.52	1.00
Childhood Religion (Ref: No Religion/Atheist/Agnostic)		
Christianity	1.85	1.00
Some Other Religion	11.57	2.41

Table S18a: Nationally Representative Descriptive Statistics of the Observed Sample (Sweden)

Variable	Proportion	Frequency
Relationship with Mother		
Very Good	0.58	8743
Somewhat Good	0.30	4513
Somewhat Bad	0.08	1194
Very Bad	0.02	372
Not Applicable	0.01	216
Missing	0.00	30
Relationship with Father		
Very Good	0.47	7134
Somewhat Good	0.32	4885
Somewhat Bad	0.11	1588
Very Bad	0.05	725
Not Applicable	0.05	720
Missing	0.00	16
Parent Marital Status		
Married	0.72	10887
Divorced	0.13	1927
Never Married	0.12	1747
One or Both Had Died	0.02	362
Missing	0.01	145
Childhood Income		

Lived Comfortably	0.39	5951
Got By	0.51	7717
Found it Difficult	0.08	1238
Found it Very Difficult	0.01	140
Missing	0.00	22
Childhood Abuse		
Yes	0.15	2288
No	0.85	12735
Missing	0.00	45
Outsider		
Yes	0.12	1867
No	0.86	13034
Not Applicable	0.01	139
Missing	0.00	29
Childhood Health		
Excellent	0.38	5733
Very Good	0.34	5124
Good	0.18	2669
Fair	0.07	1108
Poor	0.03	397
Missing	0.00	38
Immigration Status		
Born in This Country	0.92	13922
Born in Another Country	0.07	1052
Missing	0.01	94
Childhood Service Attendance		
At Least 1/Week	0.06	955
1-3/Month	0.09	1362
<1/Month	0.41	6224
Never	0.43	6472
Missing	0.00	54
Gender		
Male	0.50	7536
Female	0.50	7493
Other	0.00	27
Missing	0.00	12
Year of Birth		
1998-2005; Age 18-24	0.10	1515
1993-1998; Age 25-29	0.09	1399
1983-1993; Age 30-39	0.16	2398
1973-1983; Age 40-49	0.15	2221
1963-1973; Age 50-59	0.17	2493
1953-1963; Age 60-69	0.14	2168
1943-1953; Age 70-79	0.15	2253
1943 or Earlier; 80 or Older	0.04	621
Missing	.	.
Childhood Religion		
Christianity	0.70	10617
Islam	0.03	462

Hinduism	0.00	16
Buddhism	0.00	41
Judaism	0.00	51
Sikhism	0.00	9
Baha'i	0.00	3
Jainism	.	.
Shinto	0.00	1
Taoism	.	.
Confucianism	0.00	4
Primal, Animist, or Folk Religion	0.00	31
Spiritism	.	.
African-Derived	.	.
Chinese	.	.
Some Other Religion	0.00	69
No Religion/Atheist/Agnostic	0.25	3738
Missing	0.00	26
Race/Ethnicity		
No Data	.	.

Table S18b: Variations Across Childhood Predictors (Sweden)

Variable	IRR	SE	Prob	LCI	UCI	Global p-value
Relationship with Mother (Ref: Very/Somewhat Bad)						
Very/Somewhat Good	0.88	0.16	0.50	0.62	1.27	0.50
Relationship with Father (Ref: Very/Somewhat Bad)						
Very/Somewhat Good	1.06	0.17	0.73	0.77	1.45	0.73
Parent Marital Status (Ref: Married)						
Divorced	0.65	0.12	0.02	0.46	0.92	0.09
Never Married	1.09	0.18	0.60	0.79	1.52	.
One or Both Had Died	0.81	0.42	0.68	0.29	2.24	.
Childhood Income (Ref: Got By)						
Lived Comfortably	0.93	0.10	0.52	0.76	1.15	0.71
Found it Difficult	0.83	0.15	0.31	0.58	1.19	.
Found it Very Difficult	1.07	0.37	0.84	0.54	2.12	.
Childhood Abuse (Ref: No)						
Yes	1.32	0.16	0.03	1.03	1.68	0.03
Outsider (Ref: No)						
Yes	1.17	0.16	0.24	0.90	1.54	0.24
Childhood Health (Ref: Good)						
Excellent	0.91	0.13	0.48	0.69	1.19	0.37
Very Good	1.03	0.14	0.85	0.79	1.33	.
Fair	0.73	0.16	0.16	0.47	1.13	.
Poor	0.73	0.21	0.28	0.41	1.29	.
Immigration Status (Ref: Born in This Country)						

Born in Another Country	1.20	0.17	0.19	0.92	1.57	0.19
Childhood Service Attendance (Ref: Never)						
At Least 1/Week	18.39	3.45	0.00	12.73	26.57	0.00
1-3/Month	10.22	1.93	0.00	7.06	14.80	.
<1/Month	1.93	0.37	0.00	1.33	2.82	.
Gender (Ref: Male)						
Female	0.81	0.08	0.03	0.67	0.98	0.00
Other	0.00	0.00	0.00	0.00	0.00	.
Year of Birth (Ref: 1998-2005)						
1993-1998; Age 25-29	0.95	0.16	0.76	0.68	1.33	0.14
1983-1993; Age 30-39	0.79	0.13	0.16	0.57	1.10	.
1973-1983; Age 40-49	0.82	0.14	0.25	0.58	1.15	.
1963-1973; Age 50-59	0.84	0.15	0.34	0.59	1.20	.
1953-1963; Age 60-69	0.83	0.15	0.31	0.58	1.19	.
1943-1953; Age 70-79	0.59	0.12	0.01	0.40	0.87	.
1943 or Earlier; Age 80 or Older	0.56	0.15	0.03	0.32	0.95	.
Mother Absence/Presence (Ref: Present)						
Absent	1.71	0.49	0.06	0.97	3.00	0.06
Father Absence/Presence (Ref: Present)						
Absent	0.49	0.16	0.03	0.26	0.91	0.03
Childhood Religion (Ref: No Religion/Atheist/Agnostic)						
Christianity	1.45	0.29	0.07	0.98	2.16	0.00
Some Other Religion	3.02	0.74	0.00	1.87	4.88	.

Table S18c: E-Values and E-Value Limits (Sweden)

Variable	E-Value	E-Value Limit
Relationship with Mother (Ref: Very/Somewhat Bad)		
Very/Somewhat Good	2.00	1.00
Relationship with Father (Ref: Very/Somewhat Bad)		
Very/Somewhat Good	1.53	1.00
Parent Marital Status (Ref: Married)		
Divorced	4.90	1.25
Never Married	1.76	1.00
One or Both Had Died	2.65	1.00
Childhood Income (Ref: Got By)		
Lived Comfortably	1.62	1.00
Found it Difficult	2.46	1.00
Found it Very Difficult	1.65	1.00
Childhood Abuse (Ref: No)		
Yes	3.20	1.15
Outsider (Ref: No)		
Yes	2.26	1.00
Childhood Health (Ref: Good)		
Excellent	1.83	1.00

Very Good	1.32	1.00
Fair	3.53	1.00
Poor	3.61	1.00
Immigration Status (Ref: Born in This Country)		
Born in Another Country	2.43	1.00
Childhood Service Attendance (Ref: Never)		
At Least 1/Week	1729.05	6.59
1-3/Month	441.70	4.76
<1/Month	8.72	1.57
Gender (Ref: Male)		
Female	2.64	1.10
Other	7.27e+12	672.95
Year of Birth (Ref: 1998-2005)		
1993-1998; Age 25-29	1.51	1.00
1983-1993; Age 30-39	2.82	1.00
1973-1983; Age 40-49	2.57	1.00
1963-1973; Age 50-59	2.37	1.00
1953-1963; Age 60-69	2.47	1.00
1943-1953; Age 70-79	6.23	1.36
1943 or Earlier; Age 80 or Older	7.25	1.18
Mother Absence/Presence (Ref: Present)		
Absent	6.39	1.00
Father Absence/Presence (Ref: Present)		
Absent	10.16	1.26
Childhood Religion (Ref: No Religion/Atheist/Agnostic)		
Christianity	4.19	1.00
Some Other Religion	25.57	2.08

Table S19a: Nationally Representative Descriptive Statistics of the Observed Sample (Tanzania)

Variable	Proportion	Frequency
Relationship with Mother		
Very Good	0.85	7739
Somewhat Good	0.09	796
Somewhat Bad	0.01	84
Very Bad	0.01	84
Not Applicable	0.03	303
Missing	0.01	70
Relationship with Father		
Very Good	0.75	6831
Somewhat Good	0.12	1101
Somewhat Bad	0.02	203
Very Bad	0.03	247
Not Applicable	0.06	550
Missing	0.02	142
Parent Marital Status		
Married	0.76	6929

Divorced	0.07	678
Never Married	0.08	751
One or Both Had Died	0.03	313
Missing	0.04	404
Childhood Income		
Lived Comfortably	0.29	2611
Got By	0.32	2909
Found it Difficult	0.30	2679
Found it Very Difficult	0.09	814
Missing	0.01	61
Childhood Abuse		
Yes	0.08	716
No	0.92	8328
Missing	0.00	32
Outsider		
Yes	0.08	734
No	0.92	8320
Not Applicable	0.00	4
Missing	0.00	17
Childhood Health		
Excellent	0.27	2406
Very Good	0.22	2036
Good	0.32	2946
Fair	0.13	1177
Poor	0.05	456
Missing	0.01	54
Immigration Status		
Born in This Country	1.00	9048
Born in Another Country	0.00	25
Missing	0.00	1
Childhood Service Attendance		
At Least 1/Week	0.61	5580
1-3/Month	0.26	2383
<1/Month	0.04	333
Never	0.07	595
Missing	0.02	184
Gender		
Male	0.47	4299
Female	0.53	4776
Other	.	.
Missing	.	.
Year of Birth		
1998-2005; Age 18-24	0.25	2284
1993-1998; Age 25-29	0.15	1349
1983-1993; Age 30-39	0.23	2060
1973-1983; Age 40-49	0.17	1503
1963-1973; Age 50-59	0.10	912
1953-1963; Age 60-69	0.06	575
1943-1953; Age 70-79	0.03	297

1943 or Earlier; 80 or Older	0.01	93
Missing	0.00	2
Childhood Religion		
Christianity	0.62	5651
Islam	0.34	3060
Hinduism	.	.
Buddhism	.	.
Judaism	.	.
Sikhism	.	.
Baha'i	0.00	1
Jainism	.	.
Shinto	.	.
Taoism	.	.
Confucianism	.	.
Primal, Animist, or Folk Religion	0.00	11
Spiritism	.	.
African-Derived	.	.
Chinese	.	.
Some Other Religion	.	.
No Religion/Atheist/Agnostic	0.04	345
Missing	0.00	7
Race/Ethnicity		
African	1.00	9060
Indian	0.00	3
Arab	0.00	11
Other	.	.
Missing	0.00	2

Table S19b: Variations Across Childhood Predictors (Tanzania)

Variable	IRR	SE	Prob	LCI	UCI	Global p-value
Relationship with Mother (Ref: Very/Somewhat Bad)						
Very/Somewhat Good	0.98	0.05	0.66	0.89	1.08	0.66
Relationship with Father (Ref: Very/Somewhat Bad)						
Very/Somewhat Good	1.03	0.04	0.55	0.94	1.11	0.55
Parent Marital Status (Ref: Married)						
Divorced	0.96	0.04	0.29	0.89	1.04	0.04
Never Married	0.90	0.04	0.01	0.83	0.98	.
One or Both Had Died	0.94	0.05	0.25	0.85	1.05	.
Childhood Income (Ref: Got By)						
Lived Comfortably	0.98	0.02	0.25	0.94	1.02	0.20
Found it Difficult	0.96	0.02	0.07	0.91	1.00	.
Found it Very Difficult	0.95	0.04	0.13	0.88	1.02	.
Childhood Abuse (Ref: No)						
Yes	0.93	0.04	0.07	0.86	1.01	0.07

Outsider (Ref: No)						
Yes	0.98	0.03	0.53	0.91	1.05	0.53
Childhood Health (Ref: Good)						
Excellent	1.05	0.02	0.02	1.01	1.10	0.10
Very Good	1.06	0.03	0.02	1.01	1.11	.
Fair	1.04	0.03	0.12	0.99	1.10	.
Poor	1.04	0.05	0.37	0.95	1.14	.
Immigration Status (Ref: Born in This Country)						
Born in Another Country	1.23	0.08	0.00	1.08	1.40	0.00
Childhood Service Attendance (Ref: Never)						
At Least 1/Week	1.17	0.07	0.01	1.04	1.33	0.02
1-3/Month	1.19	0.07	0.01	1.05	1.34	.
<1/Month	1.06	0.08	0.42	0.92	1.23	.
Gender (Ref: Male)						
Female	1.02	0.02	0.24	0.99	1.06	0.24
Other	1.00	.	.	.	.	.
Year of Birth (Ref: 1998-2005)						
1993-1998; Age 25-29	1.04	0.03	0.21	0.98	1.10	0.08
1983-1993; Age 30-39	1.07	0.03	0.01	1.02	1.12	.
1973-1983; Age 40-49	1.08	0.03	0.01	1.02	1.14	.
1963-1973; Age 50-59	1.09	0.03	0.01	1.03	1.16	.
1953-1963; Age 60-69	1.12	0.05	0.01	1.03	1.22	.
1943-1953; Age 70-79	1.09	0.07	0.21	0.95	1.24	.
1943 or Earlier; Age 80 or Older	1.07	0.11	0.50	0.87	1.32	.
Mother Absence/Presence (Ref: Present)						
Absent	0.97	0.05	0.62	0.88	1.08	0.62
Father Absence/Presence (Ref: Present)						
Absent	1.05	0.04	0.25	0.97	1.13	0.25
Childhood Religion (Ref: No Religion/Atheist/Agnostic)						
Christianity	1.00	0.08	1.00	0.86	1.16	0.15
Islam	1.05	0.09	0.53	0.90	1.23	.
Some Other Religion	0.73	0.23	0.32	0.40	1.35	.
Race/Ethnicity (Ref: Ethnic Plurality)						
Ethnic Minority	0.93	0.18	0.72	0.64	1.37	0.72

Table S19c: E-Values and E-Value Limits (Tanzania)

Variable	E-Value	E-Value Limit
Relationship with Mother (Ref: Very/Somewhat Bad)		
Very/Somewhat Good	1.13	1.00
Relationship with Father (Ref: Very/Somewhat Bad)		
Very/Somewhat Good	1.14	1.00
Parent Marital Status (Ref: Married)		
Divorced	1.19	1.00
Never Married	1.33	1.12

One or Both Had Died	1.23	1.00
Childhood Income (Ref: Got By)		
Lived Comfortably	1.14	1.00
Found it Difficult	1.19	1.00
Found it Very Difficult	1.22	1.00
Childhood Abuse (Ref: No)		
Yes	1.26	1.00
Outsider (Ref: No)		
Yes	1.13	1.00
Childhood Health (Ref: Good)		
Excellent	1.21	1.07
Very Good	1.22	1.08
Fair	1.19	1.00
Poor	1.19	1.00
Immigration Status (Ref: Born in This Country)		
Born in Another Country	1.52	1.24
Childhood Service Attendance (Ref: Never)		
At Least 1/Week	1.44	1.16
1-3/Month	1.46	1.19
<1/Month	1.23	1.00
Gender (Ref: Male)		
Female	1.13	1.00
Other	1.00	1.00
Year of Birth (Ref: 1998-2005)		
1993-1998; Age 25-29	1.17	1.00
1983-1993; Age 30-39	1.25	1.11
1973-1983; Age 40-49	1.27	1.10
1963-1973; Age 50-59	1.29	1.13
1953-1963; Age 60-69	1.35	1.14
1943-1953; Age 70-79	1.28	1.00
1943 or Earlier; Age 80 or Older	1.26	1.00
Mother Absence/Presence (Ref: Present)		
Absent	1.14	1.00
Father Absence/Presence (Ref: Present)		
Absent	1.20	1.00
Childhood Religion (Ref: No Religion/Atheist/Agnostic)		
Christianity	1.01	1.00
Islam	1.21	1.00
Some Other Religion	1.70	1.00
Race/Ethnicity (Ref: Ethnic Plurality)		
Ethnic Minority	1.26	1.00

Table S20a: Nationally Representative Descriptive Statistics of the Observed Sample (Turkey)

Variable	Proportion	Frequency
Relationship with Mother		
Very Good	0.66	970
Somewhat Good	0.27	401

Somewhat Bad	0.03	48
Very Bad	0.02	26
Not Applicable	0.01	21
Missing	0.00	7
Relationship with Father		
Very Good	0.54	795
Somewhat Good	0.29	425
Somewhat Bad	0.05	73
Very Bad	0.06	95
Not Applicable	0.04	60
Missing	0.02	25
Parent Marital Status		
Married	0.90	1325
Divorced	0.04	57
Never Married	0.00	7
One or Both Had Died	0.04	61
Missing	0.02	23
Childhood Income		
Lived Comfortably	0.34	498
Got By	0.44	647
Found it Difficult	0.15	218
Found it Very Difficult	0.07	108
Missing	0.00	2
Childhood Abuse		
Yes	0.11	158
No	0.88	1290
Missing	0.02	25
Outsider		
Yes	0.11	157
No	0.89	1306
Not Applicable	0.00	5
Missing	0.00	5
Childhood Health		
Excellent	0.26	377
Very Good	0.28	410
Good	0.28	419
Fair	0.15	220
Poor	0.03	47
Missing	0.00	0
Immigration Status		
Born in This Country	0.96	1415
Born in Another Country	0.04	58
Missing	.	.
Childhood Service Attendance		
At Least 1/Week	0.41	609
1-3/Month	0.16	238
<1/Month	0.15	225
Never	0.26	383
Missing	0.01	18

Gender		
Male	0.51	754
Female	0.49	719
Other	.	.
Missing	.	.
Year of Birth		
1998-2005; Age 18-24	0.15	222
1993-1998; Age 25-29	0.10	152
1983-1993; Age 30-39	0.21	315
1973-1983; Age 40-49	0.21	312
1963-1973; Age 50-59	0.15	225
1953-1963; Age 60-69	0.11	164
1943-1953; Age 70-79	0.04	65
1943 or Earlier; 80 or Older	0.01	18
Missing	.	.
Childhood Religion		
Christianity	0.00	1
Islam	0.98	1439
Hinduism	.	.
Buddhism	.	.
Judaism	0.00	1
Sikhism	.	.
Baha'i	.	.
Jainism	.	.
Shinto	.	.
Taoism	.	.
Confucianism	.	.
Primal, Animist, or Folk Religion	.	.
Spiritism	.	.
African-Derived	.	.
Chinese	.	.
Some Other Religion	.	.
No Religion/Atheist/Agnostic	0.01	13
Missing	0.01	19
Race/Ethnicity		
Turkish	0.70	1030
Kurdish/Zaza	0.17	252
Arab	0.03	51
Laz	0.02	25
Circassian	0.01	19
Bosnian	0.00	5
Armenian	0.00	1
Georgian	0.00	4
Uyghur	0.00	1
Jewish	.	.
Albanian	0.01	8
Greek	0.00	1
Azeri	0.01	9
Other	0.04	58

Missing	0.01	9

Table S20b: Variations Across Childhood Predictors (Turkey)

Variable	IRR	SE	Prob	LCI	UCI	Global p-value
Relationship with Mother (Ref: Very/Somewhat Bad)						
Very/Somewhat Good	0.79	0.12	0.13	0.58	1.07	0.13
Relationship with Father (Ref: Very/Somewhat Bad)						
Very/Somewhat Good	1.19	0.16	0.21	0.91	1.55	0.21
Parent Marital Status (Ref: Married)						
Divorced	1.12	0.19	0.49	0.81	1.57	0.67
Never Married	0.65	0.33	0.40	0.24	1.78	.
One or Both Had Died	0.89	0.18	0.57	0.60	1.33	.
Childhood Income (Ref: Got By)						
Lived Comfortably	1.09	0.09	0.29	0.93	1.29	0.24
Found it Difficult	1.04	0.11	0.71	0.84	1.29	.
Found it Very Difficult	1.33	0.19	0.05	1.00	1.77	.
Childhood Abuse (Ref: No)						
Yes	0.80	0.11	0.11	0.61	1.05	0.11
Outsider (Ref: No)						
Yes	0.98	0.13	0.90	0.76	1.28	0.90
Childhood Health (Ref: Good)						
Excellent	0.89	0.08	0.20	0.75	1.06	0.03
Very Good	0.77	0.08	0.01	0.63	0.94	.
Fair	0.84	0.10	0.13	0.66	1.06	.
Poor	0.46	0.16	0.02	0.24	0.90	.
Immigration Status (Ref: Born in This Country)						
Born in Another Country	0.84	0.23	0.53	0.49	1.45	0.53
Childhood Service Attendance (Ref: Never)						
At Least 1/Week	2.67	0.34	0.00	2.09	3.42	0.00
1-3/Month	1.57	0.24	0.00	1.17	2.11	.
<1/Month	1.03	0.18	0.85	0.73	1.47	.
Gender (Ref: Male)						
Female	1.63	0.12	0.00	1.41	1.88	0.00
Other	1.00	.	.	.	.	.
Year of Birth (Ref: 1998-2005)						
1993-1998; Age 25-29	1.25	0.17	0.10	0.96	1.63	0.00
1983-1993; Age 30-39	1.30	0.16	0.04	1.02	1.67	.
1973-1983; Age 40-49	1.08	0.14	0.54	0.84	1.41	.
1963-1973; Age 50-59	1.23	0.18	0.16	0.92	1.63	.
1953-1963; Age 60-69	1.27	0.21	0.15	0.92	1.74	.
1943-1953; Age 70-79	1.39	0.32	0.16	0.88	2.19	.
1943 or Earlier; Age 80 or Older	2.97	0.58	0.00	2.03	4.37	.

Mother Absence/Presence (Ref: Present)						
Absent	0.86	0.20	0.52	0.54	1.36	0.52
Father Absence/Presence (Ref: Present)						
Absent	1.15	0.26	0.54	0.74	1.79	0.54
Childhood Religion (Ref: Islam)						
Some Other Religion	0.66	0.29	0.34	0.28	1.56	0.34
Race/Ethnicity (Ref: Ethnic Plurality)						
Ethnic Minority	1.04	0.08	0.63	0.89	1.22	0.63

Table S20c: E-Values and E-Value Limits (Turkey)

Variable	E-Value	E-Value Limit
Relationship with Mother (Ref: Very/Somewhat Bad)		
Very/Somewhat Good	1.71	1.00
Relationship with Father (Ref: Very/Somewhat Bad)		
Very/Somewhat Good	1.56	1.00
Parent Marital Status (Ref: Married)		
Divorced	1.43	1.00
Never Married	2.18	1.00
One or Both Had Died	1.43	1.00
Childhood Income (Ref: Got By)		
Lived Comfortably	1.36	1.00
Found it Difficult	1.22	1.00
Found it Very Difficult	1.83	1.01
Childhood Abuse (Ref: No)		
Yes	1.69	1.00
Outsider (Ref: No)		
Yes	1.13	1.00
Childhood Health (Ref: Good)		
Excellent	1.42	1.00
Very Good	1.77	1.22
Fair	1.58	1.00
Poor	3.14	1.29
Immigration Status (Ref: Born in This Country)		
Born in Another Country	1.57	1.00
Childhood Service Attendance (Ref: Never)		
At Least 1/Week	3.84	2.25
1-3/Month	2.23	1.37
<1/Month	1.20	1.00
Gender (Ref: Male)		
Female	2.32	1.66
Other	1.00	1.00
Year of Birth (Ref: 1998-2005)		
1993-1998; Age 25-29	1.68	1.00
1983-1993; Age 30-39	1.78	1.10
1973-1983; Age 40-49	1.34	1.00
1963-1973; Age 50-59	1.64	1.00

1953-1963; Age 60-69	1.71	1.00
1943-1953; Age 70-79	1.93	1.00
1943 or Earlier; Age 80 or Older	4.25	2.20
Mother Absence/Presence (Ref: Present)		
Absent	1.51	1.00
Father Absence/Presence (Ref: Present)		
Absent	1.48	1.00
Childhood Religion (Ref: Islam)		
Some Other Religion	2.14	1.00
Race/Ethnicity (Ref: Ethnic Plurality)		
Ethnic Minority	1.21	1.00

Table S21a: Nationally Representative Descriptive Statistics of the Observed Sample (United Kingdom)

Variable	Proportion	Frequency
Relationship with Mother		
Very Good	0.64	3435
Somewhat Good	0.25	1338
Somewhat Bad	0.06	325
Very Bad	0.03	150
Not Applicable	0.02	92
Missing	0.01	27
Relationship with Father		
Very Good	0.54	2907
Somewhat Good	0.26	1383
Somewhat Bad	0.08	407
Very Bad	0.06	321
Not Applicable	0.06	321
Missing	0.01	29
Parent Marital Status		
Married	0.81	4343
Divorced	0.09	481
Never Married	0.06	315
One or Both Had Died	0.03	154
Missing	0.01	75
Childhood Income		
Lived Comfortably	0.48	2552
Got By	0.36	1933
Found it Difficult	0.12	632
Found it Very Difficult	0.04	230
Missing	0.00	22
Childhood Abuse		
Yes	0.16	864
No	0.83	4455
Missing	0.01	49
Outsider		
Yes	0.19	1017

No	0.80	4308
Not Applicable	0.01	32
Missing	0.00	12
Childhood Health		
Excellent	0.40	2154
Very Good	0.32	1736
Good	0.19	995
Fair	0.06	332
Poor	0.02	130
Missing	0.00	20
Immigration Status		
Born in This Country	0.87	4659
Born in Another Country	0.13	682
Missing	0.00	27
Childhood Service Attendance		
At Least 1/Week	0.32	1732
1-3/Month	0.14	733
<1/Month	0.17	903
Never	0.37	1972
Missing	0.01	28
Gender		
Male	0.48	2557
Female	0.52	2789
Other	0.00	14
Missing	0.00	9
Year of Birth		
1998-2005; Age 18-24	0.09	490
1993-1998; Age 25-29	0.07	391
1983-1993; Age 30-39	0.18	946
1973-1983; Age 40-49	0.15	827
1963-1973; Age 50-59	0.18	949
1953-1963; Age 60-69	0.17	889
1943-1953; Age 70-79	0.13	711
1943 or Earlier; 80 or Older	0.03	163
Missing	0.00	1
Childhood Religion		
Christianity	0.64	3461
Islam	0.04	230
Hinduism	0.02	88
Buddhism	0.00	15
Judaism	0.01	59
Sikhism	0.01	30
Baha'i	0.00	5
Jainism	0.00	0
Shinto	.	.
Taoism	0.00	2
Confucianism	0.00	3
Primal, Animist, or Folk Religion	0.00	22
Spiritism	.	.

African-Derived	.	.
Chinese	.	.
Some Other Religion	0.00	24
No Religion/Atheist/Agnostic	0.26	1409
Missing	0.00	21
Race/Ethnicity		
Asian	0.08	426
Black	0.03	152
White	0.87	4647
Other	0.02	96
Missing	0.01	47

Table S21b: Variations Across Childhood Predictors (United Kingdom)

Variable	IRR	SE	Prob	LCI	UCI	Global p-value
Relationship with Mother (Ref: Very/Somewhat Bad)						
Very/Somewhat Good	1.09	0.19	0.64	0.77	1.54	0.64
Relationship with Father (Ref: Very/Somewhat Bad)						
Very/Somewhat Good	1.44	0.22	0.01	1.08	1.93	0.01
Parent Marital Status (Ref: Married)						
Divorced	0.75	0.17	0.19	0.48	1.16	0.61
Never Married	0.85	0.23	0.56	0.50	1.45	.
One or Both Had Died	0.92	0.33	0.82	0.46	1.85	.
Childhood Income (Ref: Got By)						
Lived Comfortably	0.95	0.10	0.64	0.78	1.17	0.41
Found it Difficult	1.09	0.17	0.58	0.80	1.48	.
Found it Very Difficult	0.69	0.18	0.16	0.42	1.16	.
Childhood Abuse (Ref: No)						
Yes	1.15	0.14	0.23	0.91	1.45	0.23
Outsider (Ref: No)						
Yes	1.63	0.17	0.00	1.34	2.00	0.00
Childhood Health (Ref: Good)						
Excellent	1.11	0.15	0.43	0.85	1.46	0.70
Very Good	1.01	0.14	0.96	0.77	1.32	.
Fair	1.14	0.25	0.55	0.74	1.77	.
Poor	0.76	0.28	0.46	0.37	1.58	.
Immigration Status (Ref: Born in This Country)						
Born in Another Country	1.11	0.12	0.35	0.89	1.38	0.35
Childhood Service Attendance (Ref: Never)						
At Least 1/Week	5.85	1.25	0.00	3.85	8.89	0.00
1-3/Month	4.59	1.06	0.00	2.92	7.21	.
<1/Month	1.83	0.44	0.01	1.14	2.93	.
Gender (Ref: Male)						
Female	0.74	0.07	0.00	0.62	0.89	0.00

Other	0.40	0.42	0.39	0.05	3.19	.
Year of Birth (Ref: 1998-2005)						
1993-1998; Age 25-29	1.96	0.48	0.01	1.21	3.17	0.00
1983-1993; Age 30-39	2.08	0.48	0.00	1.32	3.28	.
1973-1983; Age 40-49	1.49	0.37	0.10	0.92	2.41	.
1963-1973; Age 50-59	1.19	0.31	0.50	0.72	1.97	.
1953-1963; Age 60-69	1.12	0.31	0.69	0.65	1.92	.
1943-1953; Age 70-79	1.17	0.32	0.57	0.68	2.01	.
1943 or Earlier; Age 80 or Older	1.07	0.34	0.84	0.57	2.00	.
Mother Absence/Presence (Ref: Present)						
Absent	1.66	0.36	0.02	1.09	2.53	0.02
Father Absence/Presence (Ref: Present)						
Absent	0.98	0.23	0.92	0.61	1.56	0.92
Childhood Religion (Ref: No Religion/Atheist/Agnostic)						
Christianity	1.38	0.26	0.09	0.95	2.01	0.00
Islam	2.56	0.57	0.00	1.66	3.95	.
Some Other Religion	1.44	0.42	0.21	0.81	2.54	.
Race/Ethnicity (Ref: Ethnic Plurality)						
Ethnic Minority	1.50	0.22	0.01	1.13	1.99	0.01

Table S21c: E-Values and E-Value Limits (United Kingdom)

Variable	E-Value	E-Value Limit
Relationship with Mother (Ref: Very/Somewhat Bad)		
Very/Somewhat Good	1.49	1.00
Relationship with Father (Ref: Very/Somewhat Bad)		
Very/Somewhat Good	2.69	1.23
Parent Marital Status (Ref: Married)		
Divorced	2.34	1.00
Never Married	1.80	1.00
One or Both Had Died	1.47	1.00
Childhood Income (Ref: Got By)		
Lived Comfortably	1.33	1.00
Found it Difficult	1.50	1.00
Found it Very Difficult	2.68	1.00
Childhood Abuse (Ref: No)		
Yes	1.73	1.00
Outsider (Ref: No)		
Yes	3.33	1.58
Childhood Health (Ref: Good)		
Excellent	1.59	1.00
Very Good	1.11	1.00
Fair	1.69	1.00
Poor	2.27	1.00
Immigration Status (Ref: Born in This Country)		
Born in Another Country	1.58	1.00

Childhood Service Attendance (Ref: Never)		
At Least 1/Week	22.03	3.33
1-3/Month	15.64	2.81
<1/Month	4.00	1.33
Gender (Ref: Male)		
Female	2.37	1.31
Other	6.44	1.00
Year of Birth (Ref: 1998-2005)		
1993-1998; Age 25-29	4.47	1.43
1983-1993; Age 30-39	4.91	1.57
1973-1983; Age 40-49	2.86	1.00
1963-1973; Age 50-59	1.86	1.00
1953-1963; Age 60-69	1.60	1.00
1943-1953; Age 70-79	1.79	1.00
1943 or Earlier; Age 80 or Older	1.41	1.00
Mother Absence/Presence (Ref: Present)		
Absent	3.42	1.25
Father Absence/Presence (Ref: Present)		
Absent	1.22	1.00
Childhood Religion (Ref: No Religion/Atheist/Agnostic)		
Christianity	2.49	1.00
Islam	6.71	1.89
Some Other Religion	2.67	1.00
Race/Ethnicity (Ref: Ethnic Plurality)		
Ethnic Minority	2.87	1.32

Table S22a: Nationally Representative Descriptive Statistics of the Observed Sample (United States)

Variable	Proportion	Frequency
Relationship with Mother		
Very Good	0.54	20590
Somewhat Good	0.30	11525
Somewhat Bad	0.09	3523
Very Bad	0.05	1874
Not Applicable	0.02	694
Missing	0.00	106
Relationship with Father		
Very Good	0.40	15313
Somewhat Good	0.33	12666
Somewhat Bad	0.13	4879
Very Bad	0.07	2604
Not Applicable	0.07	2811
Missing	0.00	38
Parent Marital Status		
Married	0.72	27415
Divorced	0.17	6325
Never Married	0.08	3048

One or Both Had Died	0.03	1024
Missing	0.01	500
Childhood Income		
Lived Comfortably	0.39	15116
Got By	0.41	15682
Found it Difficult	0.13	5152
Found it Very Difficult	0.06	2342
Missing	0.00	19
Childhood Abuse		
Yes	0.26	10026
No	0.73	28045
Missing	0.01	242
Outsider		
Yes	0.27	10185
No	0.72	27714
Not Applicable	0.01	305
Missing	0.00	109
Childhood Health		
Excellent	0.44	16866
Very Good	0.32	12108
Good	0.17	6444
Fair	0.06	2303
Poor	0.01	520
Missing	0.00	71
Immigration Status		
Born in This Country	0.91	34865
Born in Another Country	0.08	3020
Missing	0.01	427
Childhood Service Attendance		
At Least 1/Week	0.49	18609
1-3/Month	0.17	6644
<1/Month	0.15	5829
Never	0.18	7085
Missing	0.00	145
Gender		
Male	0.48	18222
Female	0.51	19562
Other	0.01	392
Missing	0.00	136
Year of Birth		
1998-2005; Age 18-24	0.07	2682
1993-1998; Age 25-29	0.09	3540
1983-1993; Age 30-39	0.19	7284
1973-1983; Age 40-49	0.15	5649
1963-1973; Age 50-59	0.18	6745
1953-1963; Age 60-69	0.18	6832
1943-1953; Age 70-79	0.11	4054
1943 or Earlier; 80 or Older	0.04	1525
Missing	.	.

Childhood Religion		
Christianity	0.79	30444
Islam	0.01	220
Hinduism	0.01	203
Buddhism	0.00	172
Judaism	0.02	787
Sikhism	0.00	47
Baha'i	0.00	4
Jainism	0.00	18
Shinto	0.00	6
Taoism	0.00	17
Confucianism	0.00	8
Primal, Animist, or Folk Religion	0.00	67
Spiritism	.	.
African-Derived	.	.
Chinese	.	.
Some Other Religion	0.01	359
No Religion/Atheist/Agnostic	0.15	5845
Missing	0.00	115
Race/Ethnicity		
White	0.62	23605
Other	0.03	997
Black	0.12	4501
Asian	0.06	2466
Hispanic	0.18	6724
Other	.	.
Missing	0.00	20

Table S22b: Variations Across Childhood Predictors (United States)

Variable	IRR	SE	Prob	LCI	UCI	Global p-value
Relationship with Mother (Ref: Very/Somewhat Bad)						
Very/Somewhat Good	1.04	0.09	0.67	0.87	1.23	0.67
Relationship with Father (Ref: Very/Somewhat Bad)						
Very/Somewhat Good	1.11	0.08	0.13	0.97	1.28	0.13
Parent Marital Status (Ref: Married)						
Divorced	1.00	0.08	0.98	0.86	1.17	0.16
Never Married	1.34	0.18	0.03	1.02	1.75	.
One or Both Had Died	1.16	0.23	0.45	0.79	1.70	.
Childhood Income (Ref: Got By)						
Lived Comfortably	0.94	0.04	0.18	0.85	1.03	0.09
Found it Difficult	0.87	0.07	0.07	0.74	1.01	.
Found it Very Difficult	1.17	0.15	0.22	0.91	1.49	.
Childhood Abuse (Ref: No)						
Yes	1.02	0.05	0.71	0.92	1.13	0.71

Outsider (Ref: No)						
Yes	0.77	0.05	0.00	0.68	0.88	0.00
Childhood Health (Ref: Good)						
Excellent	1.04	0.08	0.58	0.90	1.22	0.21
Very Good	1.04	0.08	0.67	0.88	1.21	.
Fair	0.82	0.16	0.32	0.56	1.21	.
Poor	1.67	0.43	0.04	1.01	2.75	.
Immigration Status (Ref: Born in This Country)						
Born in Another Country	0.89	0.09	0.28	0.72	1.10	0.28
Childhood Service Attendance (Ref: Never)						
At Least 1/Week	2.18	0.22	0.00	1.80	2.65	0.00
1-3/Month	1.57	0.18	0.00	1.25	1.96	.
<1/Month	1.28	0.16	0.05	1.00	1.64	.
Gender (Ref: Male)						
Female	1.31	0.06	0.00	1.20	1.44	0.00
Other	0.36	0.17	0.03	0.14	0.92	.
Year of Birth (Ref: 1998-2005)						
1993-1998; Age 25-29	1.15	0.28	0.56	0.72	1.84	0.00
1983-1993; Age 30-39	0.98	0.20	0.94	0.66	1.47	.
1973-1983; Age 40-49	1.14	0.23	0.51	0.77	1.71	.
1963-1973; Age 50-59	1.35	0.27	0.13	0.91	2.01	.
1953-1963; Age 60-69	1.51	0.30	0.04	1.02	2.22	.
1943-1953; Age 70-79	1.40	0.28	0.09	0.95	2.07	.
1943 or Earlier; Age 80 or Older	1.64	0.35	0.02	1.08	2.48	.
Mother Absence/Presence (Ref: Present)						
Absent	0.89	0.15	0.48	0.63	1.24	0.48
Father Absence/Presence (Ref: Present)						
Absent	1.07	0.13	0.55	0.85	1.36	0.55
Childhood Religion (Ref: No Religion/Atheist/Agnostic)						
Christianity	1.56	0.20	0.00	1.22	2.00	0.00
Some Other Religion	1.34	0.29	0.17	0.88	2.03	.
Race/Ethnicity (Ref: Ethnic Plurality)						
Ethnic Minority	1.39	0.07	0.00	1.26	1.52	0.00

Table S22c: E-Values and E-Value Limits (United States)

Variable	E-Value	E-Value Limit
Relationship with Mother (Ref: Very/Somewhat Bad)		
Very/Somewhat Good	1.25	1.00
Relationship with Father (Ref: Very/Somewhat Bad)		
Very/Somewhat Good	1.50	1.00
Parent Marital Status (Ref: Married)		
Divorced	1.05	1.00
Never Married	2.10	1.12
One or Both Had Died	1.63	1.00

Childhood Income (Ref: Got By)		
Lived Comfortably	1.35	1.00
Found it Difficult	1.61	1.00
Found it Very Difficult	1.65	1.00
Childhood Abuse (Ref: No)		
Yes	1.17	1.00
Outsider (Ref: No)		
Yes	1.99	1.34
Childhood Health (Ref: Good)		
Excellent	1.27	1.00
Very Good	1.24	1.00
Fair	1.78	1.00
Poor	2.91	1.09
Immigration Status (Ref: Born in This Country)		
Born in Another Country	1.53	1.00
Childhood Service Attendance (Ref: Never)		
At Least 1/Week	4.14	2.02
1-3/Month	2.65	1.48
<1/Month	1.95	1.00
Gender (Ref: Male)		
Female	2.04	1.42
Other	5.51	1.26
Year of Birth (Ref: 1998-2005)		
1993-1998; Age 25-29	1.60	1.00
1983-1993; Age 30-39	1.15	1.00
1973-1983; Age 40-49	1.58	1.00
1963-1973; Age 50-59	2.14	1.00
1953-1963; Age 60-69	2.51	1.12
1943-1953; Age 70-79	2.26	1.00
1943 or Earlier; Age 80 or Older	2.83	1.25
Mother Absence/Presence (Ref: Present)		
Absent	1.55	1.00
Father Absence/Presence (Ref: Present)		
Absent	1.38	1.00
Childhood Religion (Ref: No Religion/Atheist/Agnostic)		
Christianity	2.64	1.44
Some Other Religion	2.10	1.00
Race/Ethnicity (Ref: Ethnic Plurality)		
Ethnic Minority	2.22	1.50

Table S23. Population weighted meta-analysis of regression results.

Variable	Category	RR	95% CI
Relationship with mother	(Ref: Very bad/somewhat bad)		
	Very/somewhat good	1.01	(0.91,1.14)
Relationship with father	(Ref: Very bad/somewhat bad)		

Variable	Category	RR	95% CI
	Very/somewhat good	1.08	(0.98,1.19)
Parent marital status	(Ref: Parents married)		
	No, divorced	0.97	(0.84,1.13)
	Single, never married	1.08	(0.91,1.26)
	No, one or both had died	0.91	(0.71,1.18)
Subjective financial status of family growing up	(Ref: Got by)		
	Lived comfortably	0.99	(0.93,1.06)
	Found it difficult	0.92	(0.83,1.02)
	Found it very difficult	0.85	(0.69,1.04)
Abuse	(Ref: No)		
	Yes	1.15	(1.07,1.24)
Outsider growing up	(Ref: No)		
	Yes	1.13	(1.05,1.21)
Self-rated health growing up	(Ref: Good)		
	Excellent	1.13	(1.03,1.24)
	Very good	1.07	(0.98,1.18)
	Fair	0.79	(0.67,0.94)
	Poor	0.95	(0.74,1.22)
Immigration status	(Ref: Born in this country)		
	No	0.87	(0.75,1.00)
Age 12 religious service attendance	(Ref: Never)		
	At least 1/week	2.96	(2.52,3.48)
	1-3/month	2.45	(2.08,2.90)
	Less than 1/month	1.46	(1.22,1.75)
Year of birth	(Ref: 1998-2005; age 18-24)		
	1993-1998; age 25-29	1.15	(0.98,1.34)
	1983-1993; age 30-39	1.12	(0.97,1.29)

Variable	Category	RR	95% CI
	1973-1983; age 40-49	1.17	(1.01,1.34)
	1963-1973; age 50-59	1.25	(1.08,1.45)
	1953-1963; age 60-69	1.26	(1.07,1.47)
	1943-1953; age 70-79	1.24	(1.03,1.50)
	1943 or earlier; age 80+	0.06	(0.04,0.09)
Gender	(Ref: Male)		
	Female	1.05	(0.99,1.11)
	Other	0.03	(0.02,0.06)

Table S24. Population weighted meta-analysis of E-values.

Variable	Category	evalue	evalue.limit
Relationship with mother	(Ref: Very bad/somewhat bad)		
	Very/somewhat good	1.13	1.00
Relationship with father	(Ref: Very bad/somewhat bad)		
	Very/somewhat good	1.37	1.00
Parent marital status	(Ref: Parents married)		
	No, divorced	1.21	1.00
	Single, never married	1.36	1.00
	No, one or both had died	1.42	1.00
Subjective financial status of family growing up	(Ref: Got by)		
	Lived comfortably	1.10	1.00
	Found it difficult	1.41	1.00
	Found it very difficult	1.63	1.00
Abuse	(Ref: No)		
	Yes	1.57	1.36
Outsider growing up	(Ref: No)		
	Yes	1.51	1.29
Self-rated health growing up	(Ref: Good)		
	Excellent	1.51	1.21
	Very good	1.35	1.00
	Fair	1.84	1.34
	Poor	1.30	1.00

Variable	Category	evalue	evalue.limit
Immigration status	(Ref: Born in this country)		
	No	1.57	1.04
Age 12 religious service attendance	(Ref: Never)		
	At least 1/week	5.37	4.47
	1-3/month	4.34	3.57
	Less than 1/month	2.27	1.73
Year of birth	(Ref: 1998-2005; age 18-24)		
	1993-1998; age 25-29	1.56	1.00
	1983-1993; age 30-39	1.48	1.00
	1973-1983; age 40-49	1.60	1.11
	1963-1973; age 50-59	1.82	1.38
	1953-1963; age 60-69	1.83	1.36
	1943-1953; age 70-79	1.79	1.19
	1943 or earlier; age 80+	33.14	22.07
Gender	(Ref: Male)		
	Female	1.29	1.00
	Other	61.07	31.27

Figure S1. Forest plot for `Relationship with mother`-`Very/somewhat good` effect

Relationship with mother (Ref: Very bad/somewhat bad)

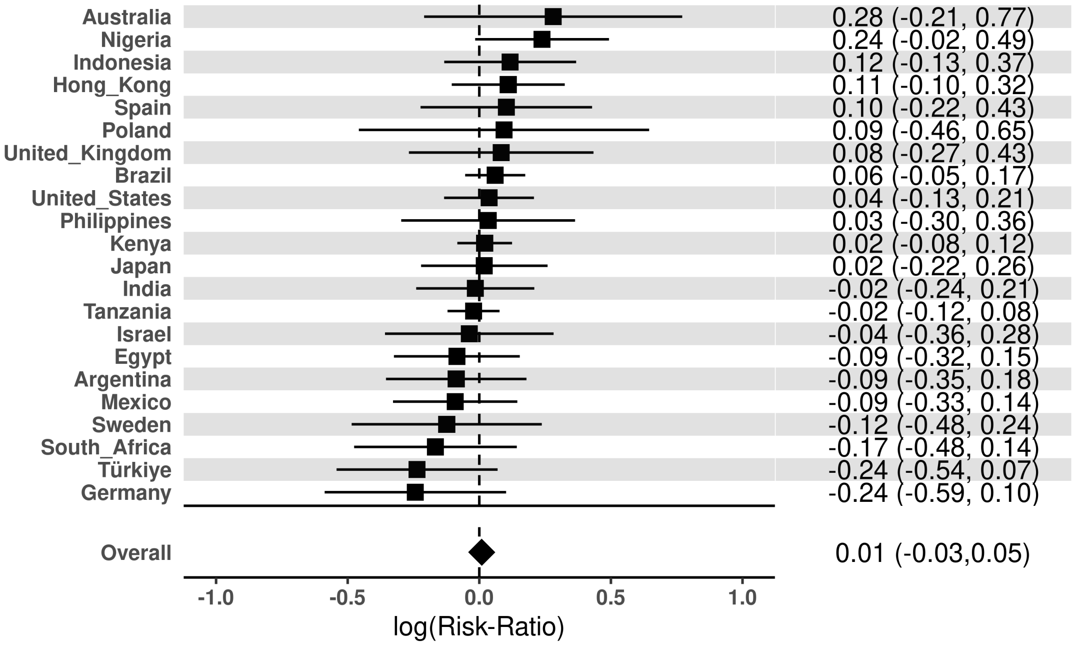

τ=0.000; Q-profile 95% CI [0.000, 0.072]; I^2=0.00;

Figure S2. Forest plot for `Relationship with father`-`Very/somewhat good` effect

Relationship with father (Ref: Very bad/somewhat bad)

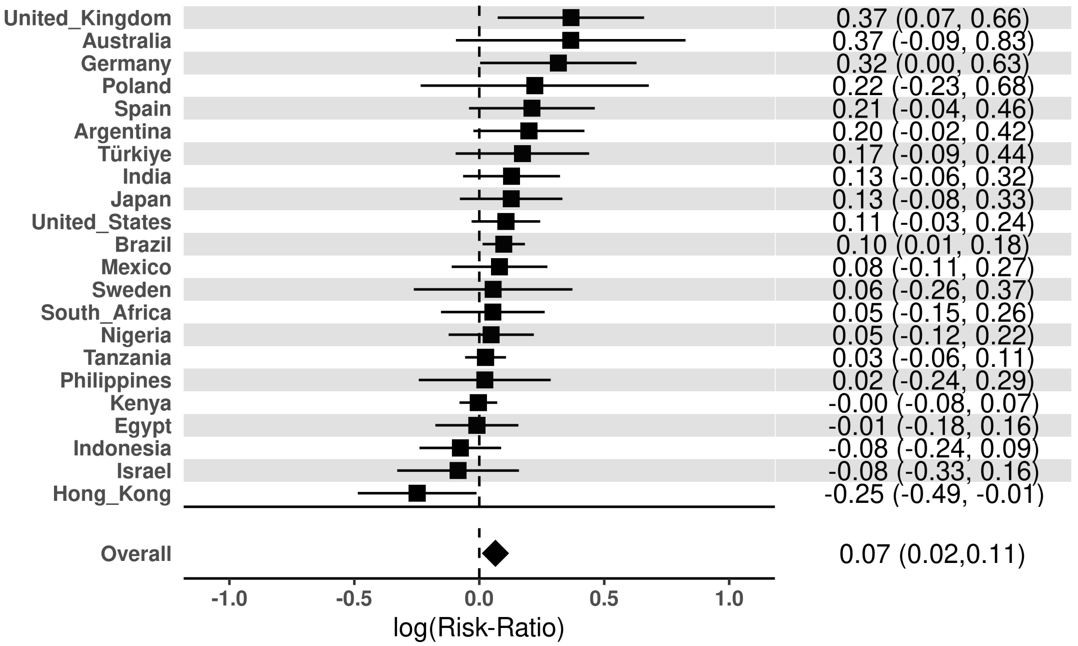

τ=0.064; Q-profile 95% CI [0.000, 0.115]; I^2=36.58;

Figure S3. Forest plot for `Parent marital status`-`No, divorced` effect

Parent marital status (Ref: Parents married)

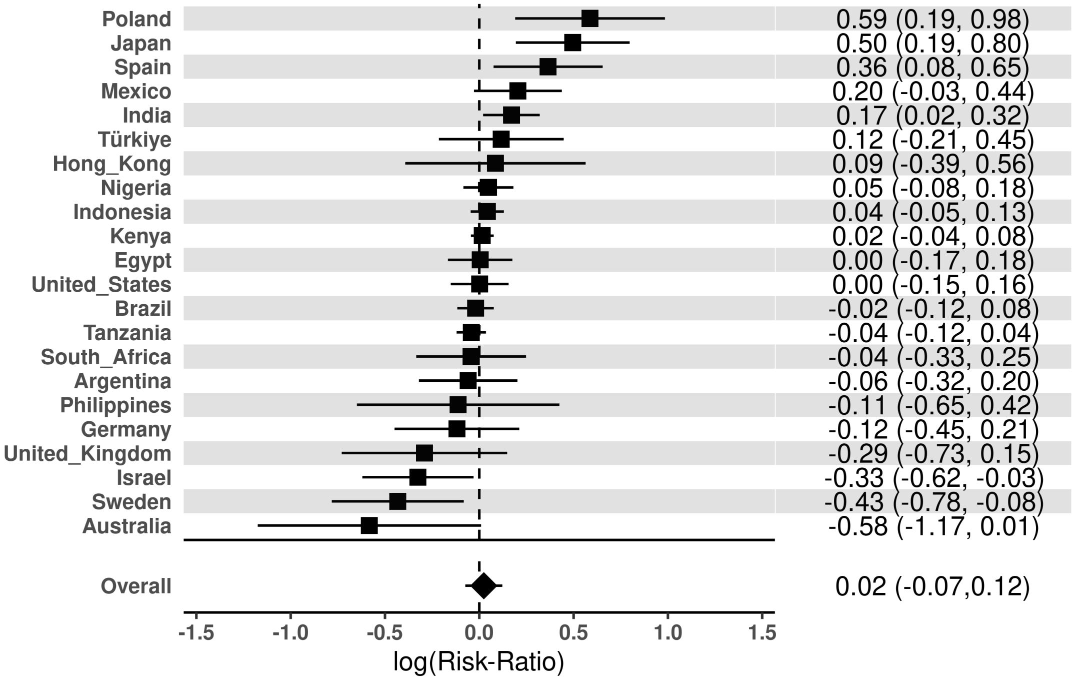

τ=0.192; Q-profile 95% CI [0.000, 0.271]; I^2=84.97;

Figure S4. Forest plot for `Parent marital status`-`Single, never married` effect

Parent marital status (Ref: Parents married)

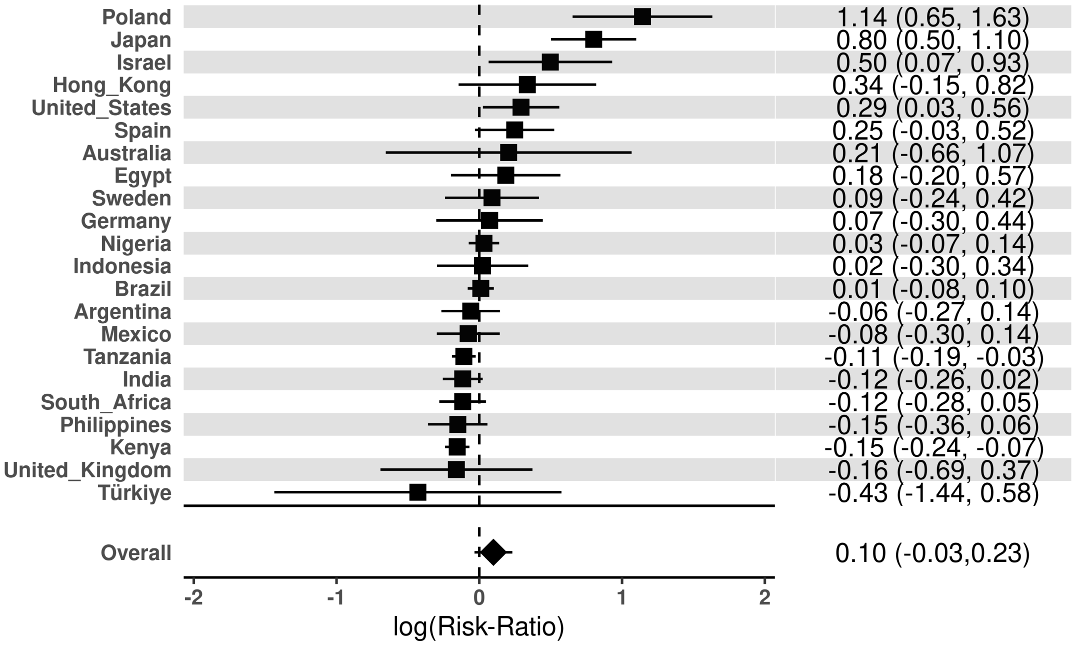

τ=0.272; Q-profile 95% CI [0.144, 0.389]; I^2=89.69;

Figure S5. Forest plot for `Parent marital status`-`No, one or both had died` effect

Parent marital status (Ref: Parents married)

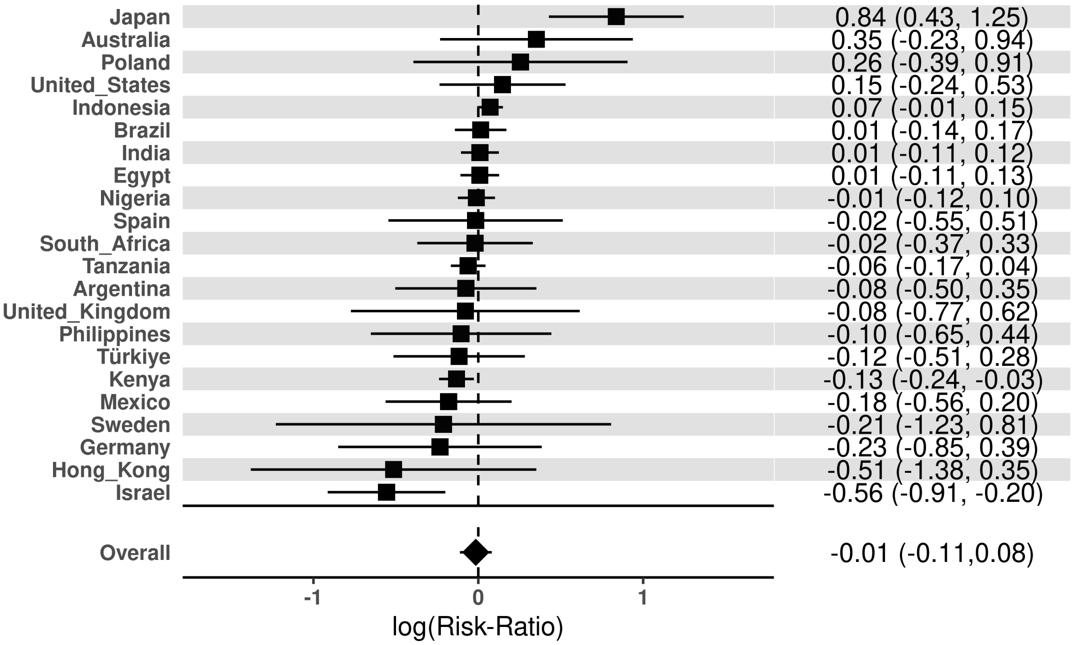

τ=0.159; Q-profile 95% CI [0.000, 0.253]; I^2=73.12;

Figure S6. Forest plot for `Subjective financial status of family growing up`-`Lived comfortably` effect

Subjective financial status of family growing up (Ref: Got by)

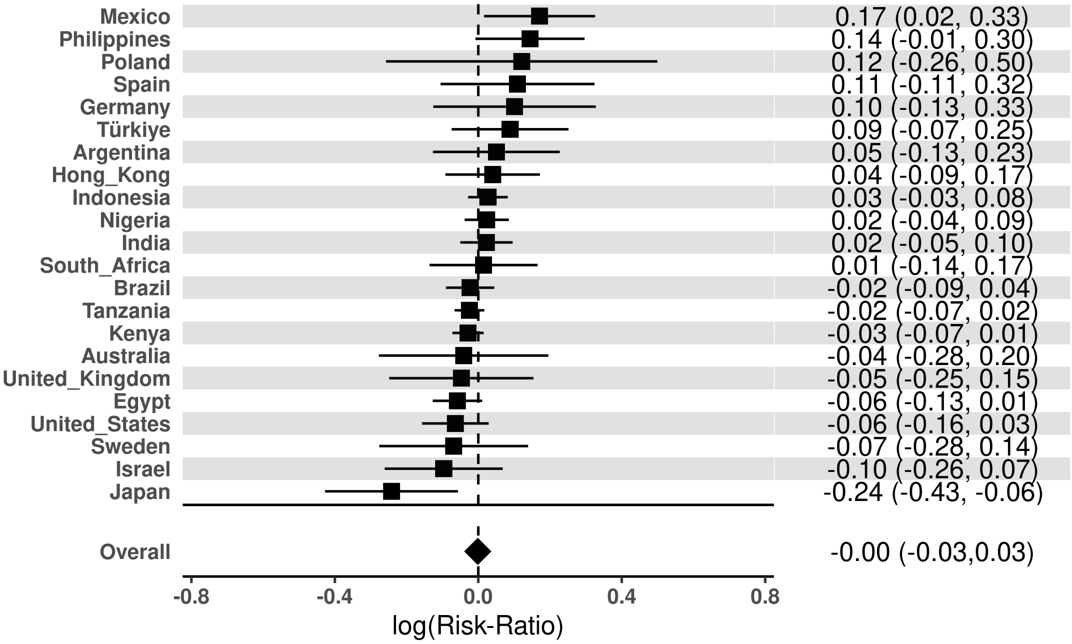

τ=0.040; Q-profile 95% CI [0.000, 0.069]; I^2=42.45;

Figure S7. Forest plot for `Subjective financial status of family growing up`-`Found it difficult` effect

Subjective financial status of family growing up (Ref: Got by)

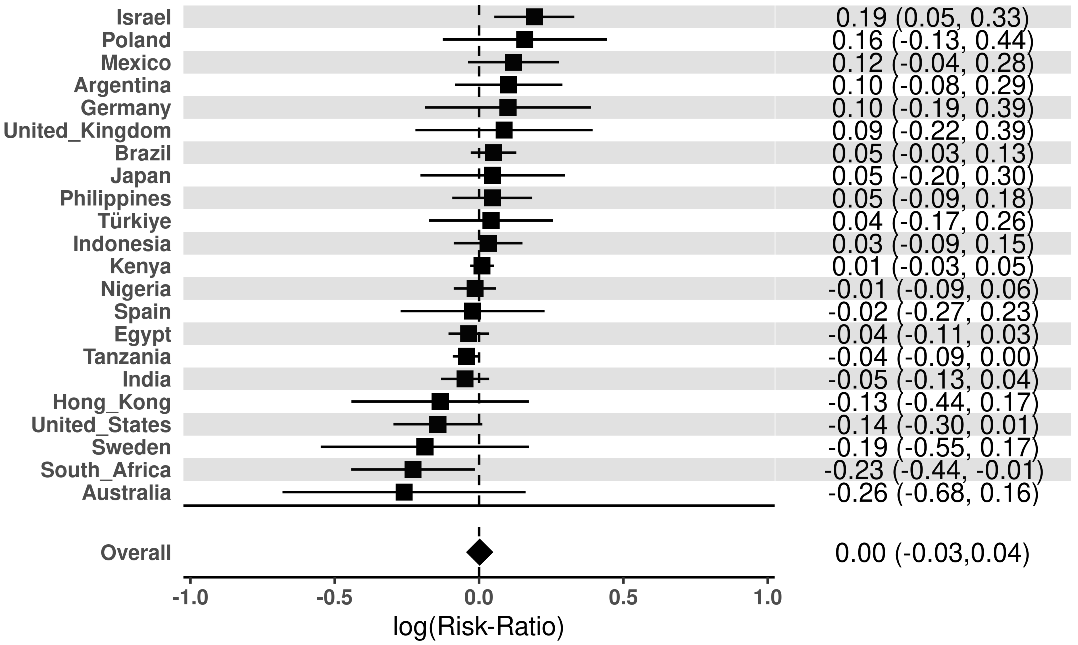

τ=0.050; Q-profile 95% CI [0.000, 0.091]; I^2=45.66;

Figure S8. Forest plot for `Subjective financial status of family growing up`-`Found it very difficult` effect

Subjective financial status of family growing up (Ref: Got by)

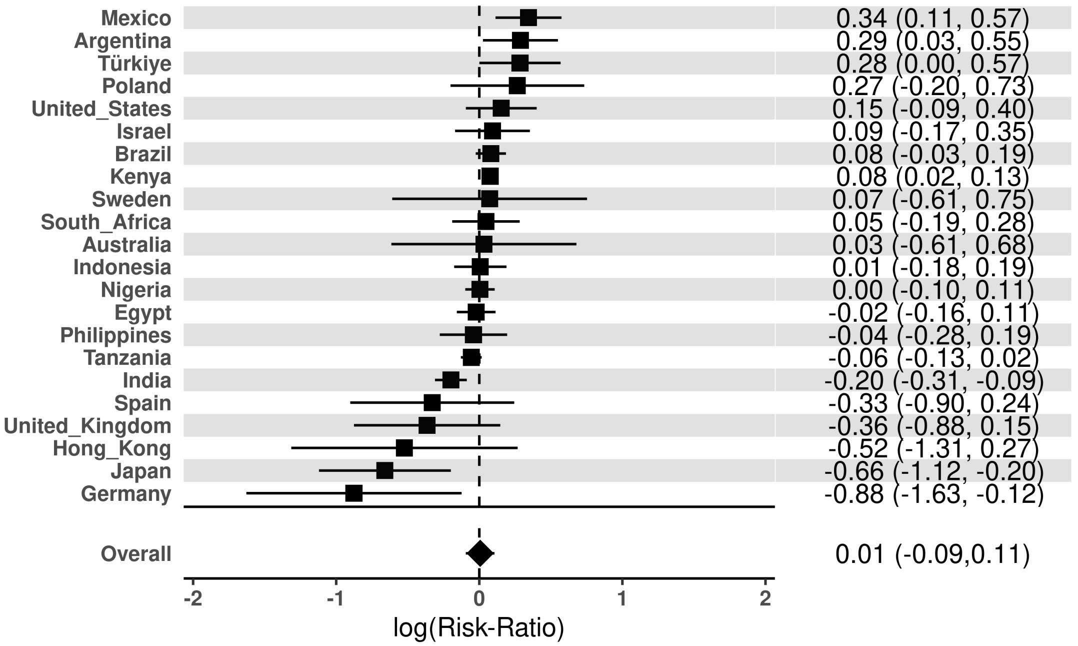

τ=0.187; Q-profile 95% CI [0.038, 0.262]; I^2=83.79;

Figure S9. Forest plot for `Abuse`-`Yes` effect

Abuse (Ref: No)

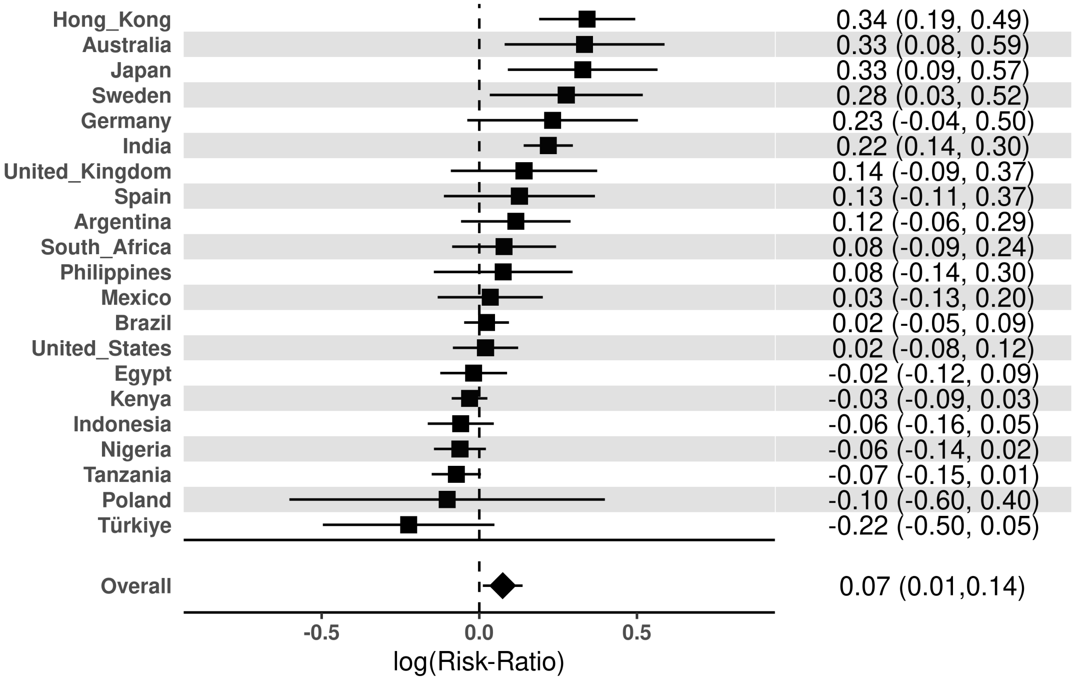

τ=0.119; Q-profile 95% CI [0.069, 0.181]; I^2=79.08;
Excluded countries: Israel

Figure S10. Forest plot for `Outsider growing up`-`Yes` effect

Outsider growing up (Ref: No)

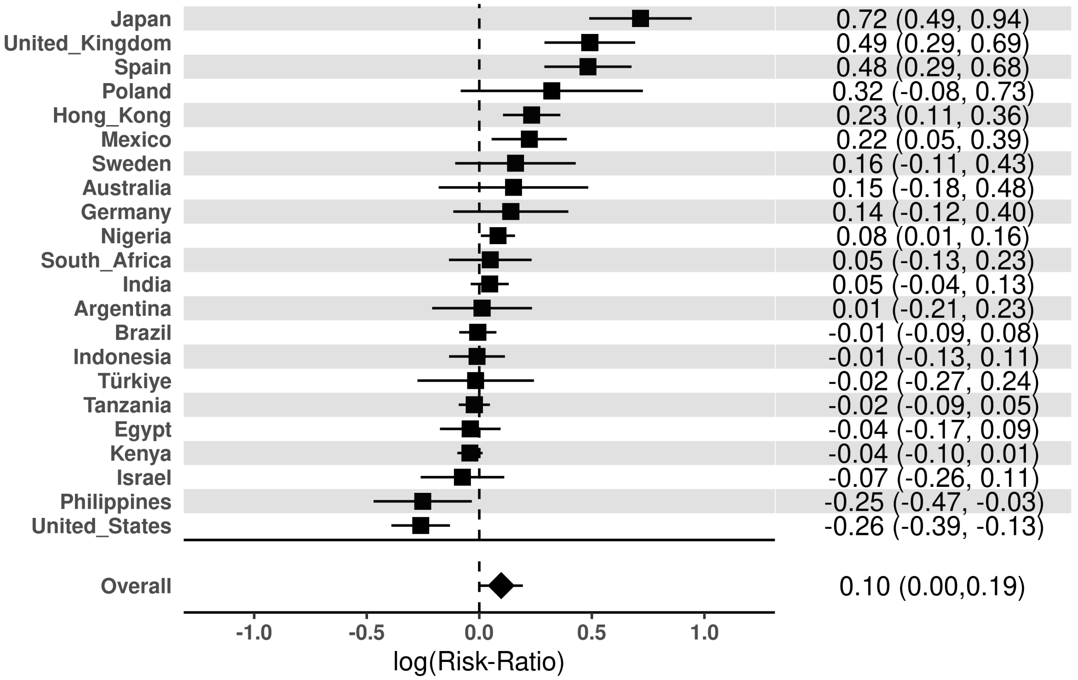

τ=0.210; Q-profile 95% CI [0.139, 0.297]; I^2=91.51;

Figure S11. Forest plot for `Self-rated health growing up`-`Excellent` effect

Self-rated health growing up (Ref: Good)

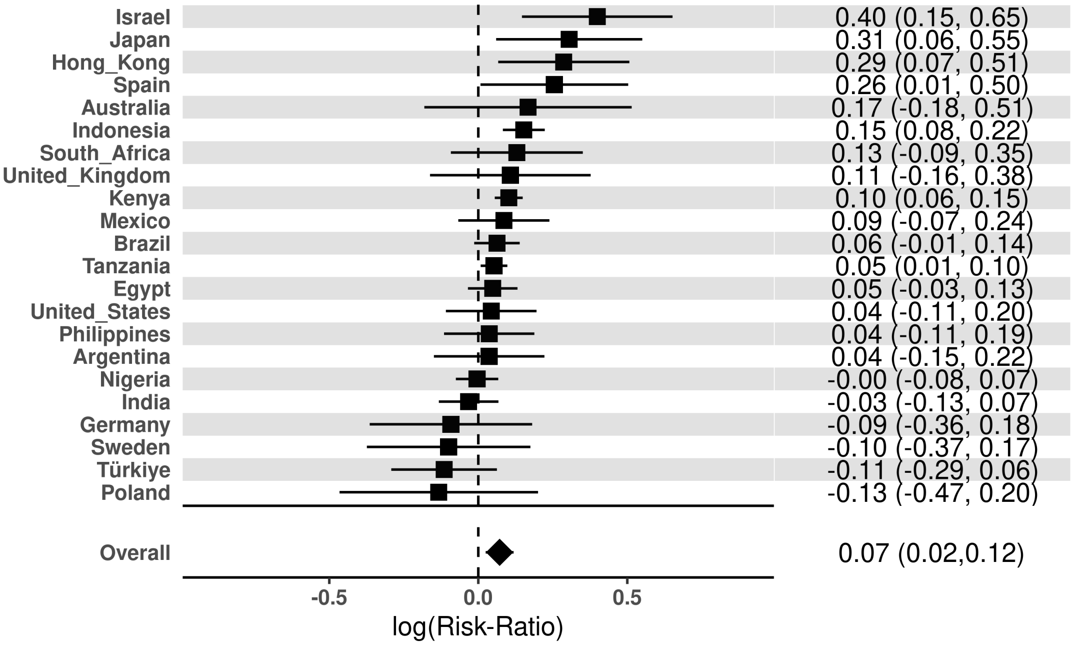

τ=0.078; Q-profile 95% CI [0.000, 0.118]; I^2=66.90;

Figure S12. Forest plot for `Self-rated health growing up`-`Very good` effect

Self-rated health growing up (Ref: Good)

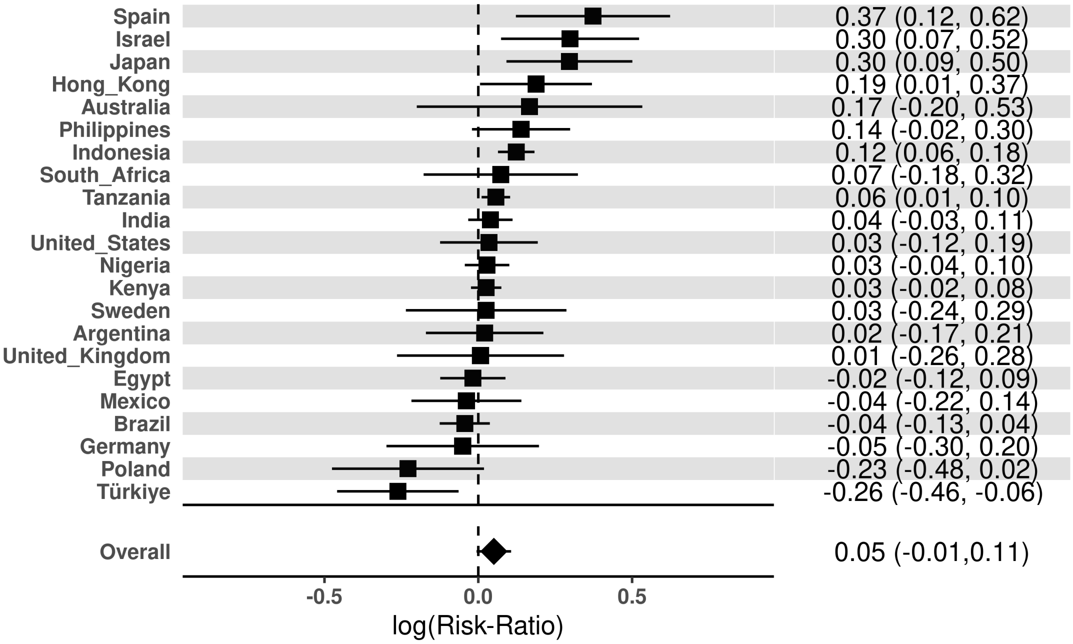

τ=0.104; Q-profile 95% CI [0.000, 0.153]; I^2=78.55;

Figure S13. Forest plot for `Self-rated health growing up`-`Fair` effect

Self-rated health growing up (Ref: Good)

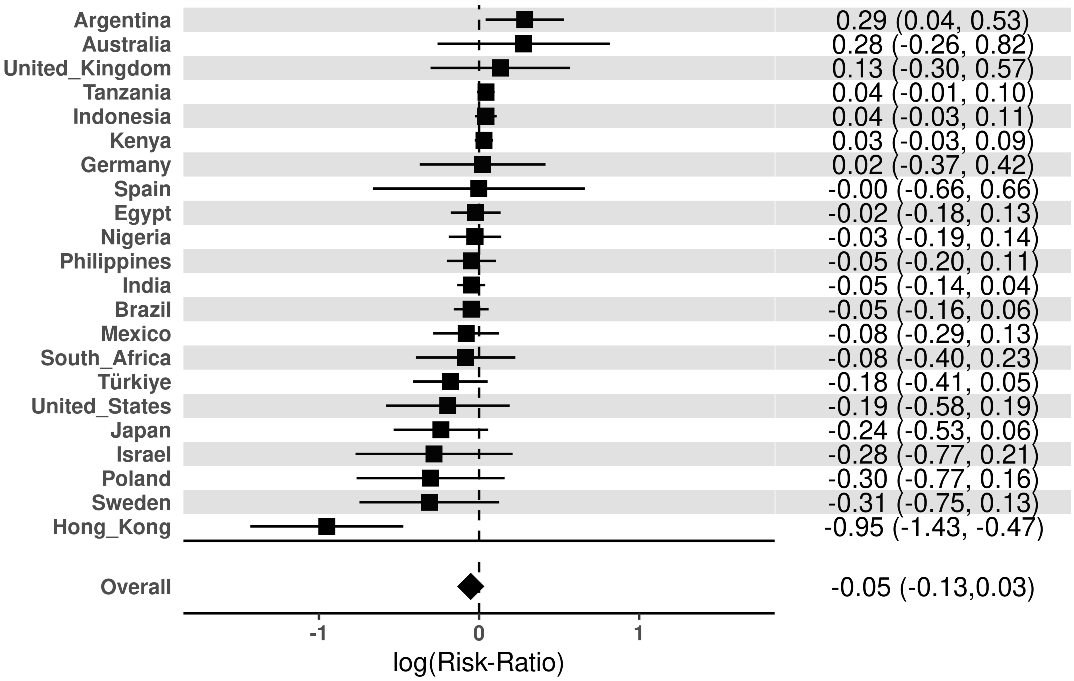

τ=0.136; Q-profile 95% CI [0.000, 0.194]; I^2=78.97;

Figure S14. Forest plot for `Self-rated health growing up`-`Poor` effect

Self-rated health growing up (Ref: Good)

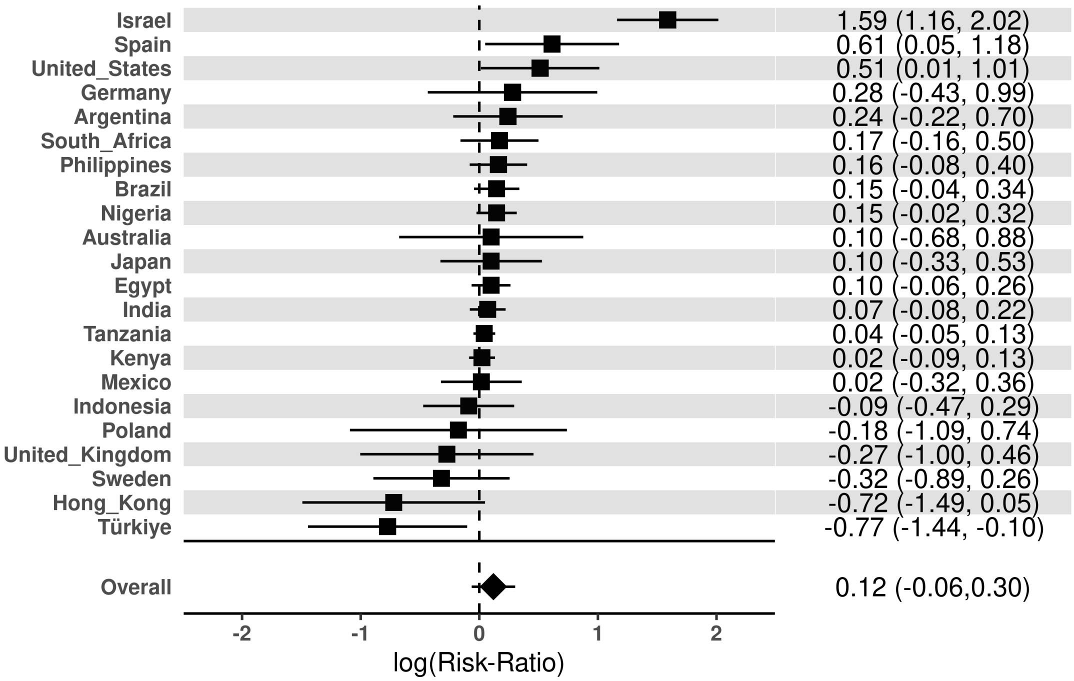

τ=0.376; Q-profile 95% CI [0.178, 0.538]; I^2=90.31;

Figure S15. Forest plot for `Immigration status`-`No` effect

Immigration status (Ref: Born in this country)

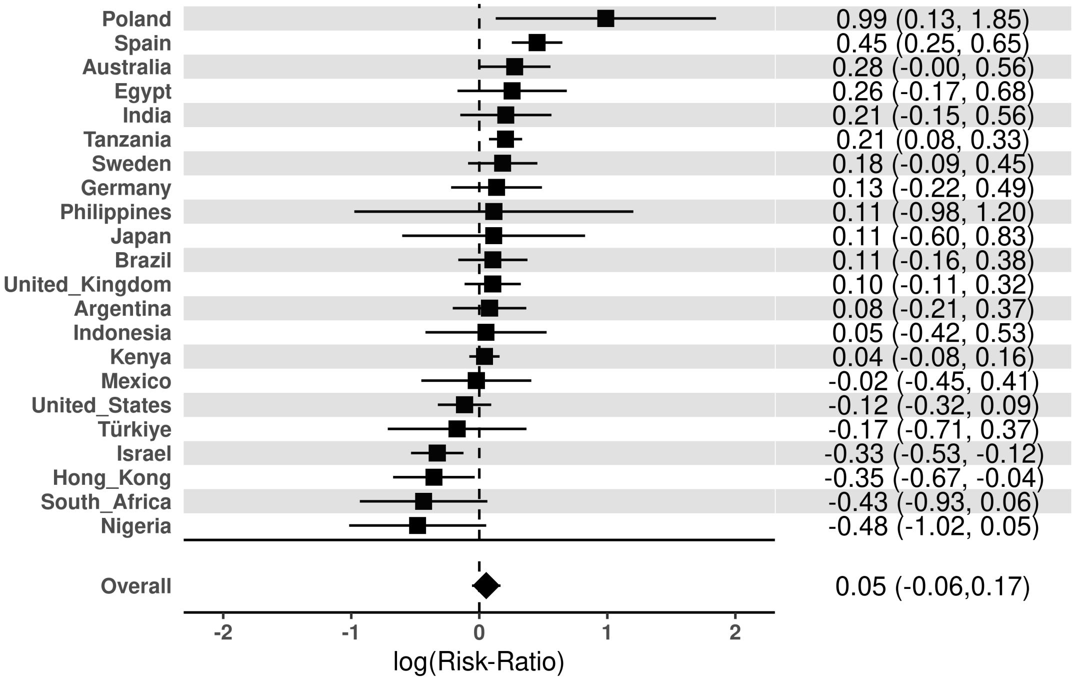

τ=0.198; Q-profile 95% CI [0.103, 0.308]; I^2=68.04;

Figure S16. Forest plot for `Age 12 religious service attendance`-`At least 1/week` effect

Age 12 religious service attendance (Ref: Never)

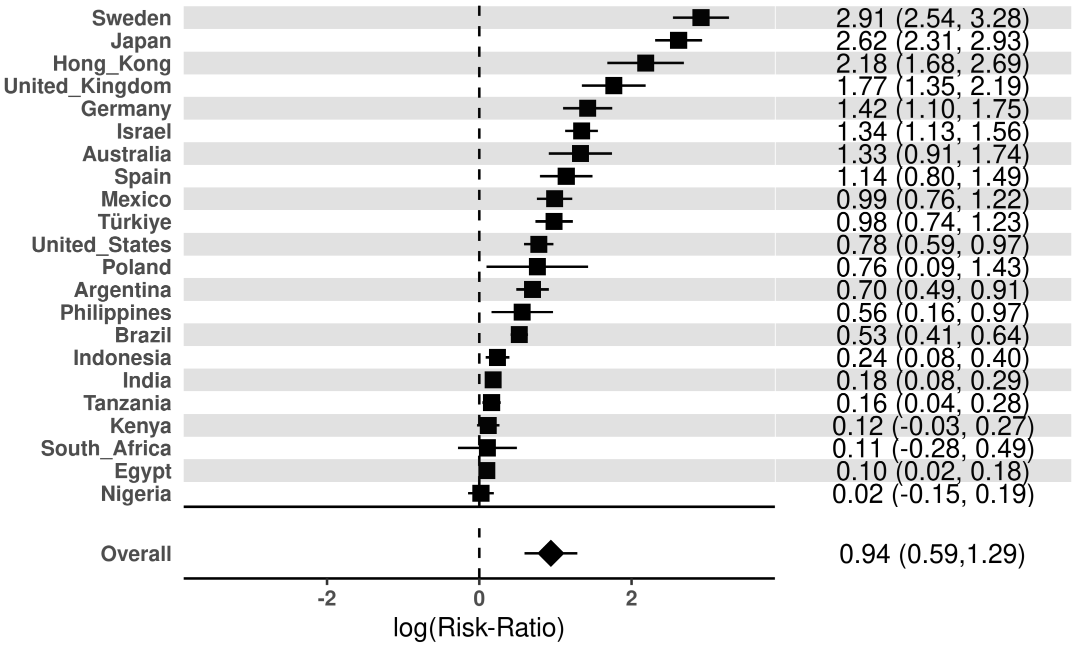

τ=0.815; Q-profile 95% CI [0.597, 1.115]; I^2=98.65;

Figure S17. Forest plot for `Age 12 religious service attendance`-`1-3/month` effect

Age 12 religious service attendance (Ref: Never)

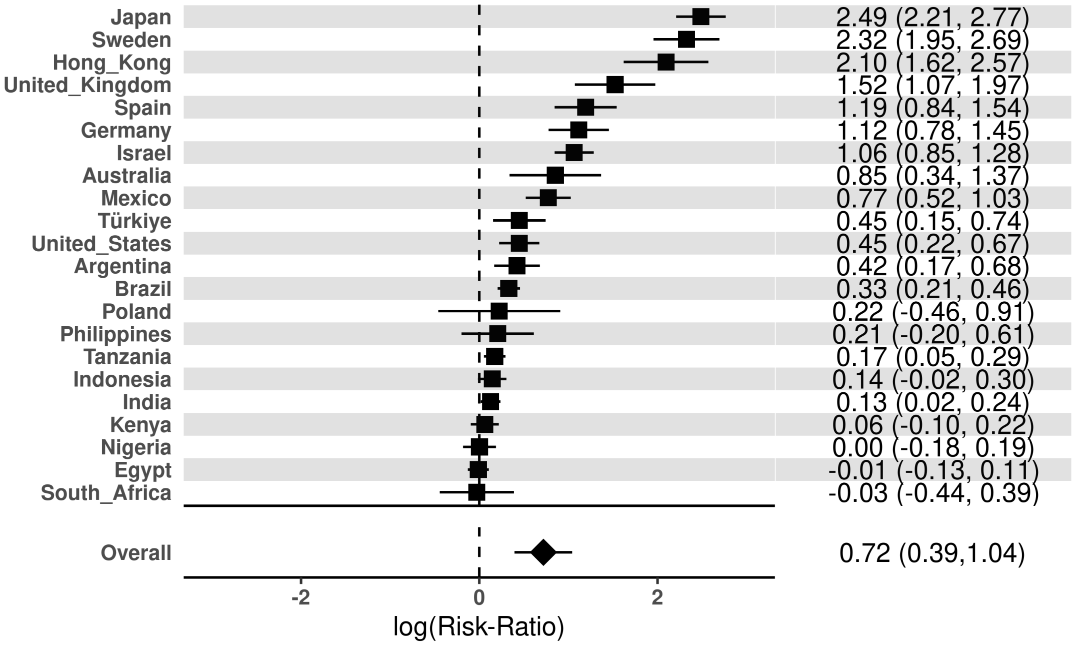

τ=0.757; Q-profile 95% CI [0.551, 1.037]; I^2=98.10;

Figure S18. Forest plot for `Age 12 religious service attendance`-`Less than 1/month` effect

Age 12 religious service attendance (Ref: Never)

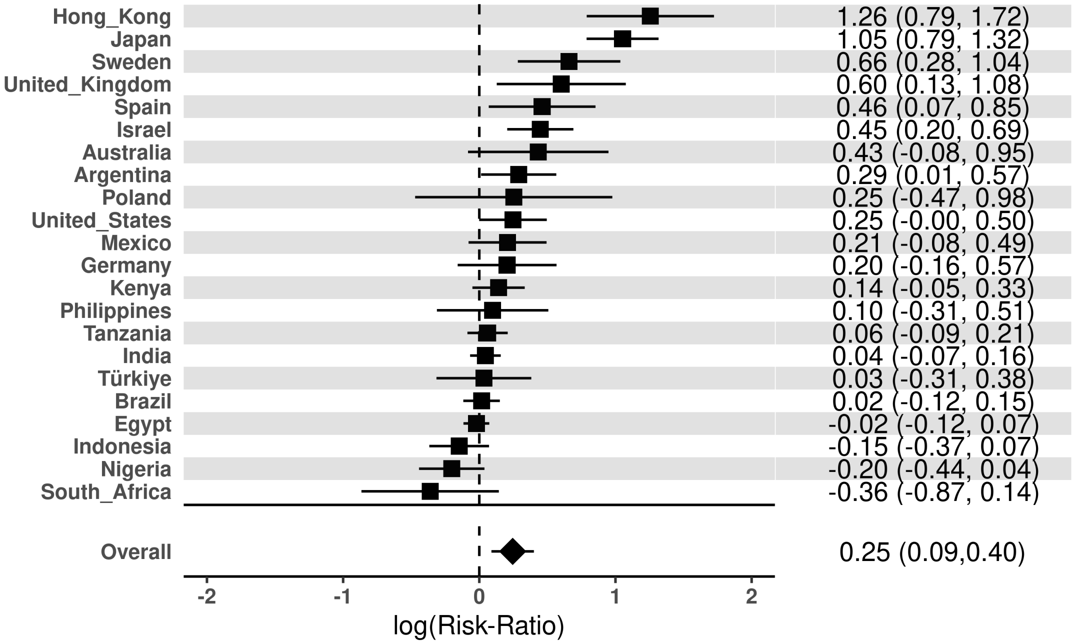

τ=0.334; Q-profile 95% CI [0.214, 0.474]; I^2=89.73;

Figure S19. Forest plot for `Gender`-`Female` effect

Gender (Ref: Male)

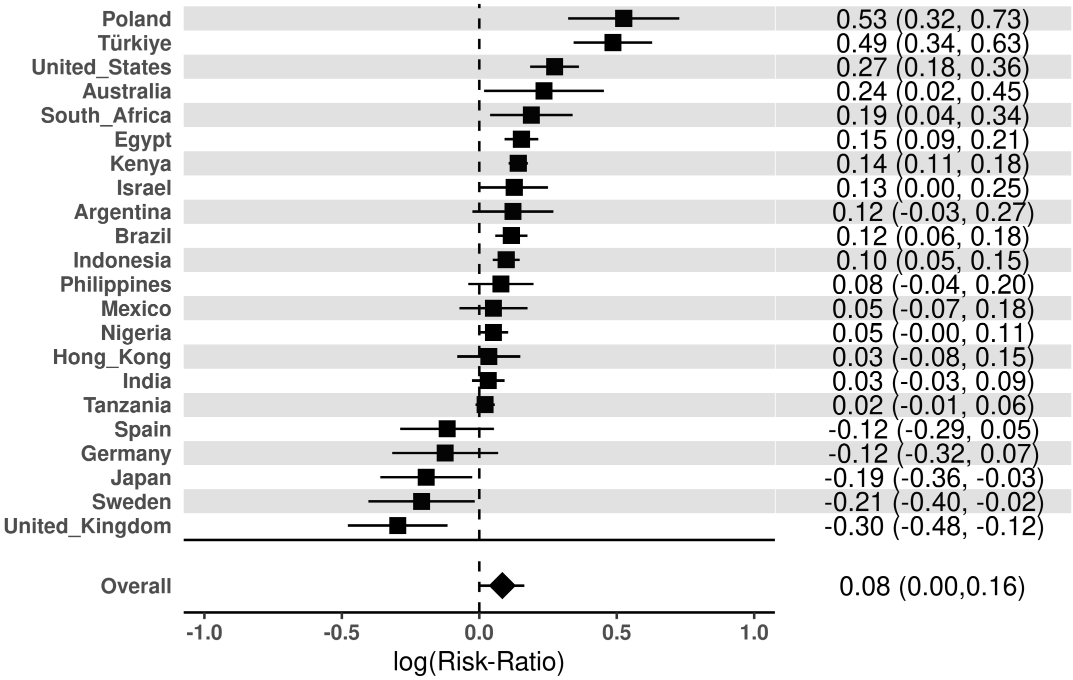

τ=0.180; Q-profile 95% CI [0.111, 0.246]; I^2=95.37;

Figure S20. Forest plot for `Gender`-`Other` effect

Gender (Ref: Male)

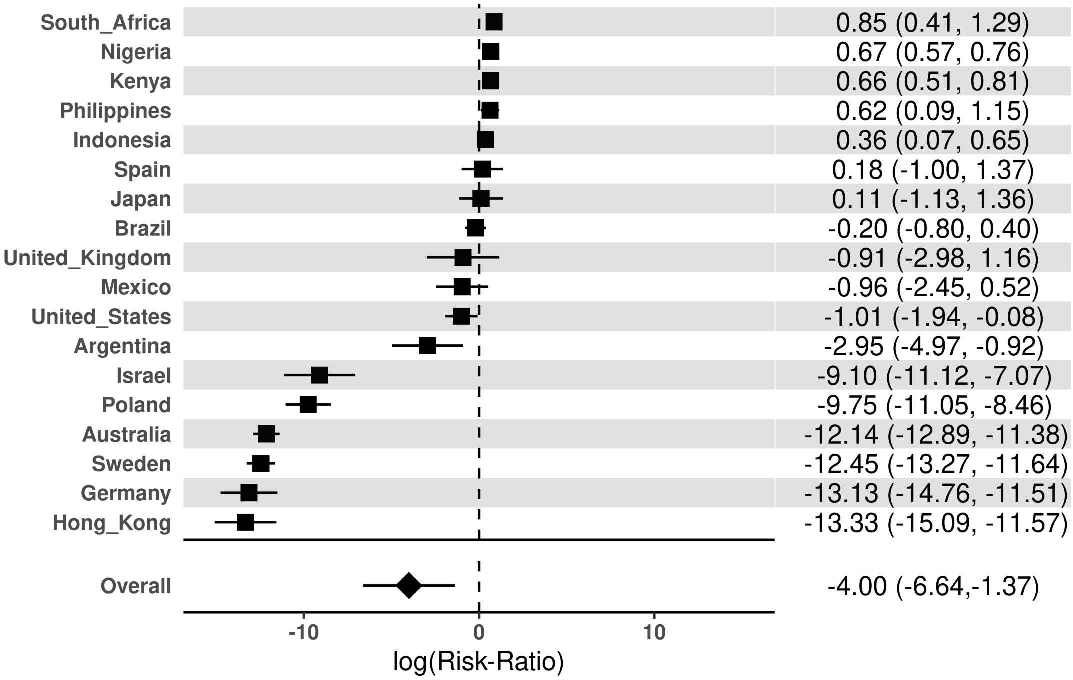

τ=5.663; Q-profile 95% CI [4.071, 7.976]; I^2=99.87;
Excluded countries: India, Egypt, Tanzania, Turkiye

Figure S21. Forest plot for `Year of birth`-`1993-1998; age 25-29` effect

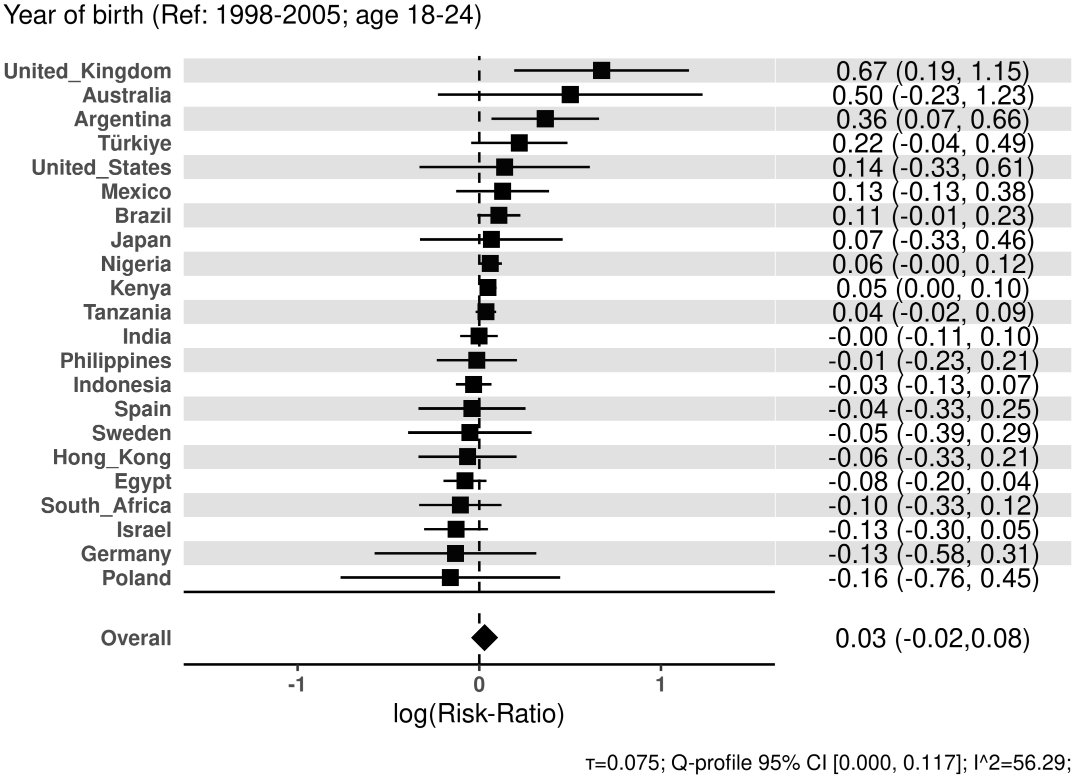

Figure S22. Forest plot for `Year of birth`-`1983-1993; age 30-39` effect

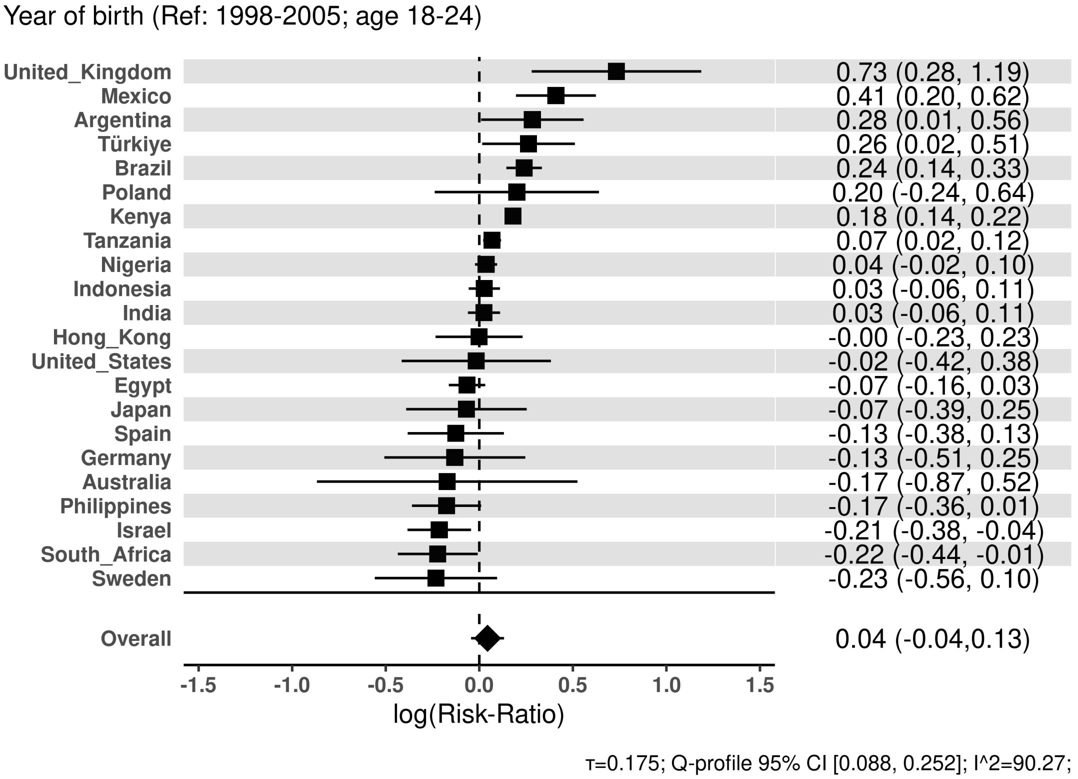

Figure S23. Forest plot for `Year of birth`-`1973-1983; age 40-49` effect

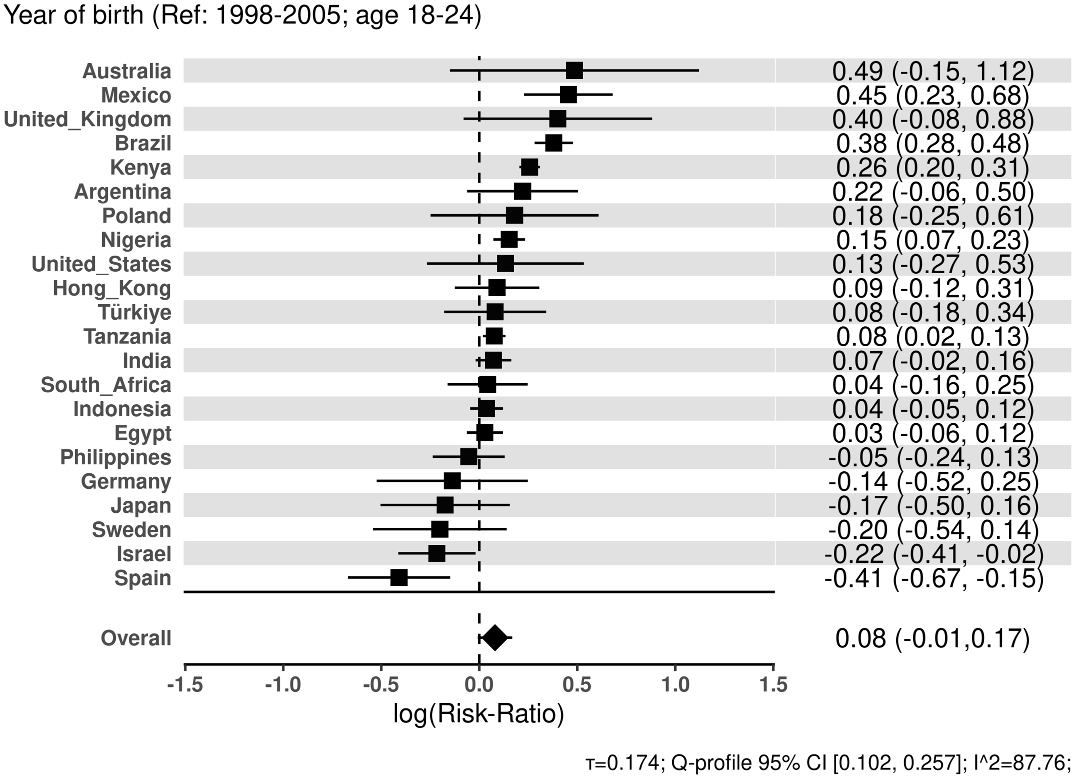

Figure S24. Forest plot for `Year of birth`-`1963-1973; age 50-59` effect

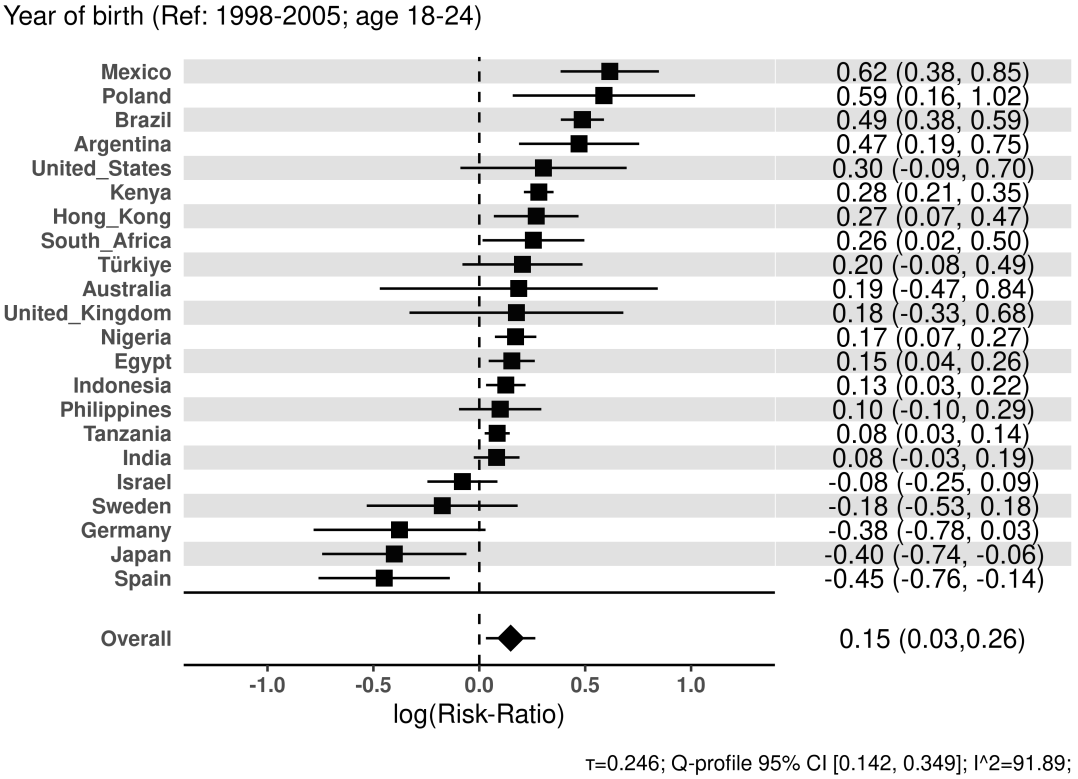

Figure S25. Forest plot for `Year of birth`-`1953-1963; age 60-69` effect

Year of birth (Ref: 1998-2005; age 18-24)

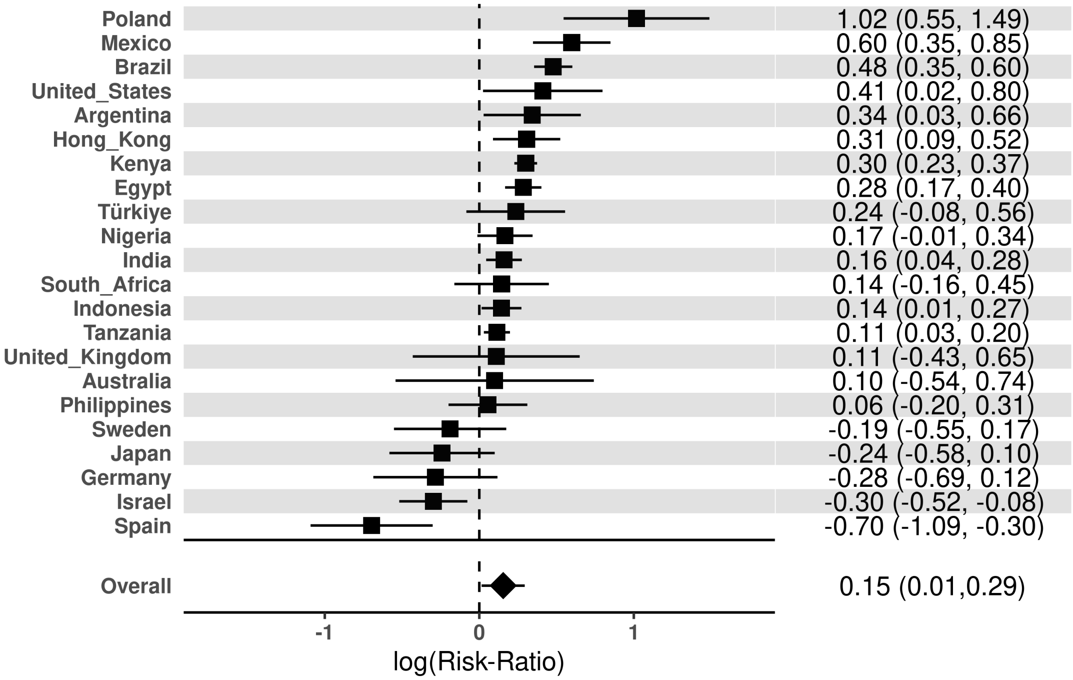

τ=0.299; Q-profile 95% CI [0.169, 0.418]; I^2=91.76;

Figure S26. Forest plot for `Year of birth`-`1943-1953; age 70-79` effect

Year of birth (Ref: 1998-2005; age 18-24)

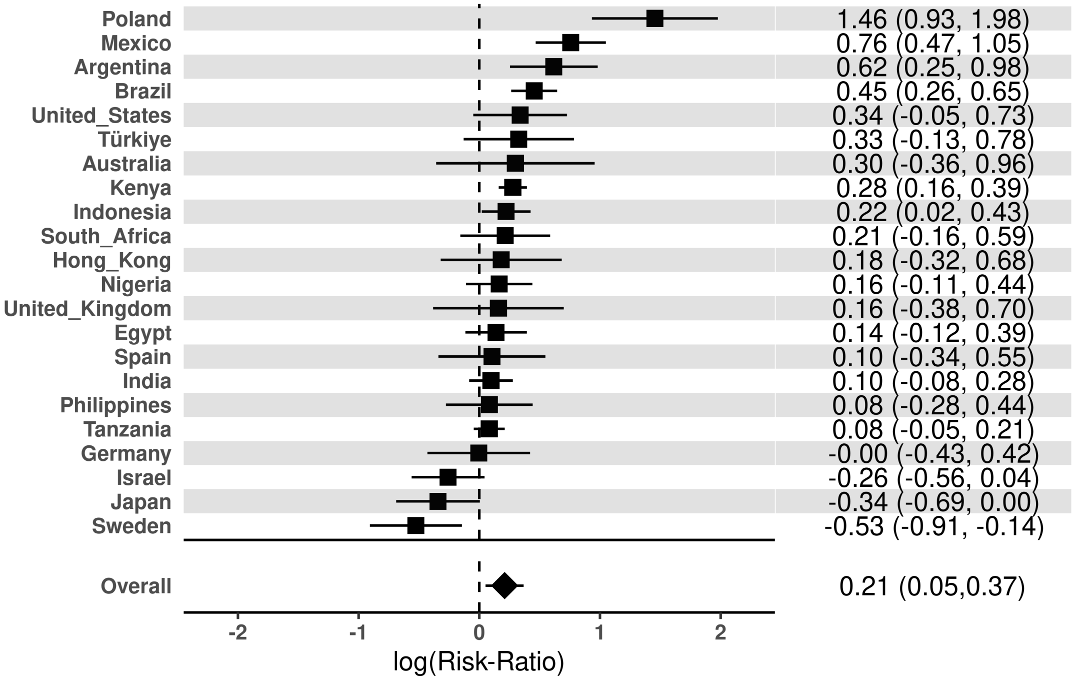

τ=0.332; Q-profile 95% CI [0.188, 0.470]; I^2=85.98;

Figure S27. Forest plot for `Year of birth`-`1943 or earlier; age 80+` effect

Year of birth (Ref: 1998-2005; age 18-24)

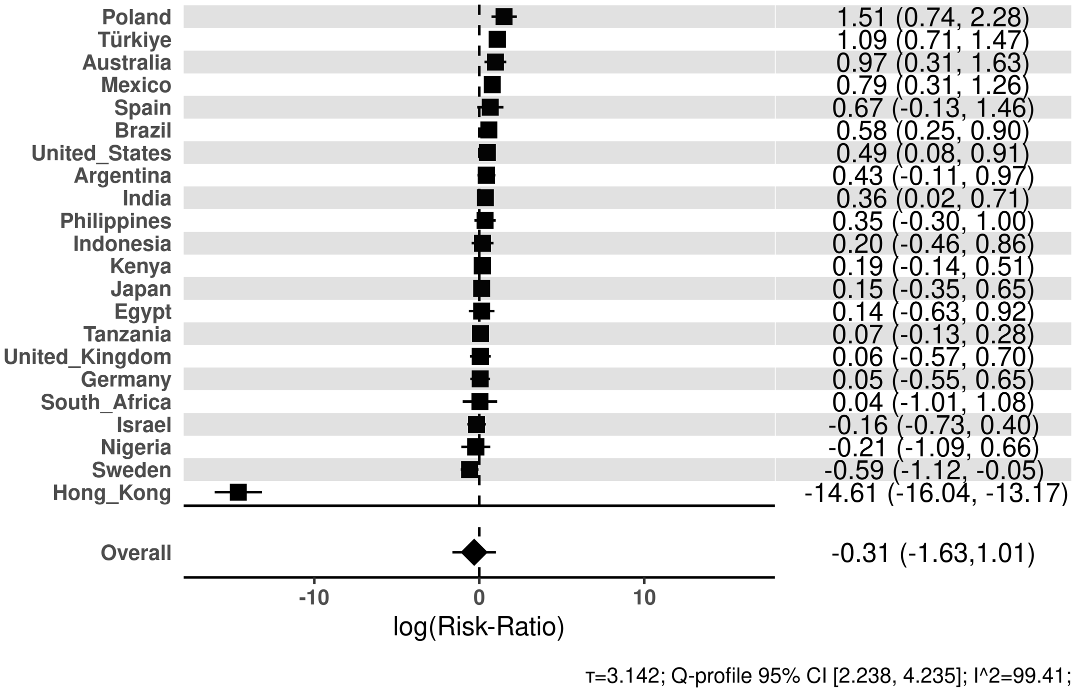

Supplement: Supplementary file 1 — Supplementary Material 1 [file 41598_2025_10142_MOESM1_ESM.pdf]
